# A Novel Contraception Counseling and Shared Decision-Making Curriculum for Internal Medicine Residents

**DOI:** 10.15766/mep_2374-8265.11046

**Published:** 2020-12-04

**Authors:** Rebeca Ortiz Worthington, Julie Oyler, Amber Pincavage, Nabil Abou Baker, Mark Saathoff, Jennifer Rusiecki

**Affiliations:** 1 Internal Medicine Resident, University of Chicago Medicine; 2 Associate Professor, Department of Medicine, University of Chicago Medicine; Associate Program Director, Internal Medicine Residency Program, University of Chicago Medicine; Associate Medical Director, Resident Clinic, Primary Care Group, University of Chicago Medicine; 3 Associate Professor, Department of Medicine, University of Chicago Medicine; Clerkship Director, Department of Medicine, University of Chicago Medicine; 4 Assistant Professor, Departments of Internal Medicine and Pediatrics, University of Chicago Medicine; 5 Director of Educational Technology and Learning for Clinical Skills Education, University of Chicago Pritzker School of Medicine; 6 Assistant Professor, Department of Medicine, University of Chicago Medicine; Women's Health Track Director, Internal Medicine Residency Program, University of Chicago Medicine

**Keywords:** Contraception, Contraception Counseling, Shared Decision-Making, Long-Acting Reversible Contraception (LARC), Family Planning, Patient-Centered Communication, Women's Health, Communication Skills, Primary Care

## Abstract

**Introduction:**

Many women of reproductive age with complex medical conditions receive primary care through an internal medicine (IM) physician rather than an obstetrician/gynecologist. Long-acting reversible contraception methods are the most effective form of contraception; however, IM residents are not routinely trained in them. Infrequent training in, inadequate knowledge of, and discomfort with contraception counseling limit the counseling performed by IM residents. Shared decision-making (SDM) is a method of patient-centered communication that can improve communication about patient preferences and increase patient satisfaction with and adherence to contraception. We developed a curriculum to teach contraception counseling under the framework of SDM for IM residents.

**Methods:**

The curriculum focused on contraception counseling through the lens of SDM designed for IM and medicine/pediatrics residents (PGY 2-PGY 4). We adapted an existing seven-step model of SDM to fit elements of contraception counseling. The curriculum consisted of a didactic teaching session with integration of an instructional video and structured interactive discussion. The session lasted 60 minutes.

**Results:**

Fifty-eight residents participated in the curriculum. On pre- and postcurriculum surveys, residents reported improvement in contraception knowledge (overall mean precurriculum = 57%, postcurriculum = 70%, *p* < .001) and comfort with contraception counseling (overall mean precurriculum = 3.2, postcurriculum = 3.6, *p* < .01). Residents expressed strong support for SDM before and after the curriculum.

**Discussion:**

Based on the survey results, the curriculum successfully addressed gaps in residents’ comfort with contraception counseling and knowledge of contraception side effects and efficacy.

## Educational Objectives

After participating in this curriculum, participants will be able to:

1. Counsel women on reproductive risk and efficacy of contraceptive options.
2. Identify medically complex female patients of reproductive age who are at risk for a medically complicated unintended pregnancy.
3. Identify contraindications to contraception.
4. Apply knowledge of contraception side effects and contraindications to medically complex female patients of reproductive age.
5. Identify teratogenic medications.
6. Employ the seven steps of shared decision-making when providing contraception counseling.
7. Confidently discuss long-acting contraception methods with patients and refer patients appropriately for placement of these options.

## Introduction

Between 2008 and 2011, the first decline in the rate of unplanned pregnancy in the United States occurred since 1981 and is largely attributed to the increased use of long-acting reversible contraception (LARC) methods.^1,2^ Studies have shown that contraceptive counseling in primary care visits is associated with increased contraceptive use.^3^ However, reproductive health services remain a small percentage of the issues addressed during primary care visits.^4^ In 2014, the Centers for Disease Control and Prevention (CDC) released a guideline stating that primary care physicians (PCPs) should provide contraceptive counseling and prescribe the selected method or refer the patient to a specialist for LARC placement.^4–6^ Internal medicine (IM) and medicine/pediatrics (med/peds) physicians are responsible for providing primary care to medically complex women of reproductive age, yet past studies have shown that inadequate knowledge is a barrier to contraception counseling.^7–10^ PCPs have reported numerous additional challenges with providing contraception counseling, including multiple medical problems that need to be addressed in a single visit, issues with insurance coverage, and limited access to placing or fitting certain forms of contraception.^11^ Women of reproductive age who receive their primary care from general internists, rather than obstetrician/gynecologists, are more likely to have chronic medical conditions that place them at higher risk of complications from unplanned pregnancy.^12^ It is crucial that PCPs are able to identify patients whose pregnancies would be high risk and prioritize contraception counseling for them.^11,13^

LARCs are the most effective forms of contraception, have few contraindications, and have increased adherence and continuation rates. Examples of LARC include hormonal and nonhormonal intrauterine devices (IUDs) and the subdermal implant (Nexplanon). However, LARC training is not a routine element of IM residency programs, and PCPs who practice independently or train residents have inaccurate perceptions of the effectiveness of LARCs.^12–14^ Prior studies have shown that medical students and trainees believe contraception is an important part of preventive care, but they report variable rates of contraception training, knowledge of the various options, and comfort with contraception counseling.^8,15,16^ The Contraceptive CHOICE Project demonstrated an increase in LARC prescribing with structured communication interventions, highlighting that counseling is an important factor in reproductive planning.^17,18^

Studies have shown that physicians make incorrect assumptions about which factors are most important to a patient when making medical decisions.^19^ Shared decision-making (SDM) is a method of patient-centered communication that can improve communication about patient preferences and increase patient satisfaction with and promote adherence to contraception.^20,21^ Despite this, one study of practicing physicians showed that when SDM occurred in clinic visits, it was only partially performed or not performed well.^22^ Research has shown that SDM allows patients to make a decision autonomously, with active involvement by the provider as the patient deliberates between her contraception options.^23^ Patients have expressed that during this process, they specifically want their providers to discuss contraception side effects—both negative and positive—to help them in their decision.^23,24^

We performed a needs assessment for this curriculum to teach IM and med/peds residents contraceptive counseling through the framework of SDM. This needs assessment of 38 PGY 2-PGY 4 residents at our institution from the academic year preceding the curriculum implementation demonstrated significant areas for improvement. Thirty-seven percent of residents felt they had not received adequate education or training about contraception. On a 5-point Likert scale (1 = *I need close supervision from a preceptor,* 5 = *I can teach this skill to others*), residents reported a median comfort level of 3 (interquartile range [IQR]: 2–3) for prescribing contraception. Residents also reported a median comfort level of 3 for discussing contraception efficacy rate (IQR: 2–3) and side effects (IQR: 2–3). The majority of residents identified that the hormonal implant, Mirena IUD, and tubal ligation were highly effective forms of birth control, but 73% of residents incorrectly stated that the Nexplanon was less effective than the Mirena IUD and tubal ligation. Fifty-nine percent of residents correctly identified that IUDs were safe for placement in patients with a history of PID. Fifty-four percent of residents correctly identified bleeding as a side effect of Nexplanon. Overall, residents scored 59% correct on the contraception knowledge questions.

We thus identified a need to train primary care IM residents to lead contraception counseling conversations with patients and to be prepared to discuss the details relevant to each patient's medical history. Residents reported discomfort with counseling and showed poor knowledge of contraception options and side effects. Focusing on LARCs and conversational counseling techniques using SDM, our curriculum was developed to address this training gap.

We searched for existing materials in PubMed and *MedEdPORTAL* using terms like *contraception counseling, shared decision-making, long-acting reversible contraception,* and *contraception counseling curriculum.* We were not able to locate any interactive or teaching modules teaching IM residents contraception counseling for medically complex patients via SDM. One resource in *MedEdPORTAL* teaches medical students contraceptive pharmacology; however, ours is aimed at IM residents rather than medical students and is not focused on pharmacology.^25^ A second publication teaches IM primary care residents in a military residency program about LARCs and the implant insertion, but it does not focus on SDM or identifying medically complex women who would be most impacted by pregnancy.^26^ Additionally, our curriculum is unique in that it utilizes a previously validated model of SDM to guide instruction of counseling techniques.

## Methods

The target population for this curriculum was IM and med/peds residents. Our sessions included PGY 2-PGY 4 residents, though PGY 1 trainees could also be included. PGY 1 trainees were not broadly included due to clinic and academic time constraints. The curriculum could also be used with faculty, medical students, and family medicine residents.

Before initiating the workshop, we identified program support, including protected space for the group session, audiovisual equipment for video production and viewing, and appropriate program staff to lead the session. Ideally, the individual leading the session would have familiarity with the resident continuity clinic(s).

The curriculum was delivered during dedicated lecture time in the residency program's ambulatory block schedule. The curriculum can be used as an independent curriculum or as an addition to an existing women's health or SDM curriculum. In our case, the session was part of a women's health curriculum taking place over 2 years and involving 10 didactic sessions lasting an hour each. The facilitator should be a core member of the residency program, including chief resident, core faculty, or associate program director.

This curriculum consisted of a didactic teaching session (Appendix I: PowerPoint Lecture) with the integration of an instructional video (Appendix D: Author-Owned Video) and structured discussion time. The session was approximately 60 minutes long, with 10 minutes of structured video viewing (Appendices D: Author-Owned Video and F: Video Observation Tool), 10 minutes of video debrief (Appendices E: Video Viewing Instructions and Questions and F: Video Observation Tool), and 40 minutes of didactic teaching of the seven-step model of SDM (Appendix H: 7 Steps of SDM for Contraception) integrated with contraception clinical information such as side effects and efficacy (Appendix I: PowerPoint Lecture). Residents were encouraged to ask questions throughout the session. A tiered efficacy contraceptive chart was used to organize the talk and to introduce the residents to tools for use with patients.^27^ The use of this chart was demonstrated in the clinical video. Residents were also provided with a Quick Start guide to oral contraception^28^ and a contraception dosing chart (Appendix G: Oral Contraceptive Dosing Chart).

The seven-step model was adjusted from a previously validated model of SDM for residencies to fit the unique elements of contraception counseling.^29^ An important addition to the adjusted model was reproductive risk. Residents were instructed to determine if an unexpected pregnancy would place the women or fetus at an elevated risk of pregnancy complications. If the patient was determined to be above average risk, the resident would discuss it with her in the equipoise step. For example, residents identified the need to discuss with the patient that she was taking a potentially teratogenic medication and therefore needed a highly effective form of contraception as part of her family planning. Residents related this to equipoise by discussing the idea that there were a few appropriate options for contraception and the physician needed the patient's input to make this decision.

An educational video (Appendix D: Author-Owned Video) was developed to demonstrate these communication skills in the clinical setting. We chose to include the video to concretely demonstrate specific communication skills around which our learners could focus their discussion and ask questions.^30^ This also helped create continuity across our several teaching sessions. The video was developed based on the data collected in the needs assessment and the seven-step SDM model. Our male residents ranked lower levels of comfort with this topic in the needs assessment compared to female residents; therefore, a male played the role of the doctor. Medical education and women's health experts vetted the script. We used a two-clinical scenario approach with a poor conversation and a better conversation. The poor conversation was viewed by the group at the start of the session, and the better conversation was watched after we had reviewed the curriculum content. Each video was debriefed as a large group.

To measure and trend trainee comfort, knowledge, and attitudes regarding contraception counseling, we used pre- and postworkshop surveys (Appendices A: Contraception SDM Presurvey, B: Contraception SDM Postsurvey, and C: Contraception SDM Survey Key). These surveys are optional. The precurriculum survey can be distributed at any point in time before initiating the curriculum. We distributed it after one of the other women's health teaching sessions. We found that distributing the postcurriculum survey immediately following the curriculum was most convenient, and we advise allocating enough time for participants to complete the survey at the end of the session.

The time line of an example education session (didactic content for each section is available in Appendix I: PowerPoint Lecture) is as follows:

• Introduce the concept of SDM (5 minutes).
• Start the educational video, viewing the initial poor conversation and debriefing it as a large group (10 minutes; Appendices D: Author-Owned Video, E: Video Viewing Instructions and Questions, and F: Video Observation Tool).
• Review the seven-step model of SDM (5 minutes; Appendix H: 7 Steps of SDM for Contraception).
• Throughout the seven steps of SDM, review contraceptive options with efficacy and side effects (30 minutes).
• Complete the educational video, viewing the second, better conversation and debriefing it as a large group (10 minutes; Appendices D: Author-Owned Video, E: Video Viewing Instructions and Questions, and F: Video Observation Tool).

## Results

This curriculum has been used with several groups of IM and med/peds PGY 2-PGY 4 residents, for a total of 58 residents at our institution during the 2017–2018 academic year, with a completion rate of 73%. The remaining residents were not present at the time of these teaching sessions. This publication's final author (Jennifer Rusiecki) facilitated the sessions.

After the curriculum, there was overall improvement in perceived barriers to contraception counseling (Table 1). On a 5-point Likert scale (1 = *I need close supervision from a preceptor,* 5 = *I can teach this skill to others*), residents reported an improvement in comfort with all topics assessed (overall mean comfort pre = 3.2, post = 3.6, *p* < .01). The greatest improvements were seen in comfort discussing contraception efficacy (pre = 2.8, post = 3.4, *p* < .01), patient perceptions/beliefs regarding contraception (pre = 3.5, post = 4.0, *p* < .01), and negotiating a contraceptive decision with the patient (pre = 3.4, post = 3.9, *p* < .01). Sixty-six percent of residents reported comfort with counseling patients on the Nexplanon subdermal implant after the curriculum, versus 37% previously. Gains were seen in most of the questions focused on medical knowledge and management (Table 2). Overall scores increased from 57% to 70% from pre- to postcurriculum, respectively (*p* < .001). There was a 26% increase in residents identifying Nexplanon as the most effective form of contraception (pre = 0%, post = 26%, *p* < .001). After the curriculum, 95% of residents correctly identified that patients with a history of PID could have an IUD placed, versus 59% previously (*p* = .28). Postcurriculum, 78% correctly identified the reason for avoiding combined hormonal contraceptives in patients with migraine with aura, versus 58% before the curriculum (*p* = .03).

**Table 1.**
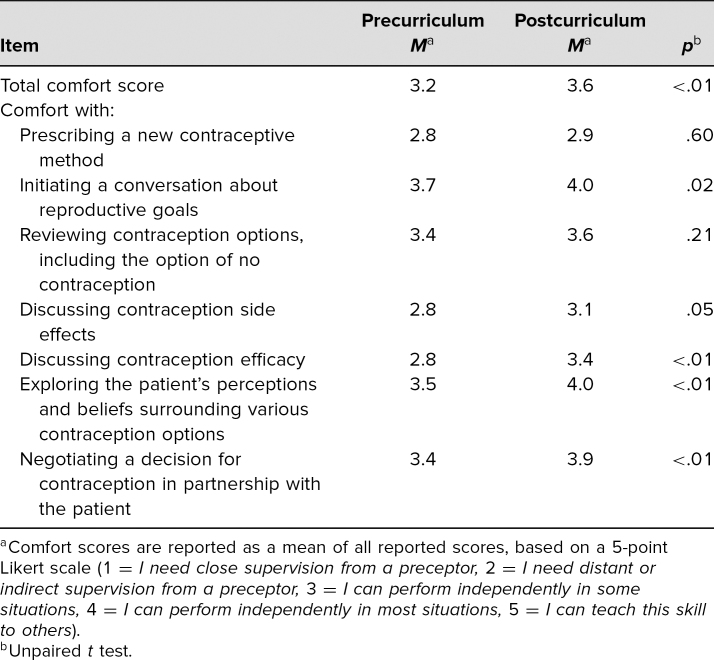
Resident Self-Reported Comfort With Contraception Counseling Topics on Pre- (*n* = 38) and Postcurriculum Surveys (*n* = 58)

**Table 2.**
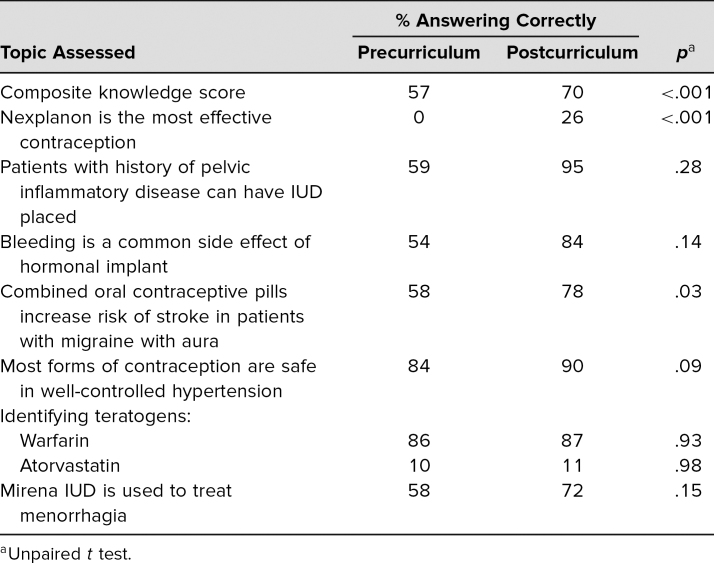
Percentage of Residents Answering Correctly on Pre- (*n* = 38) and Postcurriculum Surveys (*n* = 58)

Regarding the video portion of the curriculum, 95% of residents strongly agreed or agreed that the video scenarios helped highlight some negative elements of incomplete contraception counseling. Ninety percent strongly agreed or agreed that the video helped them feel more comfortable discussing contraception options with patients (see the Figure).

**Figure.**
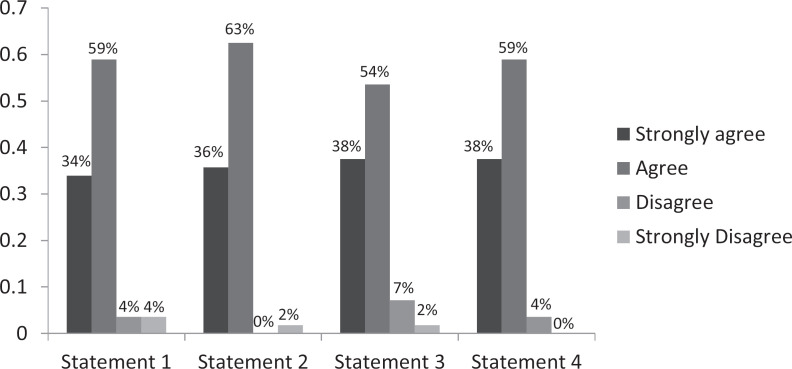
Postcurriculum video assessment (*n* = 56). Statement 1: After reviewing this video, I am more comfortable discussing contraception options with patients. Statement 2: The video scenarios help highlight some negative elements of incomplete contraception counseling. Statement 3: The video scenarios help highlight some possible strategies for improving my contraception counseling. Statement 4: I found reviewing the communication skills in this video as a group helpful.

Residents consistently expressed strong support for SDM, rating it as 4 on a 5-point Likert scale (1 = *Not at all,* 5 = *Extremely*) in terms of importance both before and after the curriculum (pre and post medians = 4, IQR: 4–5).

## Discussion

This curriculum addresses common knowledge gaps in residency primary care education. Before this curriculum, residents at our institution felt that contraception counseling was important but lacked knowledge and training to regularly integrate it into clinic visits. Based on the results of the surveys, the curriculum successfully addresses these gaps in comfort with contraception counseling and knowledge of contraception side effects and efficacy. The workshop is interactive and includes a variety of teaching modalities. In conjunction with the video, residents reported feeling better equipped to lead effective contraception counseling conversations with patients.

The unique benefit of the curriculum is its dual emphasis on contraception knowledge and the SDM communication technique. We held several small-group sessions when teaching the curriculum, which allowed for some variability in the details discussed during each session based on resident participation and questions. The video portion of the curriculum allowed for a consistent way to demonstrate leading an SDM session during a clinic visit, and residents reported this to be an effective teaching element.

The framework we present can be used by residents in a variety of clinical situations and can be adapted to individual patient and provider circumstances. Furthermore, we provide resources, such as the CDC contraception counseling guide,^27^ that can be easily accessed and reviewed with patients to facilitate these discussions. The precurriculum survey can be modified to assess several clinical knowledge scenarios, which can guide the creation of subsequent teaching sessions to fit the needs of a variety of resident groups and patient populations.

Based on these results, we will continue to use and improve upon this innovative curriculum in the future. It is not a comprehensive curriculum for identifying medications and medical conditions that would make a pregnancy high risk. Based on the results of this evaluation, we have added a more robust teratogen section to a different women's health session focused on pregnancy and preconception counseling to help resident better identify patients at high reproductive risk. The information in the contraception curriculum can be used to guide discussion of these scenarios with patients.

Although the postcurriculum data showed improvement in confidence with contraception prescribing, it did not reach statistical significance. Our study is likely underpowered given the small number of residents in our program. It may also highlight the logistical complexity of prescribing contraception. In future iterations of this curriculum, it may be helpful to dedicate time to prescribing or referring for placement within the electronic medical record.

Limitations of this curriculum evaluation include a single institution with a fixed number of resident participants. Due to time constrains, we were not able to include any direct observation of or feedback on residents’ communication skills. This evaluation does not include any direct patient input, and future work could incorporate patient surveys to identify and address patient-perceived gaps in clinical care, which would also help guide future changes to the curriculum. We have not been able to determine if the curriculum affected residents’ clinical practice and prescribing of contraception.

This curriculum is a novel way to teach contraception counseling, prescribing, and SDM in a streamlined format. Our evaluation demonstrates improvement in resident knowledge of and comfort with contraception and SDM. Next steps include adding an additional session to review teratogens and an institution-specific discussion of the prescribing and LARC referral process.

## Appendices

Contraception SDM Presurvey.docxContraception SDM Postsurvey.docxContraception SDM Survey Key.docxAuthor-Owned Video.movVideo Viewing Instructions and Questions.docxVideo Observation Tool.docxOral Contraceptive Dosing Chart.pdf7 Steps of SDM for Contraception.docxPowerPoint Lecture.pptx
All appendices are peer reviewed as integral parts of the Original Publication.
