# Supplementary material for: A Novel Contraception Counseling and Shared Decision-Making Curriculum for Internal Medicine Residents
Source: MedEdPORTAL. 2020 Dec 4;16:11046. doi: 10.15766/mep_2374-8265.11046 (PMC7727611; doi:10.15766/mep_2374-8265.11046)
Supplement: Supplementary file 1 — Contraception SDM Presurvey.docxContraception SDM Postsurvey.docxContraception SDM Survey Key.docxAuthor-Owned Video.movVideo Viewing Instructions and Questions.docxVideo Observation Tool.docxOral Contraceptive Dosing Chart.pdf7 Steps of SDM for Contraception.docxPowerPoint Lecture.pptx [file mep_2374-8265.11046-s001.zip › B. Contraception SDM Postsurvey.docx]

***Contraceptive Counseling and Shared Decision Making Survey***

This survey was created to gather information regarding your knowledge and experience with providing contraception counseling and practicing shared decision making during **continuity clinic** visits. Please answer to the best of your ability based on your current knowledge and experience. The survey should take roughly 10 minutes to complete.

IUD = intrauterine device, and refers to both the hormonal (Mirena) and copper (ParaGard) methods unless otherwise specified

**Section 1**

The following questions are related to your interests and attitudes regarding women’s health.

***How important to you is learning about contraception counseling during residency?***

a. Not at all b. Slightly c. Moderately d. Very e. Extremely

***How relevant is learning about contraception counseling to your career?***

a. Not at all b. Slightly c. Moderately d. Very e. Extremely

***Please indicate your comfort level with the following topics:***

| **Topic** | I need *close** supervision from a preceptor | I need *distant or indirect*** supervision from a preceptor | I can perform independently in *some**** situations | I can perform independently in *most* situations | I can teach this skill to others |
| --- | --- | --- | --- | --- | --- |
| ***Prescribing a new contraceptive method*** |  |  |  |  |  |
| ***Initiating a conversation about the patient’s reproductive goals*** |  |  |  |  |  |
| ***Reviewing contraception options including the option of no contraception*** |  |  |  |  |  |
| ***Discussing contraception side effects*** |  |  |  |  |  |
| ***Discussing contraception efficacy (i.e. failure rates)*** |  |  |  |  |  |
| ***Exploring the patient’s perceptions and beliefs surrounding various contraception options*** |  |  |  |  |  |
| ***Negotiate a decision for contraception in partnership with the patient*** |  |  |  |  |  |

**I need to observe my preceptor performing this skill*

***I need my preceptor to supervise me*

****I can simply discuss this with my preceptor*

***How important is shared decision making to you?***

a. Not at all b. Slightly c. Moderately d. Very e. Extremely

***What barriers, if any, have you encountered that affect your ability to counsel your patients on contraception? Select all that apply:***

1. I have not received adequate training or education about contraception
2. I do not feel comfortable talking with patients about reproductive health issues
3. I do not have time
4. I feel subspecialists are more skilled at these discussions
5. There are too many options available and I feel overwhelmed guiding the patient through this discussion
6. I do not feel the issue is as acute as the patient’s other medical concerns
7. I do not feel that this is a major focus or within the realm of my clinical practice
8. I infrequently need to counsel patients on contraceptive options
9. Contraception counseling is not included in the Epic “Best Practice Alert” or the health maintenance “dot phrase”
10. Other, please describe:

***Do you feel comfortable counseling a patient on the following contraceptive methods? Select all that apply:***

1. Condoms (male or female)
2. Combined hormonal contraceptives (pills, patch, or ring)
3. Progestin-only pills
4. Depot injections
5. Subdermal implants (Nexplanon)
6. Intrauterine device or system (Mirena or ParaGard IUD)
7. Other barrier contraceptives (diaphragms, cervical caps, spermicides)
8. Permanent sterilization (tubal ligation, vasectomy)
9. Periodic abstinence (natural family planning, “rhythm method”)
10. Abstinence
11. Withdrawal
12. Emergency contraception
13. None

**Section 2**

The following questions relate to your knowledge of contraceptive methods, side effects, contraindications.

***A patient and her husband feel their family is complete and do not desire any more children. Please rank the efficacy of the following methods (1=most effective, 5= least effective)***

- ____ Condoms (male or female) ____ Mirena IUD
- ____ Nexplanon Implant ____ Tubal Ligation
- ____ Combined hormonal contraceptives (pills, patch, or ring)

***A young woman who is a patient of yours has a history of pelvic inflammatory disease (PID). She continues to have unprotected intercourse with multiple partners. This patient should NOT have an IUD placed because it will further increase her risk for PID. This statement is:***

a. True b. False c. Not sure

***Which of the following is a common side-effect of the hormonal implant (e.g. Nexplanon)?***

1. Weight gain
2. Intermittent and unpredictable bleeding
3. Reduced efficacy of antibiotics
4. Any of the above
5. None of the above

***A 30 year-old never smoker with a history of migraine with aura is interested in starting Yaz (combined hormonal oral contraceptive pill). What is your concern?***

1. She may experience more severe or frequent migraines
2. This could interact with her sumatriptan, which she uses as a migraine abortive agent
3. Starting combined hormonal contraception greatly increases the risk of stroke in patients with migraines with aura
4. Starting ANY form of hormonal contraception greatly increases the risk of stroke in patients with migraines with aura
5. No concerns

***Women with well-controlled hypertension may be able to use which of the following contraceptive methods? Select all that apply:***

1. Combined hormonal contraceptives (pills, patch, or ring)
2. Progestin-only methods (pills or depot injections)
3. Intrauterine device or system
4. Emergency contraception
5. Any of the above
6. None of the above

***Which of the following medications is/are known to be teratogenic and require the effective use of contraception? Select all that apply:***

1. Isotretinoin (Accutane)
2. Metformin (Glucophage)
3. Acyclovir (Zovirax)
4. Warfarin (Coumadin)
5. Enalapril (Vasotec)
6. Sertraline (Zoloft)
7. Atorvastatin (Lipitor)
8. Hydrochlorothiazide
9. Not sure

***Which of the following forms of contraception may be used to improve menorrhagia? Select all that apply:***

1. Combined hormonal contraceptives (pills, patch, or ring)
2. Progestin-only methods (pills or depot injections)
3. Mirena IUD
4. ParaGard IUD
5. Hormonal implant (Nexplanon)
6. Any of the above
7. None of the above

**Section 3**

The following questions are related to your knowledge of shared decision making.

***Which ONE of the following is an element of shared decision making?***

1. Developing a partnership with other members of the health care team including RNs and LPNs to treat reproductive age women
2. Working towards increasing patient’s “change talk” regarding unprotected sex
3. Help the patient to make the most cost conscious choice when selecting between an IUD and oral contraceptive pills
4. Identifying the patients concerns and expectations of selecting a method of contraception

***Which ONE of the following defines the term “equipoise?”***

1. Multiple possible answers without one clearly superior choice
2. The act of interpreting evidenced based medical terms for a patient
3. The point where evidenced based medicine and patient-centered communication skills meet
4. The process of discussing high value, cost conscious care with patients

***Shared decision making should be used in which ONE of the following clinical situations?***

1. When a patient does not take her oral contraceptive pills every day
2. For counseling a patient regarding frequency of mammogram screening
3. Providing patient education on safe sex practices
4. Prescribing a short course of antibiotics for a UTI

**Section 4**

The following statements are about the contraception counseling video.

***After reviewing this video I am more comfortable discussing contraception options with patients.***

1. Strongly Agree b. Agree c. Disagree d. Strongly Disagree

***The video scenarios were realistic and believable.***

1. Strongly Agree b. Agree c. Disagree d. Strongly Disagree

***I found reviewing the communication skills in this video as a group helpful.***

1. Strongly Agree b. Agree c. Disagree d. Strongly Disagree

**Section 5**

The following are demographic questions.

***What is your gender?***

a. Male b. Female c. Self-describe: ­­­­­__________________

***What is you training specialty?***

a. Internal Medicine b. Medicine/Pediatrics

***What is your PGY level?***

a. PGY1 b. PGY2 c. PGY3 d. PGY4

***My long-term career plan involves:***

a. Primary Care b. Subspecialty c. Hospitalist d. Primarily Research e. Primarily Teaching f. Other

*Thank you for your participation!*
