# Supplementary material for: A Novel Contraception Counseling and Shared Decision-Making Curriculum for Internal Medicine Residents
Source: MedEdPORTAL. 2020 Dec 4;16:11046. doi: 10.15766/mep_2374-8265.11046 (PMC7727611; doi:10.15766/mep_2374-8265.11046)
Supplement: Supplementary file 1 — Contraception SDM Presurvey.docxContraception SDM Postsurvey.docxContraception SDM Survey Key.docxAuthor-Owned Video.movVideo Viewing Instructions and Questions.docxVideo Observation Tool.docxOral Contraceptive Dosing Chart.pdf7 Steps of SDM for Contraception.docxPowerPoint Lecture.pptx [file mep_2374-8265.11046-s001.zip › E. Video Viewing Instructions and Questions.docx]

**Contraception Counseling and Shared Decision Making**

**Video Instructions for Facilitators**

**How to use this video**:

This video was developed for IM and Med-Peds residents but can be used with medical students, housestaff in other specialties, midlevel providers, and faculty. This video includes 2 conversations demonstrating a “poorly” and “better” preformed conversation. The “poorly” performed conversation can be utilized at the start of the session as a way to develop buy-in from the learners. The “better” conversation can be viewed either immediately following the "poorly" performed conversation or after the didactic component of the curriculum.

The video will start with a typed description of the clinical scenario which can be used to orient the learners to the video. The video can either be debriefed after the video or can be paused throughout for comments. We watched each of the conversations without pause and then debriefed each conversation as a large group. We also used the attached viewing tool to encourage the learners to take notes while actively viewing the video.

**Video Learning Objectives**:

By viewing this video learners will be able to:

1. Identify 2 areas of the conversation that could be improved and offer suggestions for improvement.
2. Identify the 7-steps of shared decision making and 2 communication skills used in the video.
3. Discuss reproductive risk with a medically complicated female patient of reproductive age.

Debriefing Questions:

1. What do you feel went well in that conversation?
2. What do you feel could be improved in the conversation?
3. Which steps of shared decision making did you see used in this conversation?
4. What communication skills were used in this conversation? (Example skills: agenda-setting, teach-back, Ask/Tell/Ask, summarizing, etc)
5. How can you integrate this into your clinical practice?
6. What challenges do you see with integrating this into your clinical practice?

Further Talking Points:

Conversation 1:

1. Based on the intro prompt what are some things that you want to discuss with this patient?
   1. *We want to assess her family planning goals. Using questions like the one key question: “Are you interested in becoming pregnant in the next year?” is a good option.*
   2. *We also need to gauge her understanding of the risk of methotrexate in pregnancy. Has anyone discussed the teratogen risk with this medication before?*
   3. *We need to discuss the effectiveness of condoms for birth control and see how she feels about that. This can be used as a bridge to discussing a more effective contraception option.*
2. How do you think the patient felt when the doctor said: “You don’t want to get pregnant right?”
   1. *As health care providers we need to move away from the idea that unexpected pregnancies are a universally unwanted outcome. To discuss contraception with patients we first need to gauge their reproductive goals. The doctor in the video comes off belittling the patient instead of building a therapeutic relationship with her. A better choice would be to use the PATH or One Key Question to assess reproductive goals.*
3. How confident do you feel that this patient will make her follow up visit with the women’s clinic?
   1. *Women who discuss contraception with their PCP are more likely to be compliant with their chosen method.1 The patient may feel brushed off if their provider does not explore their values and concerns surrounding contraception. Even if we are referring a patient to another provider for LARC placement or further counseling it is important that we still perform the steps of shared decision making with the patient.*

Conversation 2:

1. How do you think the discussion around the risk of methotrexate in pregnancy went?
   1. *When discussing potentially dangerous side effects of medication it is important to not scare the patient. We want her to be informed about the risk but she needs to treat her rheumatoid arthritis. It is important that we inform women that they can still become pregnant and have a healthy pregnancy (depending on condition) despite their chronic medical conditions. It is helpful to put the focus on planning and prevention when discussing the reproductive risk.*
2. What have we learned about the patient’s values and concerns with contraception?
3. *Through this conversation, we have learned that a weight stable option is important to her. We have also learned that she has little confidence in her ability to take daily medication and is comfortable with amenorrhea. It is important to collect this information and use it to guide the conversation. You do not need to discuss an option if it contrasts her values.*
4. How did the doctor due to explaining the contraception options chart?
5. *The doctor did a good job of orienting her to the chart and explain what the number on the chart means. Something he could improve on is not using words like “better” to describe the more effective options. We do not want patients to feel pressured to use one of these options. A more patient-center choice would be to say “the options at the top are more effective or have less unexpected pregnancies than the options at the bottom.”*
6. What skills did the doctor use to move the conversation forward in a timely manner?
7. *The doctor in this video did a good job of exploring the patient’s values on this topic and editing the conversation based on this. We did not discuss the risk and benefit of Depo-Provera since she told us she does not want to gain weight. With each option discussed, he gave 1-2 benefits and risks with each option. You do not need to give an exhaustive list of side effects for every option. When you decide on a method you can review the side effects further. Toward the end of the visit, he used summarizing to help focus the patient on a final decision.*

References:

1. Lee JK, Parisi SM, Akers AY, Borrerro S, Schwarz EB. The impact of contraceptive counseling in primary care on contraceptive use. *J Gen Intern Med*. 2011;26(7):731-736. <https://doi.org/10.1007/s11606-011-1647-3>
