# Supplementary material for: A Novel Contraception Counseling and Shared Decision-Making Curriculum for Internal Medicine Residents
Source: MedEdPORTAL. 2020 Dec 4;16:11046. doi: 10.15766/mep_2374-8265.11046 (PMC7727611; doi:10.15766/mep_2374-8265.11046)
Supplement: Supplementary file 1 — Contraception SDM Presurvey.docxContraception SDM Postsurvey.docxContraception SDM Survey Key.docxAuthor-Owned Video.movVideo Viewing Instructions and Questions.docxVideo Observation Tool.docxOral Contraceptive Dosing Chart.pdf7 Steps of SDM for Contraception.docxPowerPoint Lecture.pptx [file mep_2374-8265.11046-s001.zip › F. Video Observation Tool.docx]

**Contraception Counseling Observation Tool**

**Date: _________**

**Name: ___________________________**

**Video 1**

| Steps | Performed well | Performed poorly | Not performed |
| --- | --- | --- | --- |
| Identify reproductive goals |  |  |  |
| Explaining equipoise |  |  |  |
| Explain Reproductive risk |  |  |  |
| Explore pt’s values |  |  |  |
| Review options |  |  |  |
| Check understanding |  |  |  |
| Negotiate a decision |  |  |  |
| Review treatment plan |  |  |  |

What went well:

Areas for improvement:

**Video 2**

| Steps | Performed well | Performed poorly | Not performed |
| --- | --- | --- | --- |
| Identify reproductive goals |  |  |  |
| Explaining equipoise |  |  |  |
| Explain Reproductive risk |  |  |  |
| Explore pt’s values |  |  |  |
| Review options |  |  |  |
| Check understanding |  |  |  |
| Negotiate a decision |  |  |  |
| Review treatment plan |  |  |  |

What went well:

Areas for improvement:
