# Supplementary material for: A Novel Contraception Counseling and Shared Decision-Making Curriculum for Internal Medicine Residents
Source: MedEdPORTAL. 2020 Dec 4;16:11046. doi: 10.15766/mep_2374-8265.11046 (PMC7727611; doi:10.15766/mep_2374-8265.11046)
Supplement: Supplementary file 1 — Contraception SDM Presurvey.docxContraception SDM Postsurvey.docxContraception SDM Survey Key.docxAuthor-Owned Video.movVideo Viewing Instructions and Questions.docxVideo Observation Tool.docxOral Contraceptive Dosing Chart.pdf7 Steps of SDM for Contraception.docxPowerPoint Lecture.pptx [file mep_2374-8265.11046-s001.zip › G. Oral Contraceptive Dosing Chart.pdf]

# Oral Contraceptive Dosing Chart

Adapted by: Jennifer Rusiecki, MD, MS

| Estrogen (mcg) | Progestin (mg) | Progesterone | Brands |
|---|---|---|---|
| 10 | 1 | Norethindrone | Lo loestrin |
| 20 | 3 | Drospirenone | Yaz |
| | 0.1 | levonorgestrel | Aviane 28, lessina 28 |
| | 1 | Norethindrone | Gildess, Junel 1/20, microgestin, loestrin |
| 25 | 0.18,0.125, 0.25 | Norgestimate | Ortho Tri-Cyclen Lo |
| 30 | 0.15 | Desogestrel | Apri 28, Ortho-cept 28 |
| | 3 | Drospirenone | Yassmin, ocella 28 |
| | 0.15 | levonorgestrel | Atavera 28, levora 28 |
| | 1.5 | Norethindrone | Gildess 1.5/30, Junel 1.5/30, microgestin 1.5/30 |
| 35 | 0.5 | Norethindrone | Brevicon, Necon 0.5/25, nortrel 0.5/35 |
| | 1 | Norethindrone | Alyacen 1/35, Necon 1/35, Norinyl 1+35, Ortho-Novum 1/35 |
| | 0.25 | Norgestimate | Ortho-Cyclen 28, Sprintec 28, MonoNessa 28 |
| | 0.18, 0.215,0.25 | Norgestimate | Ortho Tri-Cyclen 28, TriNessa 28, Tri-Sprintec 28 |

Common 1st regimens

Adjusted from Allen R. Combined estrogen-progestin oral contraceptives: Patient selection, counseling, and use. Table 1. UpToDate. Waltham, MA: UpToDate Inc. https://www.uptodate.com. (Accessed on October 10, 2019)
