# Supplementary material for: A Novel Contraception Counseling and Shared Decision-Making Curriculum for Internal Medicine Residents
Source: MedEdPORTAL. 2020 Dec 4;16:11046. doi: 10.15766/mep_2374-8265.11046 (PMC7727611; doi:10.15766/mep_2374-8265.11046)
Supplement: Supplementary file 1 — Contraception SDM Presurvey.docxContraception SDM Postsurvey.docxContraception SDM Survey Key.docxAuthor-Owned Video.movVideo Viewing Instructions and Questions.docxVideo Observation Tool.docxOral Contraceptive Dosing Chart.pdf7 Steps of SDM for Contraception.docxPowerPoint Lecture.pptx [file mep_2374-8265.11046-s001.zip › H. 7 Steps of SDM for Contraception.docx]

Appendix C

Seven Steps of Shared Decision Making for Contraception


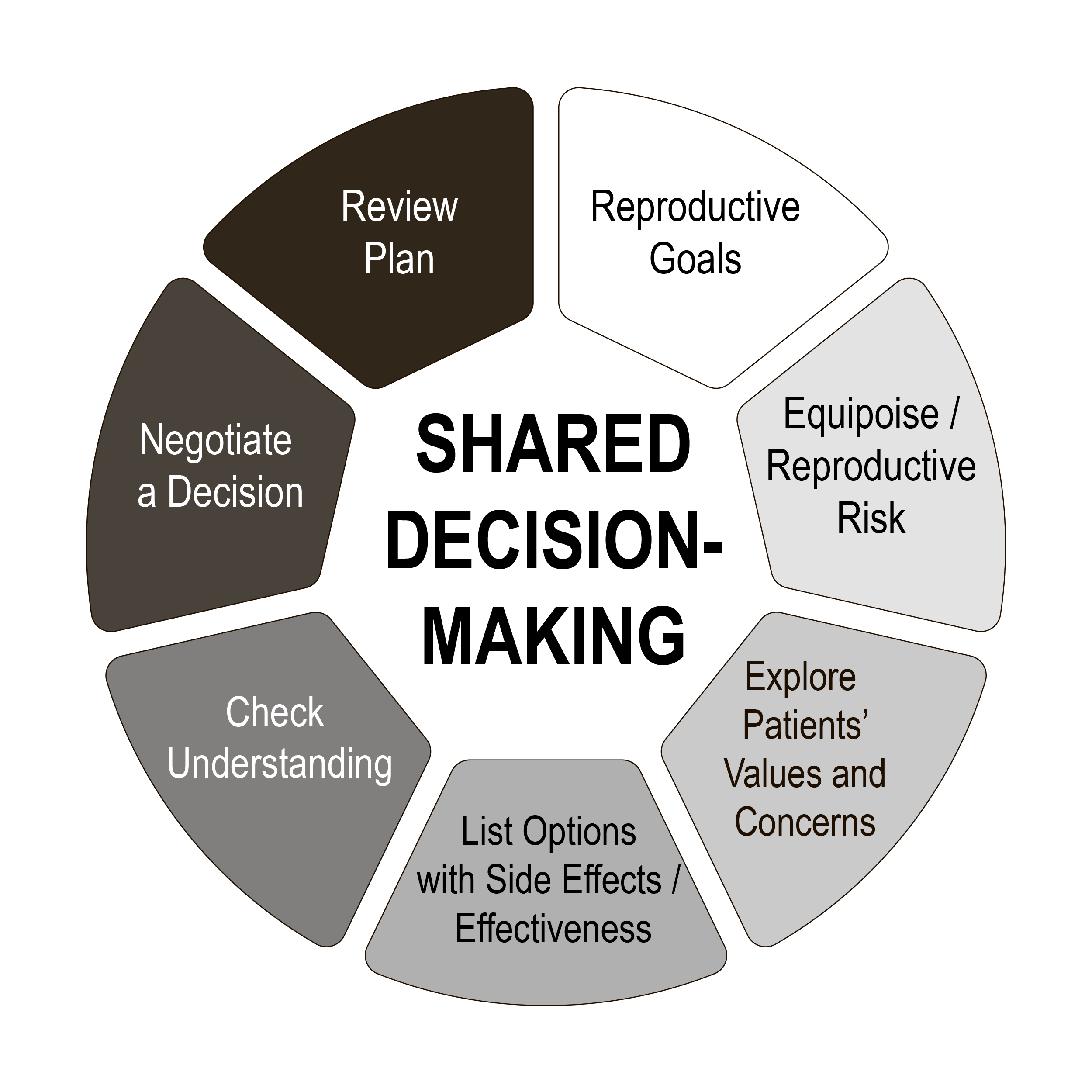


Example verbiage for each step:

Reproductive Goals – *Is pregnancy something you are interested in over the next year?*

Equipoise/Reproductive Risk – *Pregnancy can be a more complicated for women with lupus. Women with lupus can have healthy pregnancies it just takes a little more planning therefore we need to discuss birth control. The good news is we have many birth control options and I need your input to find the best option.*

Explore the Patient’s Values/Concerns – *Tell me what you have heard about birth control?*

*Is having a regular period something that is important to you?*

*What is important to you in your birth control?*

List Options with Effectiveness and Side Effects – If using a chart: *This is a chart of all the options for birth control. The more effective options or those with less unplanned pregnancies are at the top. The less effective options or those with more unplanned pregnancies are at the bottom.*

*The implant is very effective at preventing pregnancies. Most women have lighter less frequent periods with this method. The most common side effect is unexpected bleeding and about a third of women have it removed due to this.*

Check Understanding – *We have covered a lot of information and I just want to make sure it was clear. Can you tell me what you understood from our discussion?*

*What questions do you have?*

Negotiate a Decision – *We have discussed a lot of options, tell me what you are thinking?*

*I hear that you are interested in a method that will make your periods less frequent and you do not want to take a daily medication which leaves us with the IUD and the ring. Is that right?*

Review Plan – *I will place the consult to gynecology for the IUD placement and the front desk will help you schedule the appointment. Please continue to use condoms to prevent STDs and pregnancy.*

Citation: Author owned
