# Supplementary material for: A Novel Contraception Counseling and Shared Decision-Making Curriculum for Internal Medicine Residents
Source: MedEdPORTAL. 2020 Dec 4;16:11046. doi: 10.15766/mep_2374-8265.11046 (PMC7727611; doi:10.15766/mep_2374-8265.11046)
Supplement: Supplementary file 1 — Contraception SDM Presurvey.docxContraception SDM Postsurvey.docxContraception SDM Survey Key.docxAuthor-Owned Video.movVideo Viewing Instructions and Questions.docxVideo Observation Tool.docxOral Contraceptive Dosing Chart.pdf7 Steps of SDM for Contraception.docxPowerPoint Lecture.pptx [file mep_2374-8265.11046-s001.zip › I. PowerPoint Lecture.pptx]

## Slide 1
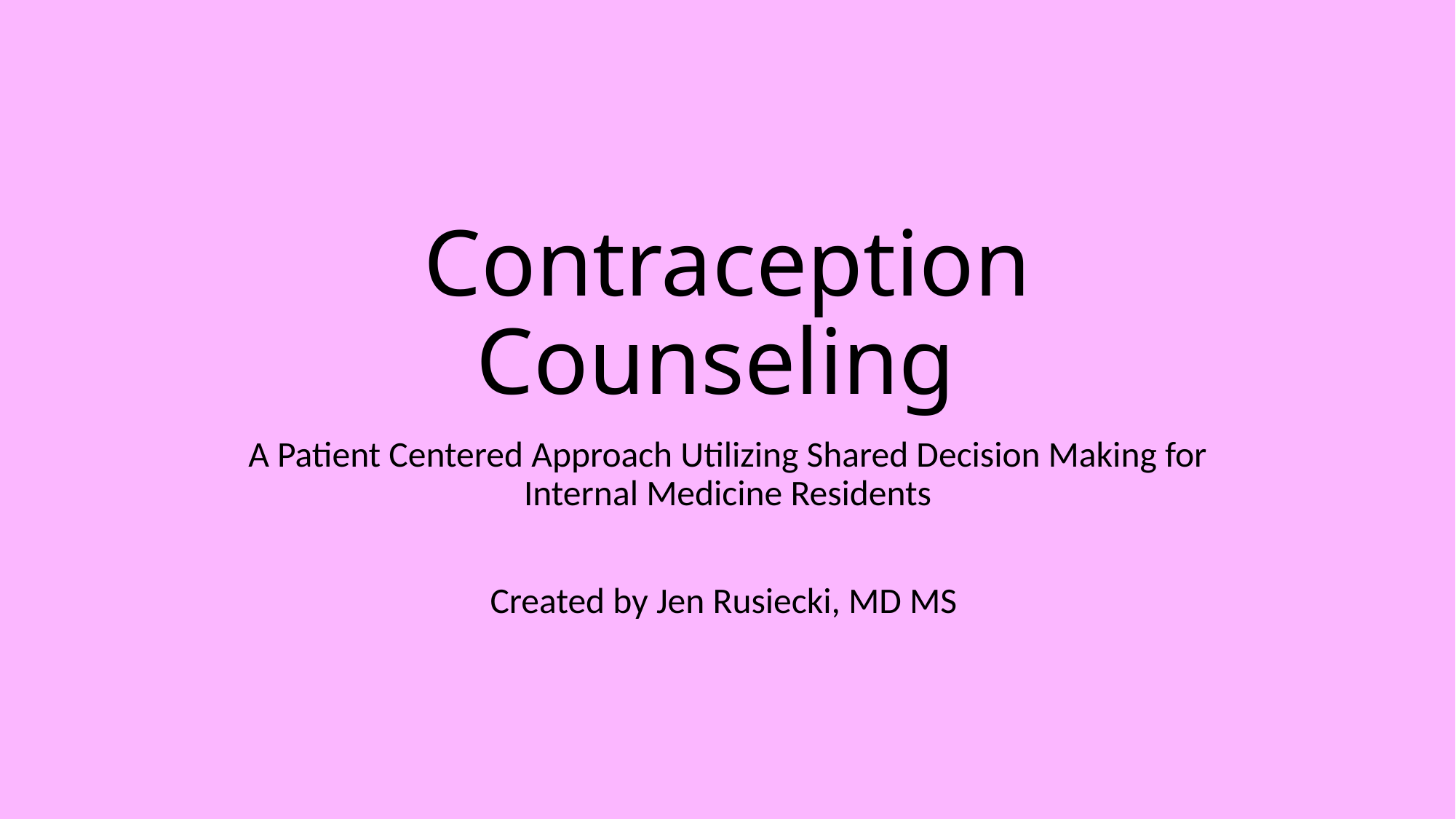

# Contraception Counseling
A Patient Centered Approach Utilizing Shared Decision Making for Internal Medicine Residents
Created by Jen Rusiecki, MD MS

## Slide 2
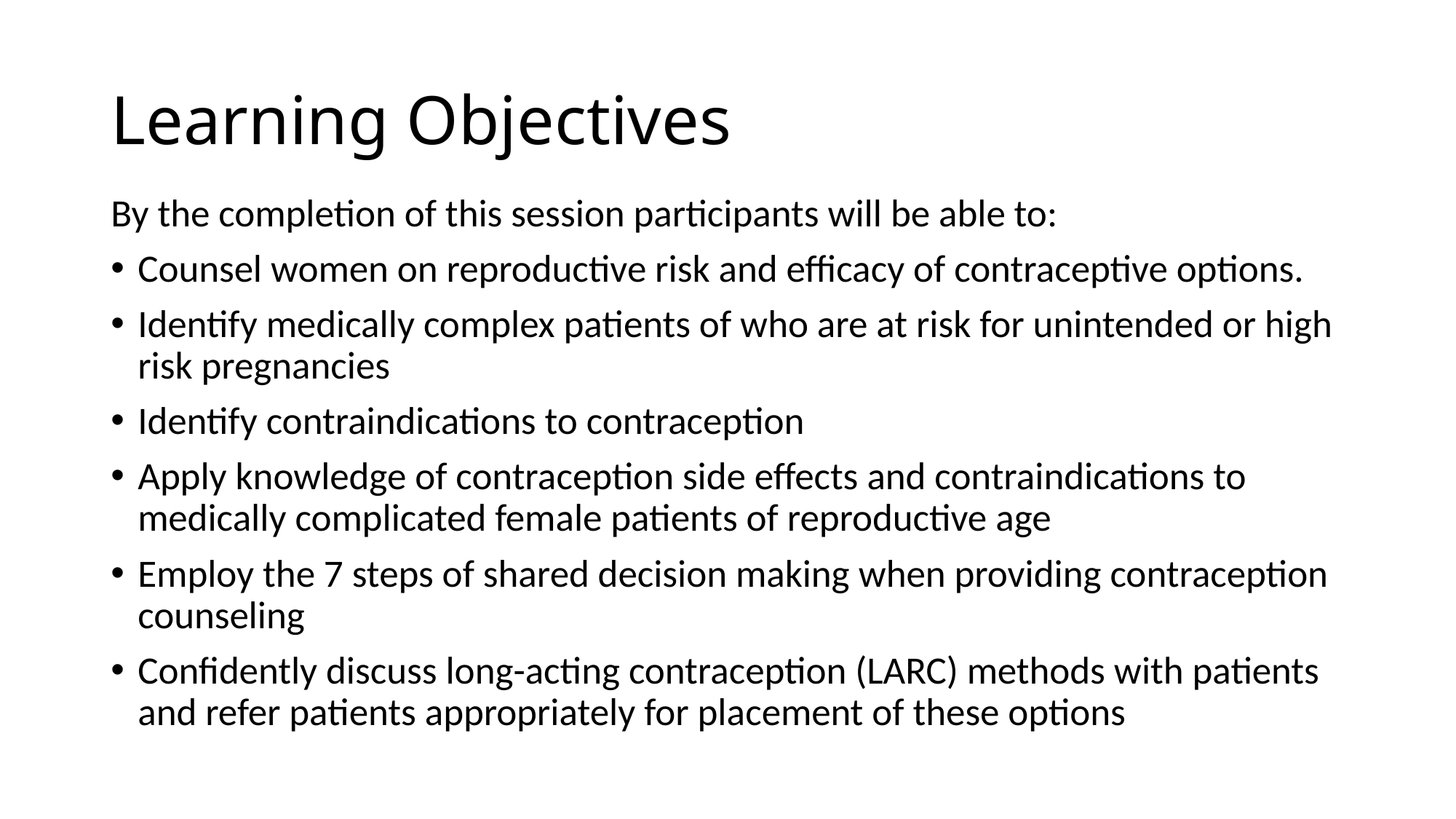

# Learning Objectives
By the completion of this session participants will be able to:
Counsel women on reproductive risk and efficacy of contraceptive options.
Identify medically complex patients of who are at risk for unintended or high risk pregnancies
Identify contraindications to contraception
Apply knowledge of contraception side effects and contraindications to medically complicated female patients of reproductive age
Employ the 7 steps of shared decision making when providing contraception counseling
Confidently discuss long-acting contraception (LARC) methods with patients and refer patients appropriately for placement of these options

## Slide 3
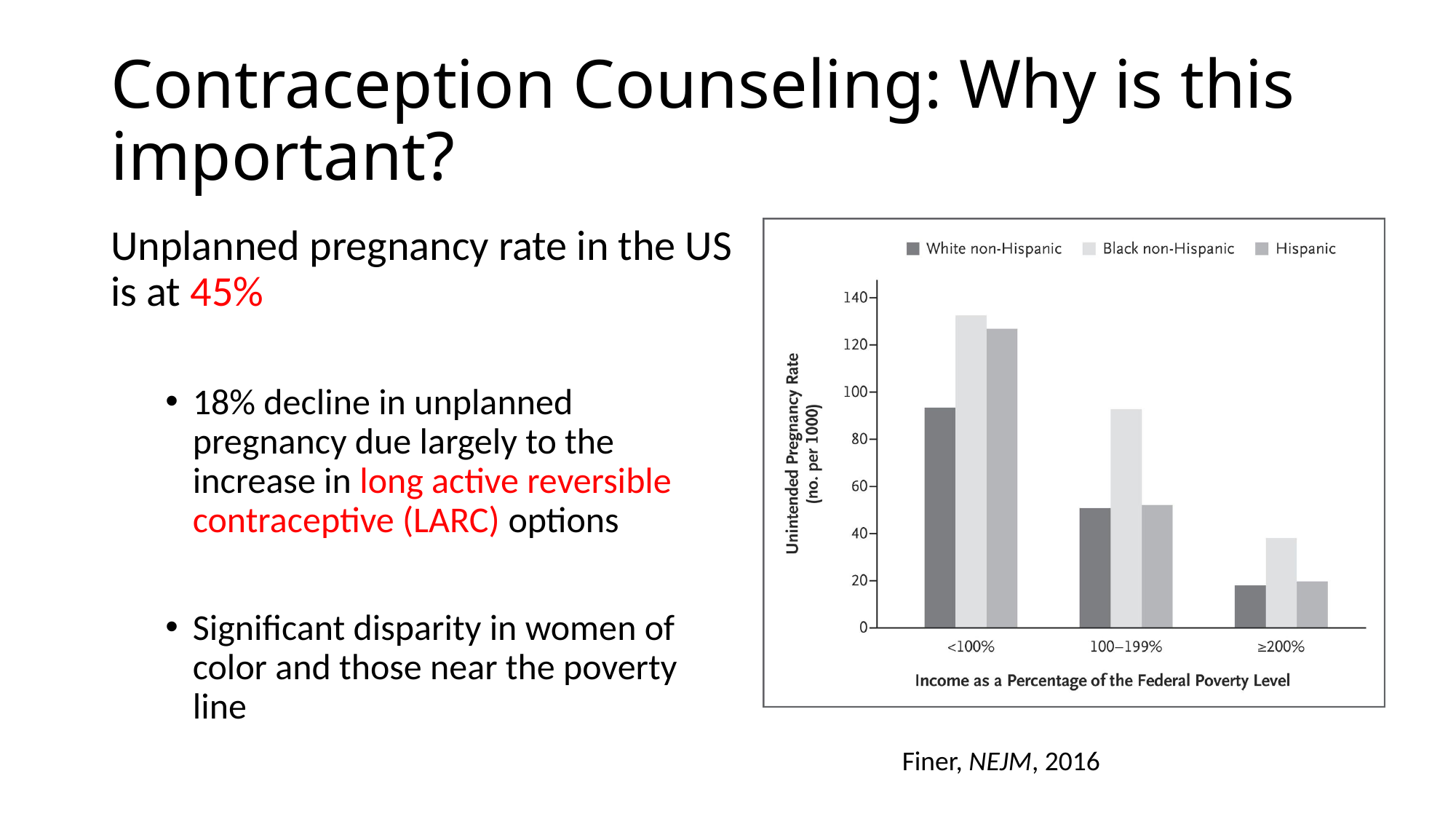

# Contraception Counseling: Why is this important?
Unplanned pregnancy rate in the US is at 45%
18% decline in unplanned pregnancy due largely to the increase in long active reversible contraceptive (LARC) options
Significant disparity in women of color and those near the poverty line
Finer, NEJM, 2016

## Slide 4
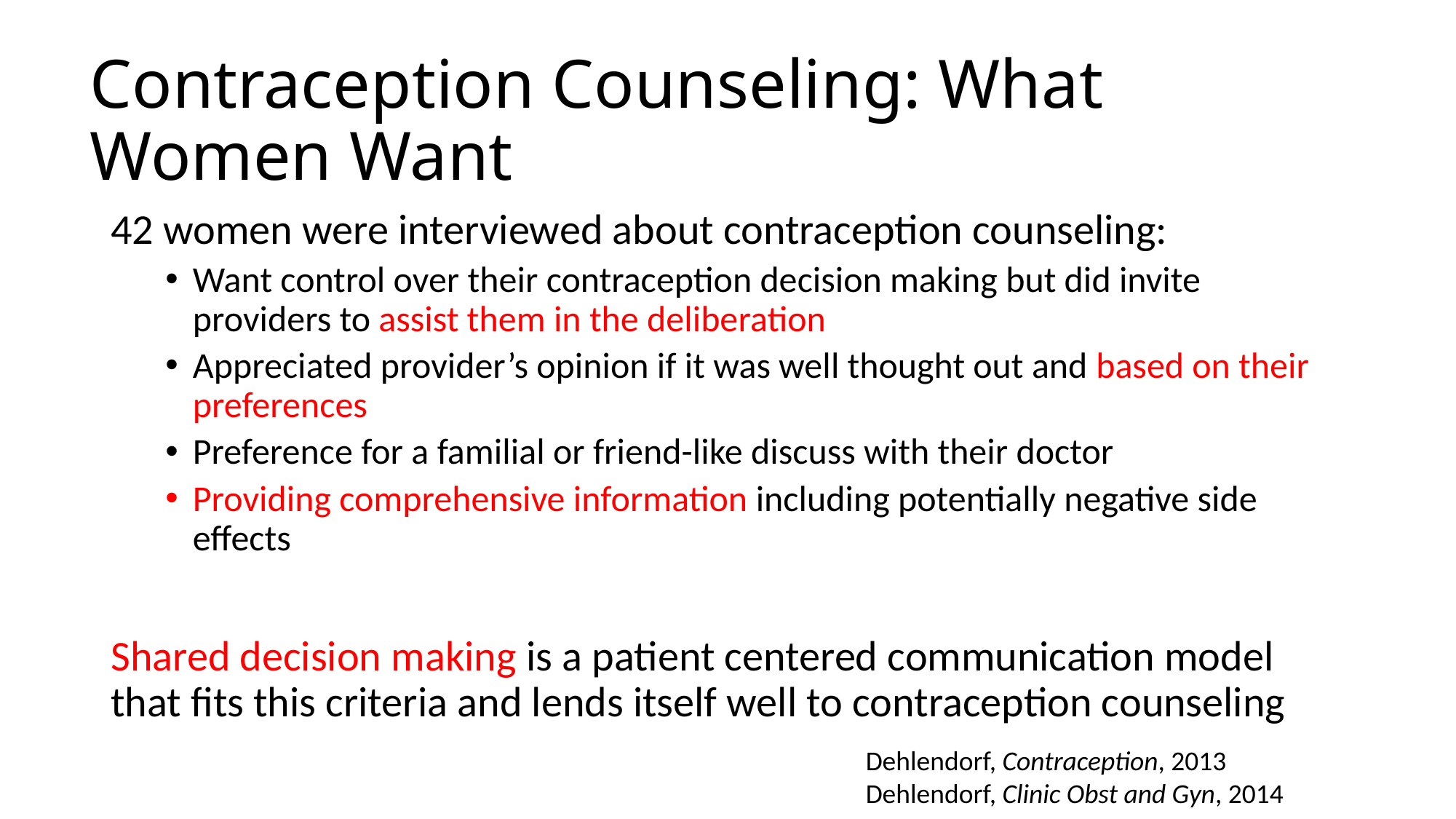

# Contraception Counseling: What Women Want
42 women were interviewed about contraception counseling:
Want control over their contraception decision making but did invite providers to assist them in the deliberation
Appreciated provider’s opinion if it was well thought out and based on their preferences
Preference for a familial or friend-like discuss with their doctor
Providing comprehensive information including potentially negative side effects
Shared decision making is a patient centered communication model that fits this criteria and lends itself well to contraception counseling
Dehlendorf, Contraception, 2013
Dehlendorf, Clinic Obst and Gyn, 2014

## Slide 5
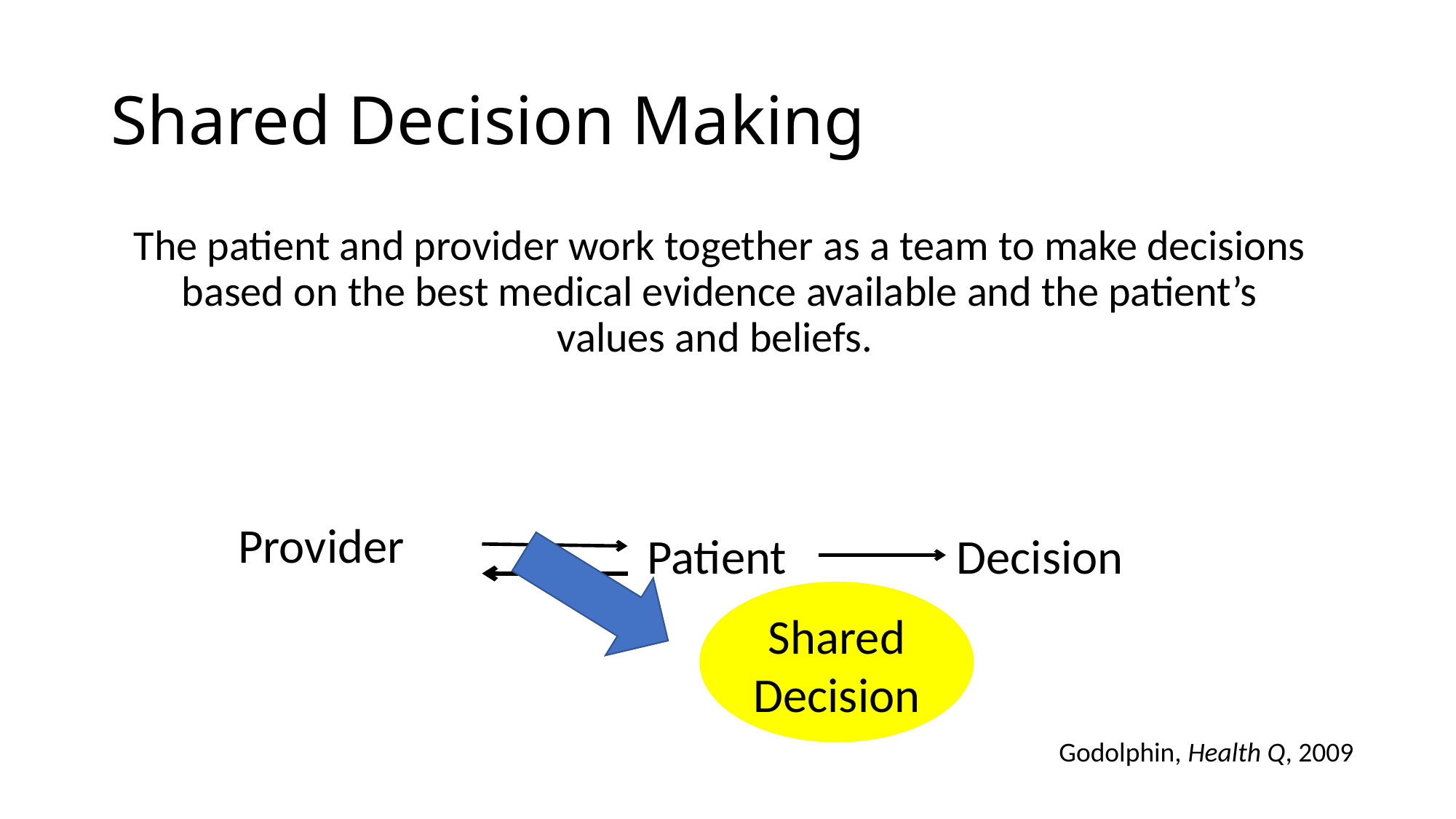

# Shared Decision Making
The patient and provider work together as a team to make decisions based on the best medical evidence available and the patient’s values and beliefs.
Provider
Patient
Decision
Shared
Decision
Godolphin, Health Q, 2009

## Slide 6
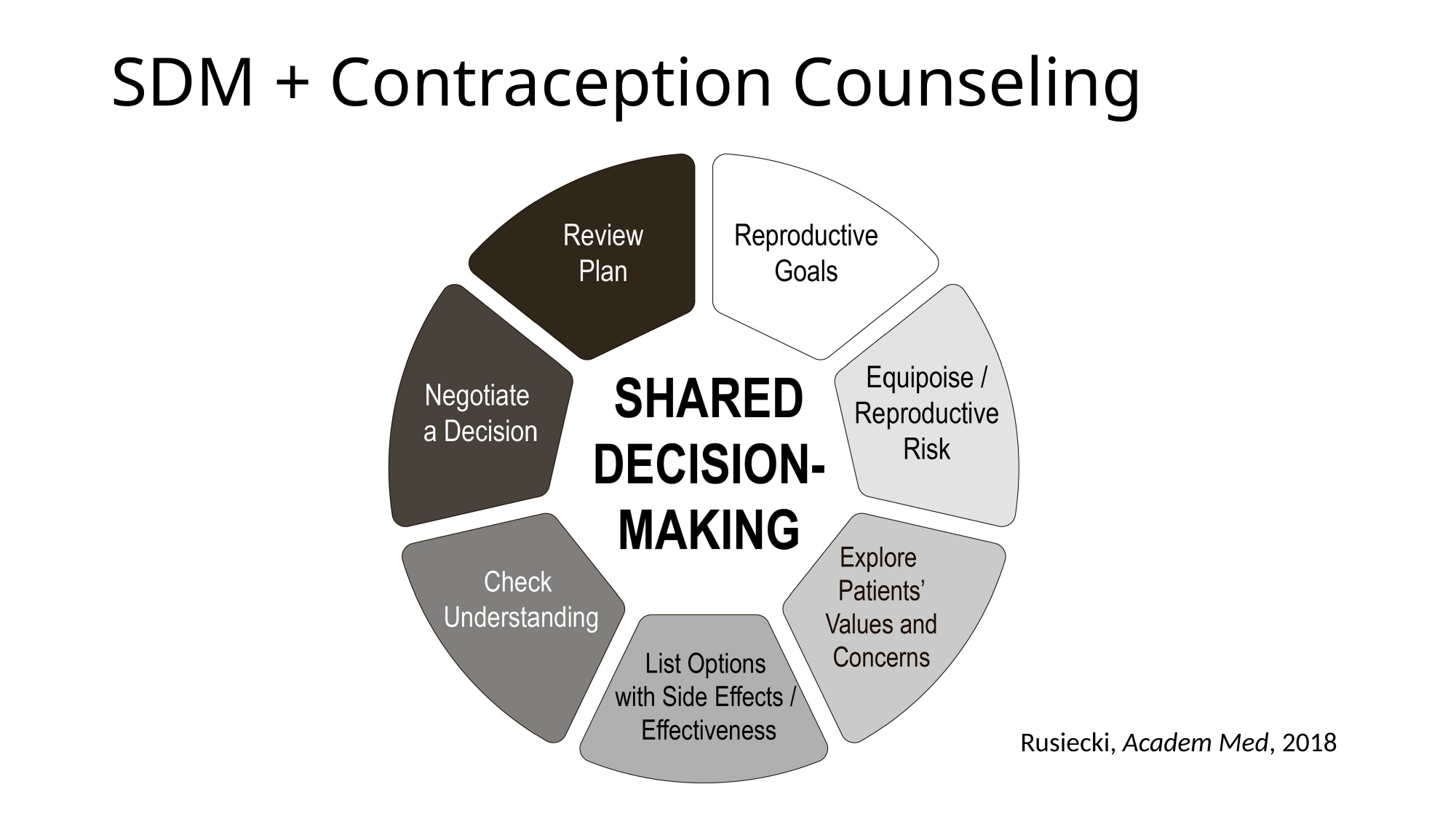

# SDM + Contraception Counseling
Rusiecki, Academ Med, 2018

## Slide 7
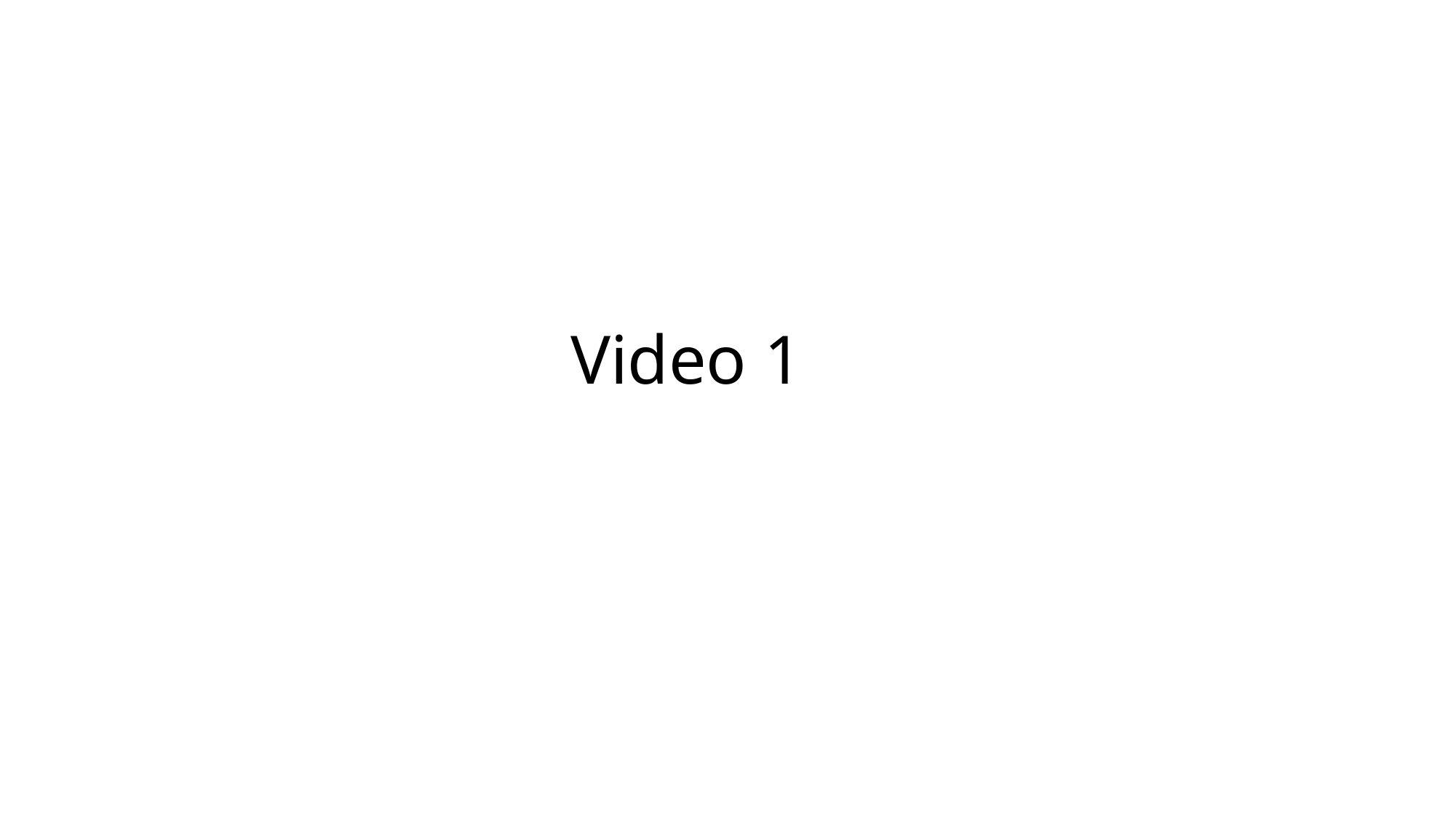

# Video 1

## Slide 8
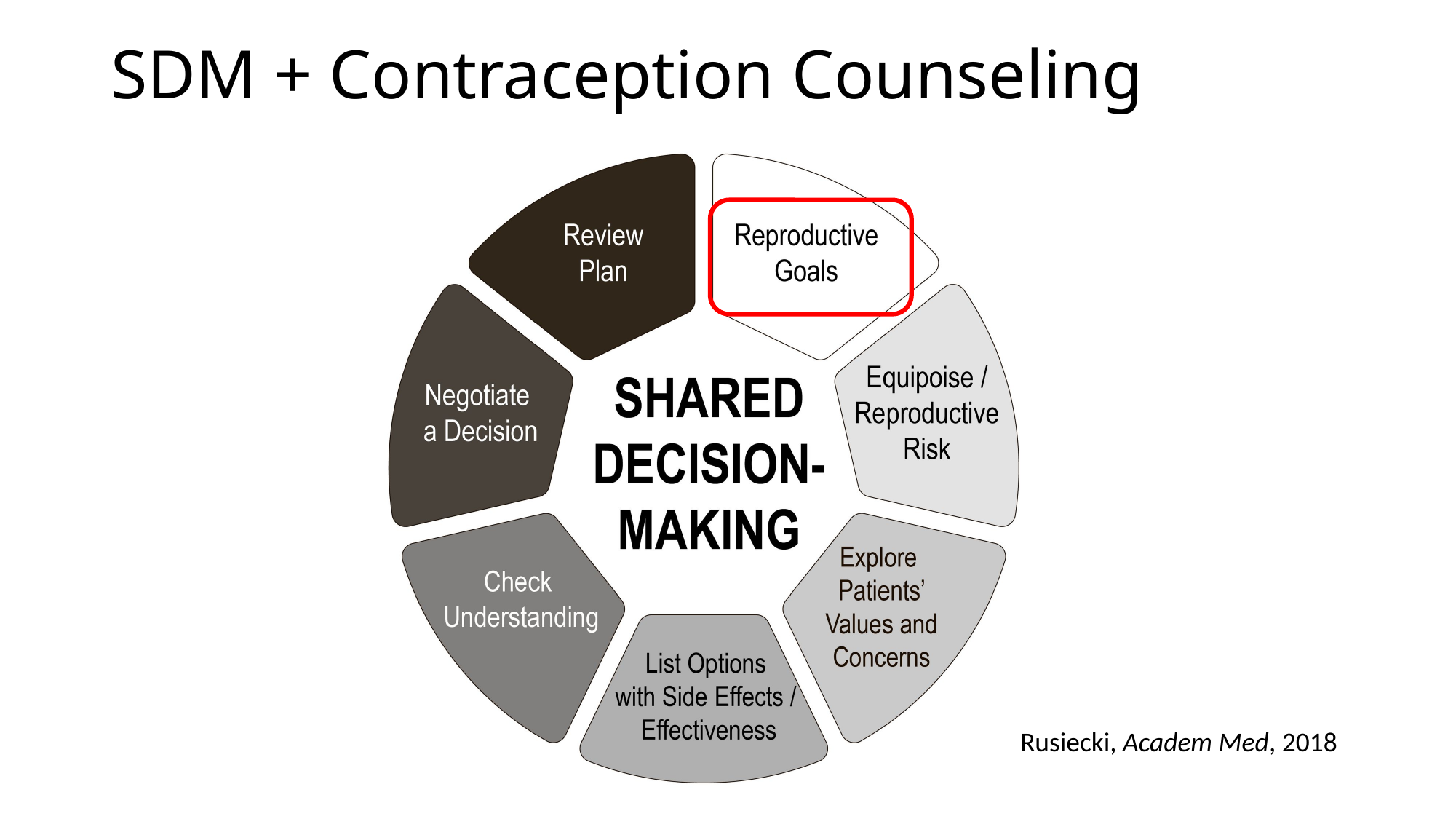

# SDM + Contraception Counseling
Rusiecki, Academ Med, 2018

## Slide 9
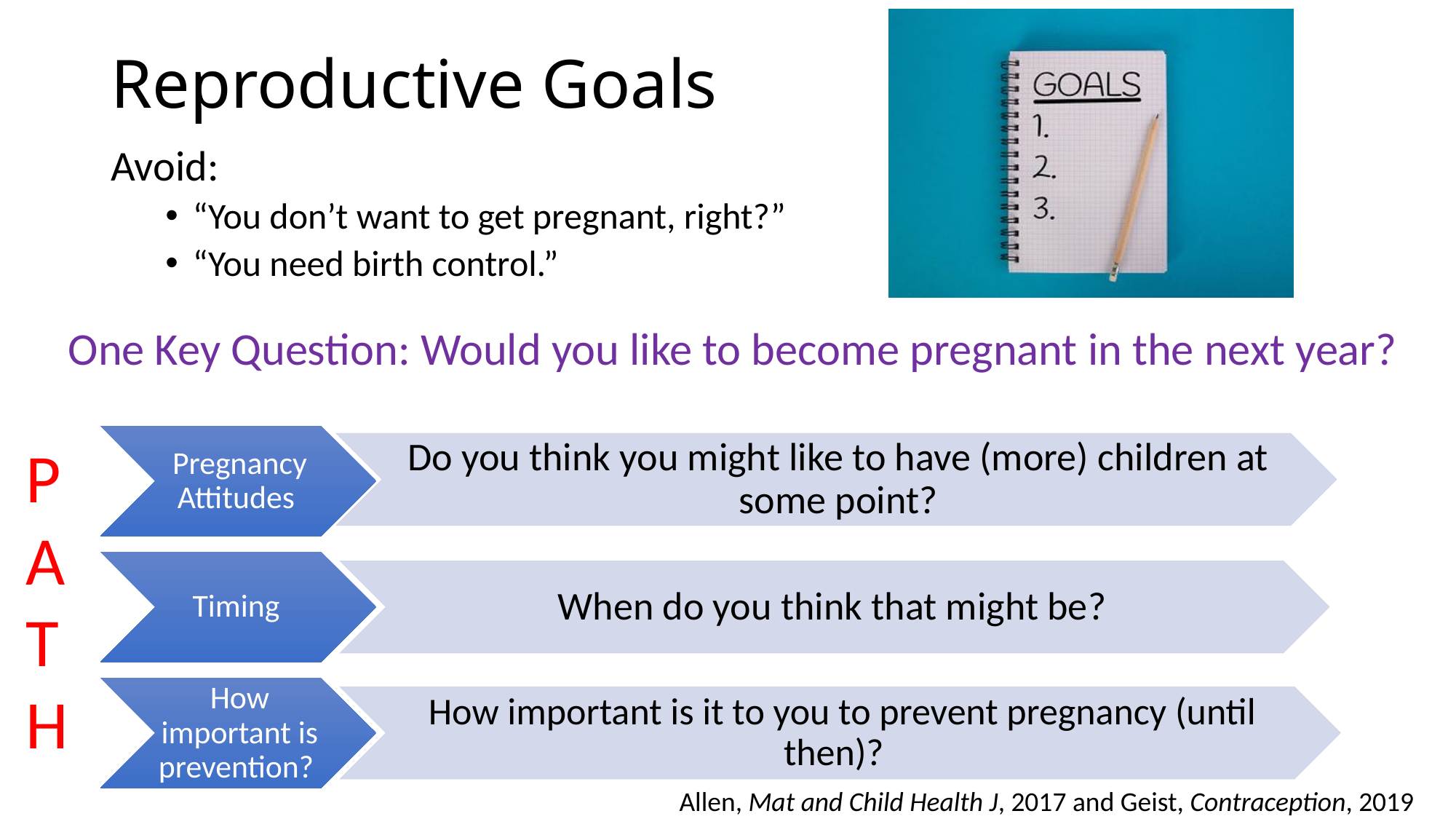

# Reproductive Goals
Avoid:
“You don’t want to get pregnant, right?”
“You need birth control.”
One Key Question: Would you like to become pregnant in the next year?
P
A
T
H
Allen, Mat and Child Health J, 2017 and Geist, Contraception, 2019

## Slide 10
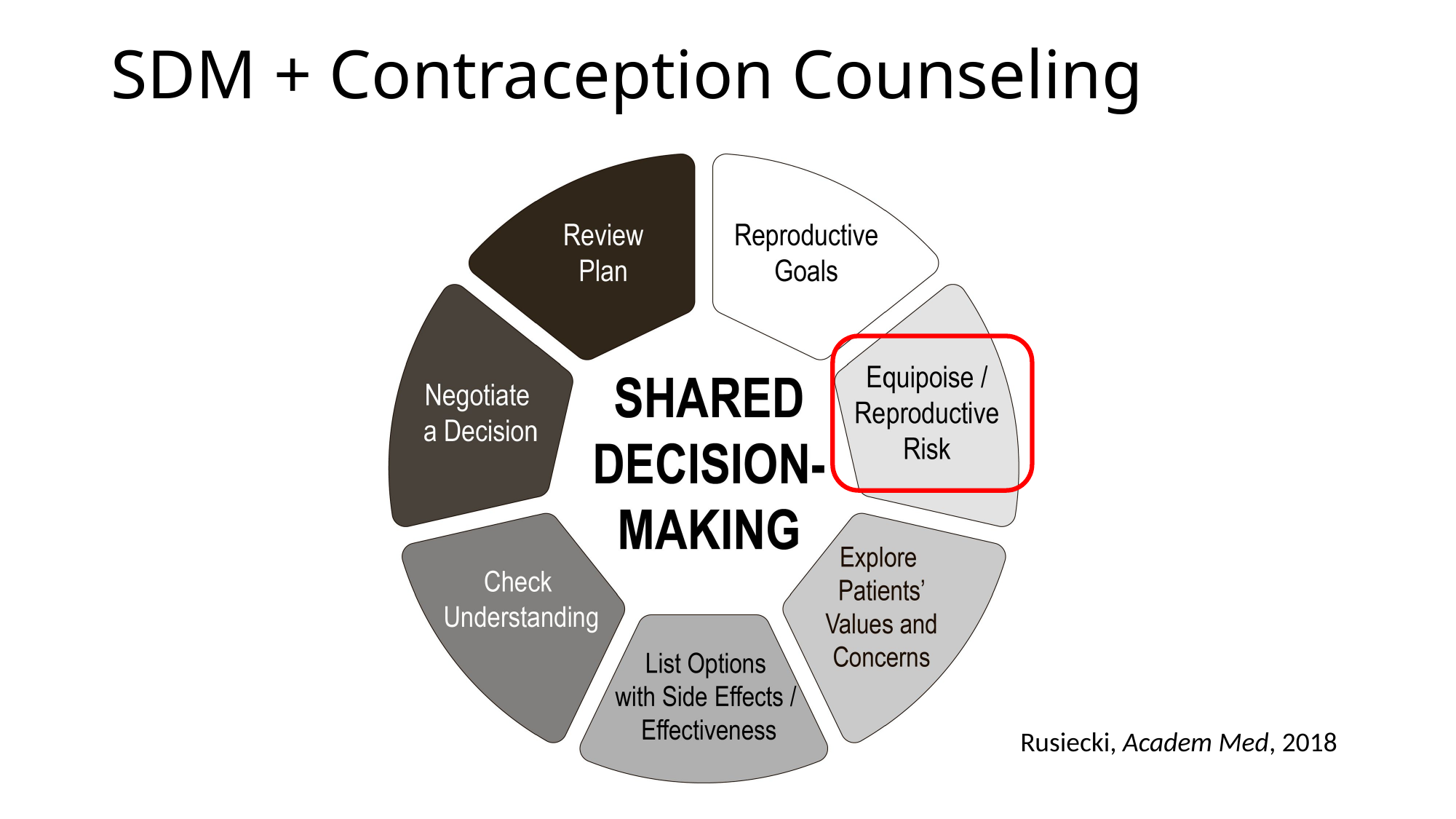

# SDM + Contraception Counseling
Rusiecki, Academ Med, 2018

## Slide 11
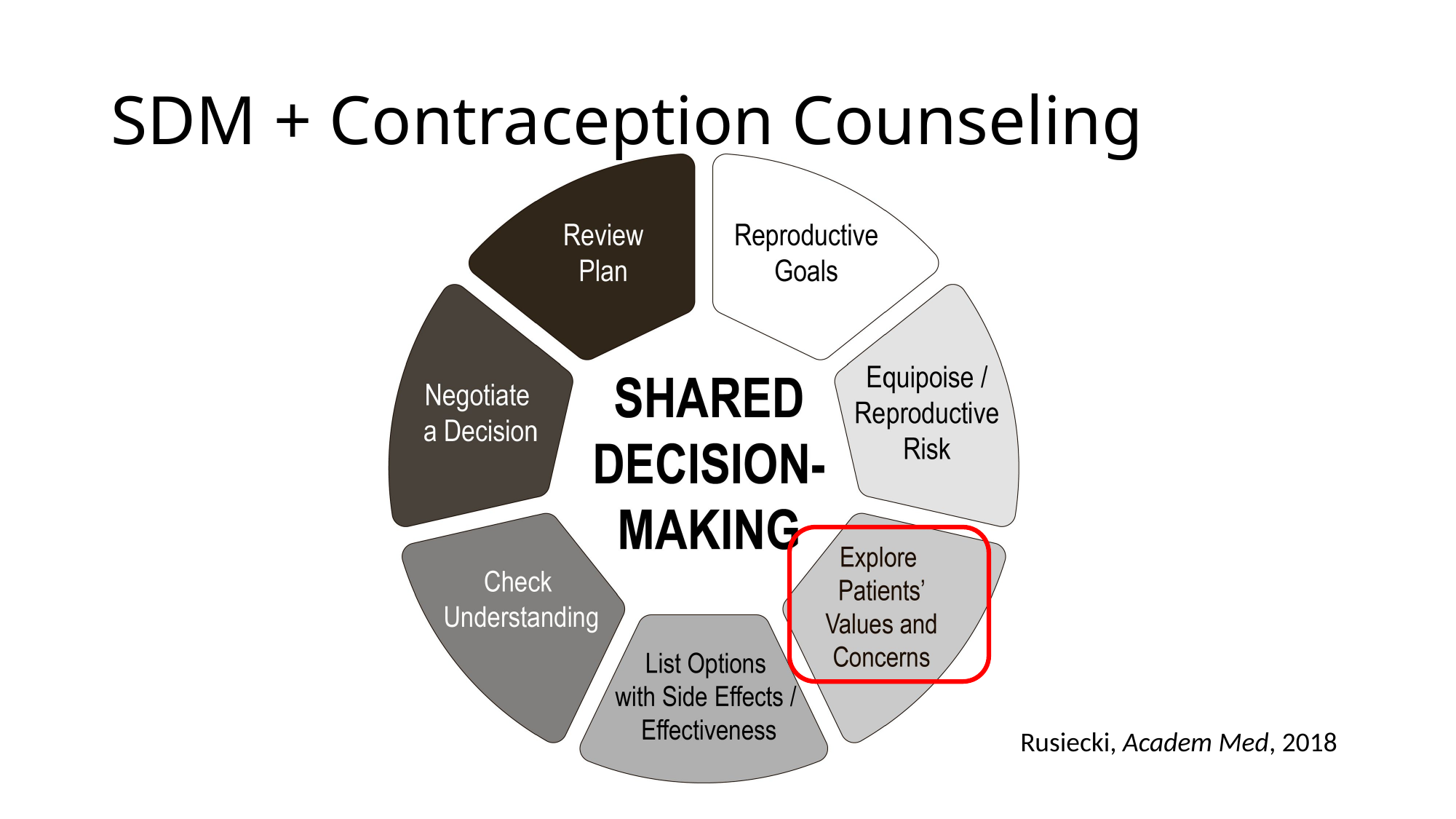

# SDM + Contraception Counseling
Rusiecki, Academ Med, 2018

## Slide 12
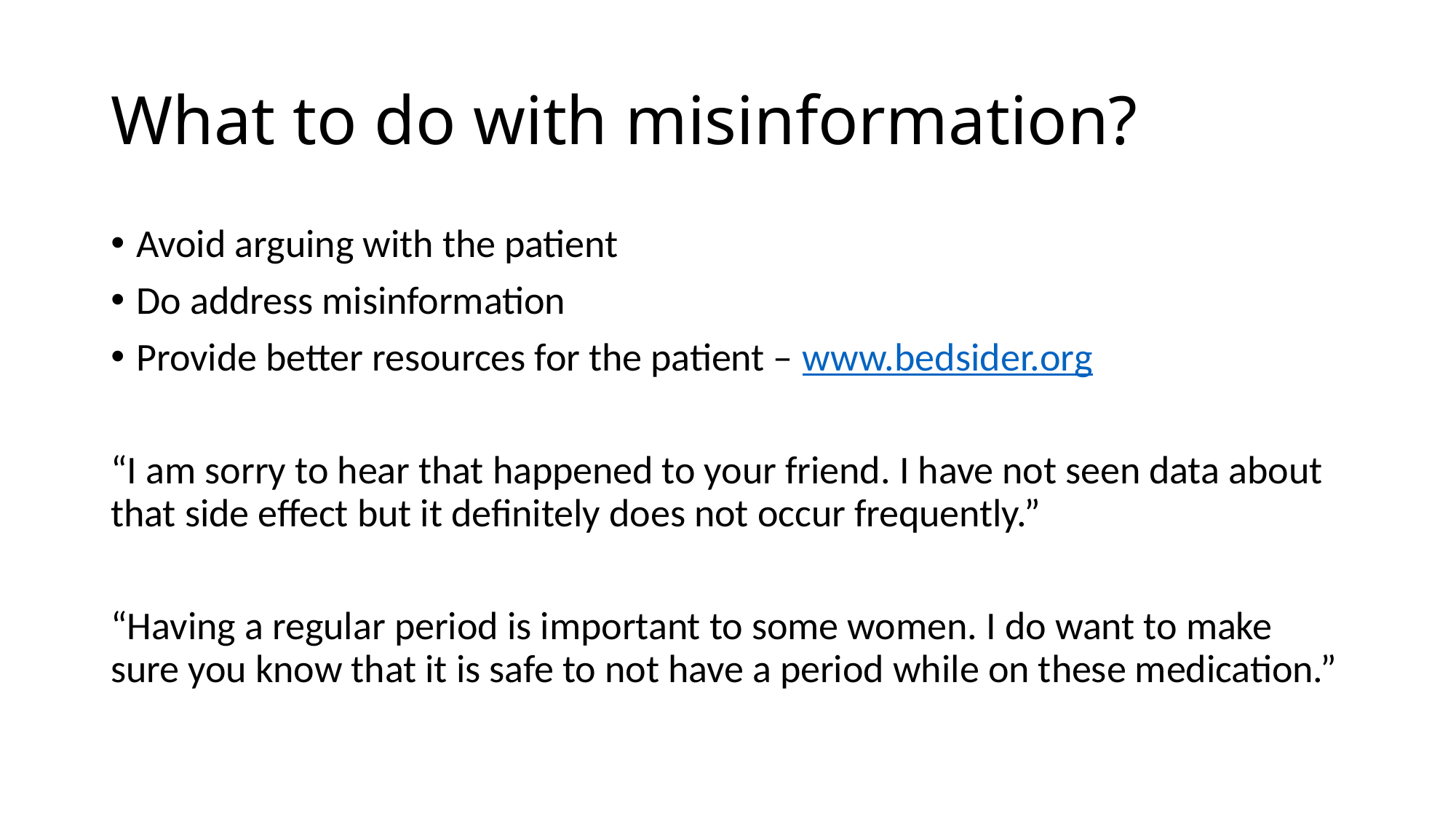

# What to do with misinformation?
Avoid arguing with the patient
Do address misinformation
Provide better resources for the patient – www.bedsider.org
“I am sorry to hear that happened to your friend. I have not seen data about that side effect but it definitely does not occur frequently.”
“Having a regular period is important to some women. I do want to make sure you know that it is safe to not have a period while on these medication.”

## Slide 13
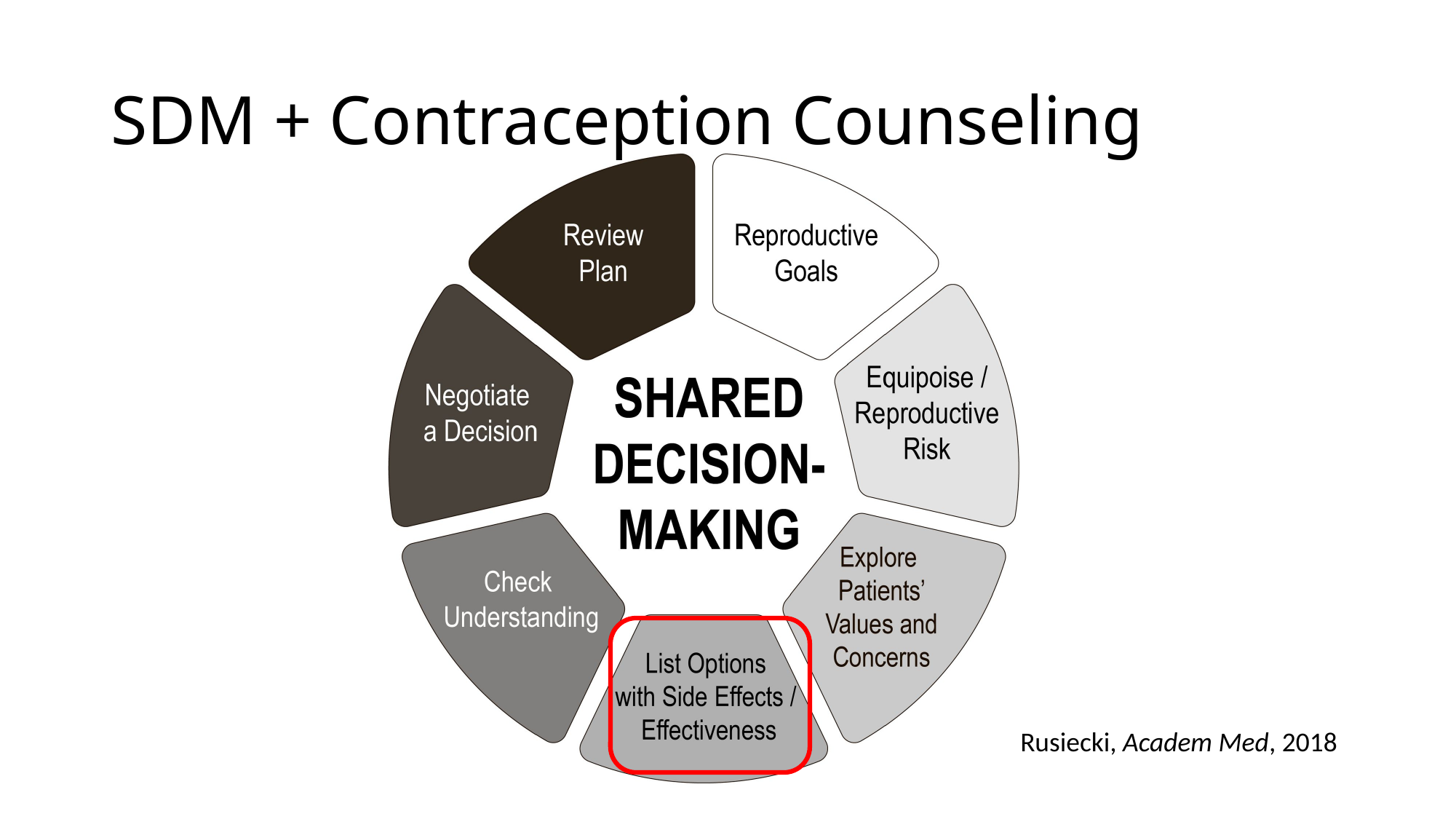

# SDM + Contraception Counseling
Rusiecki, Academ Med, 2018

## Slide 14
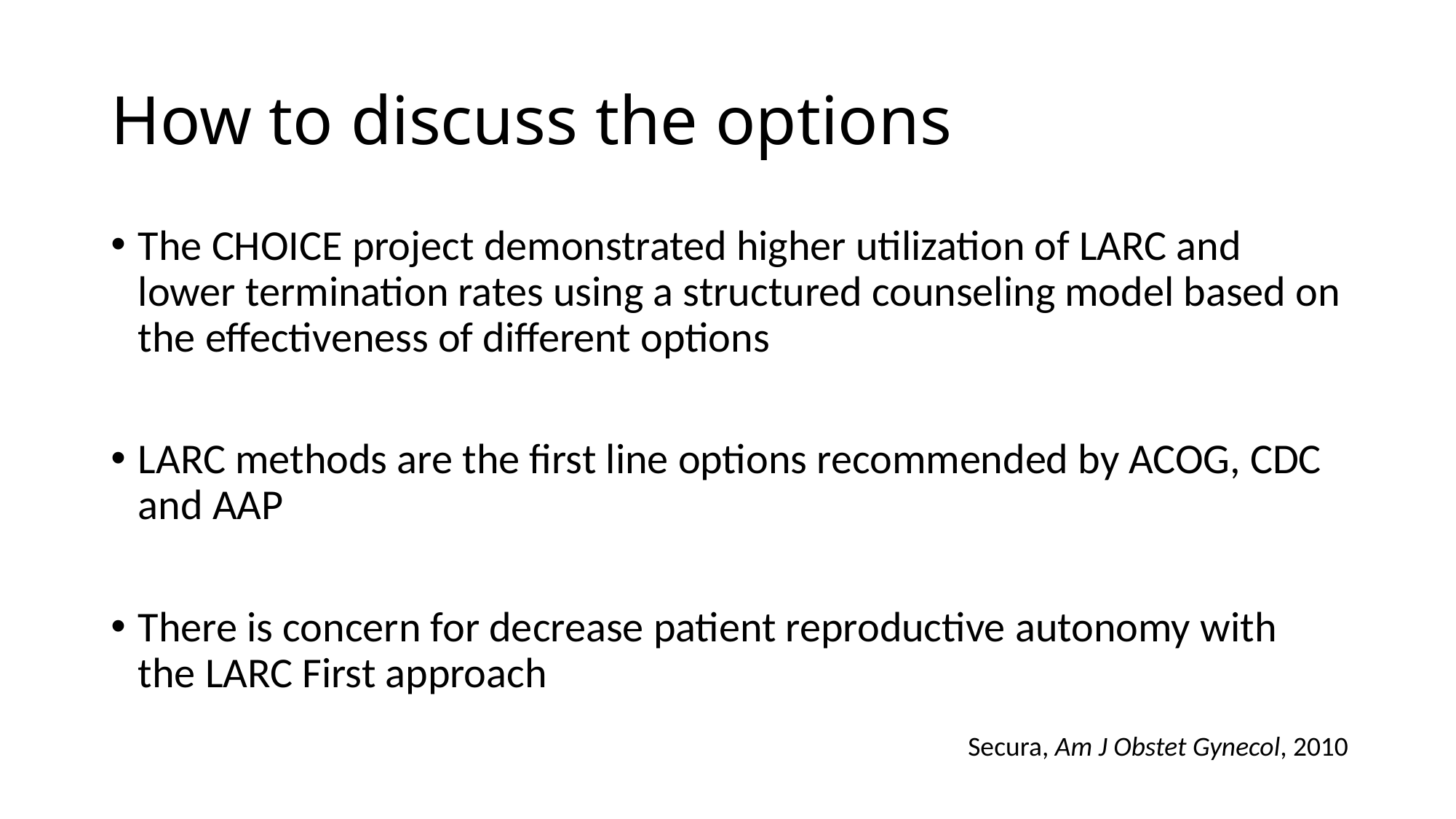

# How to discuss the options
The CHOICE project demonstrated higher utilization of LARC and lower termination rates using a structured counseling model based on the effectiveness of different options
LARC methods are the first line options recommended by ACOG, CDC and AAP
There is concern for decrease patient reproductive autonomy with the LARC First approach
Secura, Am J Obstet Gynecol, 2010

## Slide 15
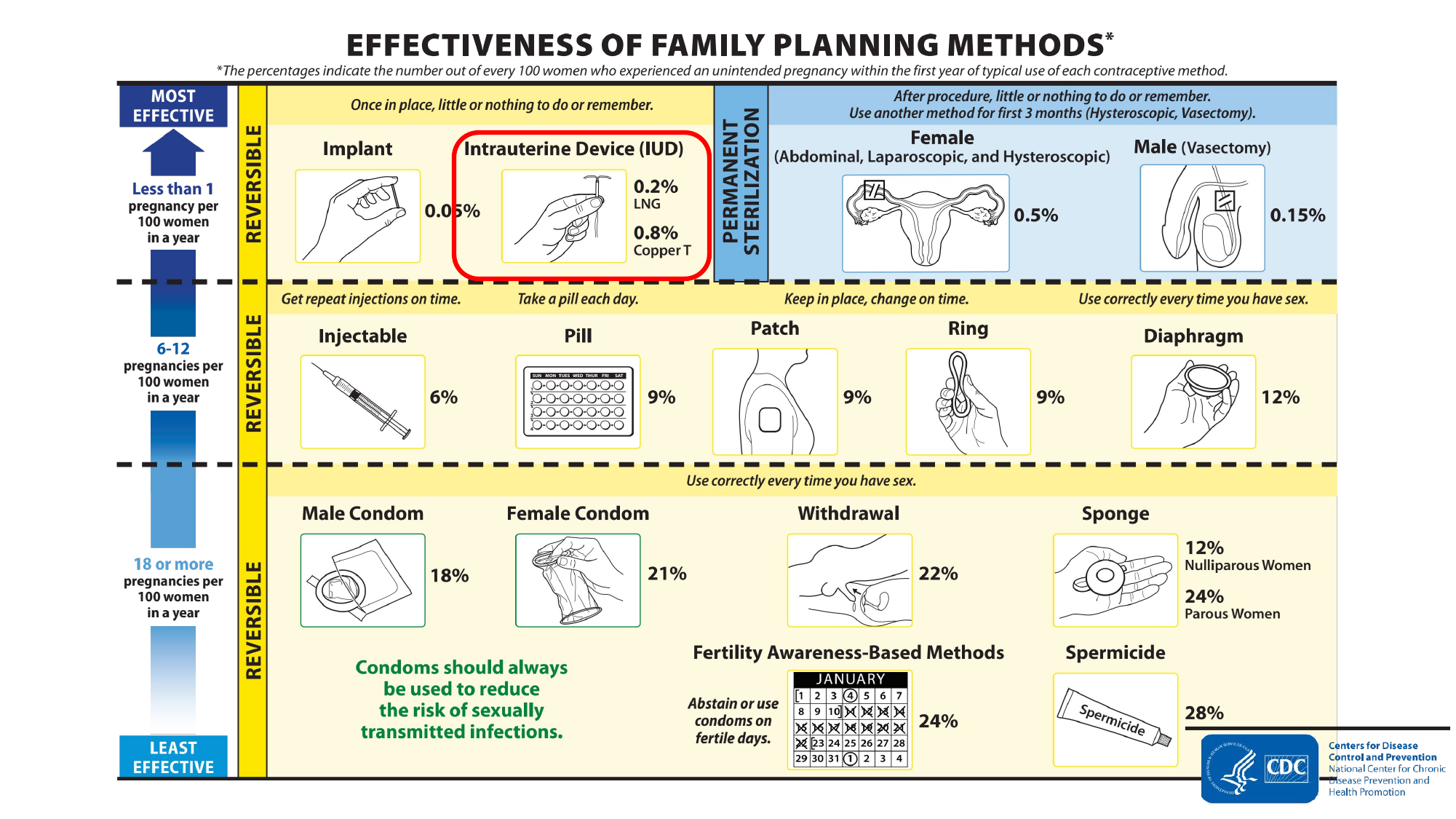

## Slide 16
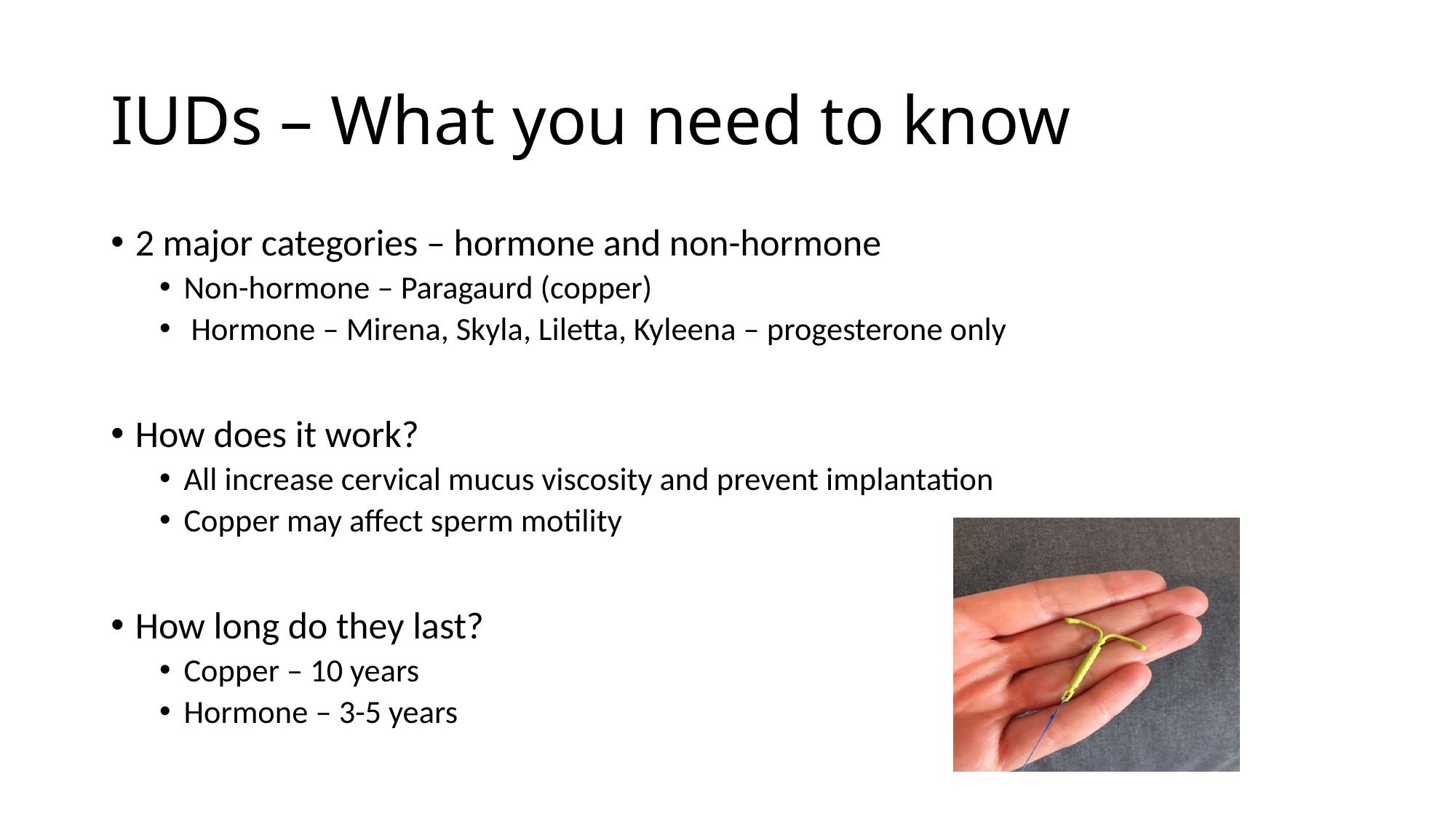

# IUDs – What you need to know
2 major categories – hormone and non-hormone
Non-hormone – Paragaurd (copper)
 Hormone – Mirena, Skyla, Liletta, Kyleena – progesterone only
How does it work?
All increase cervical mucus viscosity and prevent implantation
Copper may affect sperm motility
How long do they last?
Copper – 10 years
Hormone – 3-5 years

## Slide 17
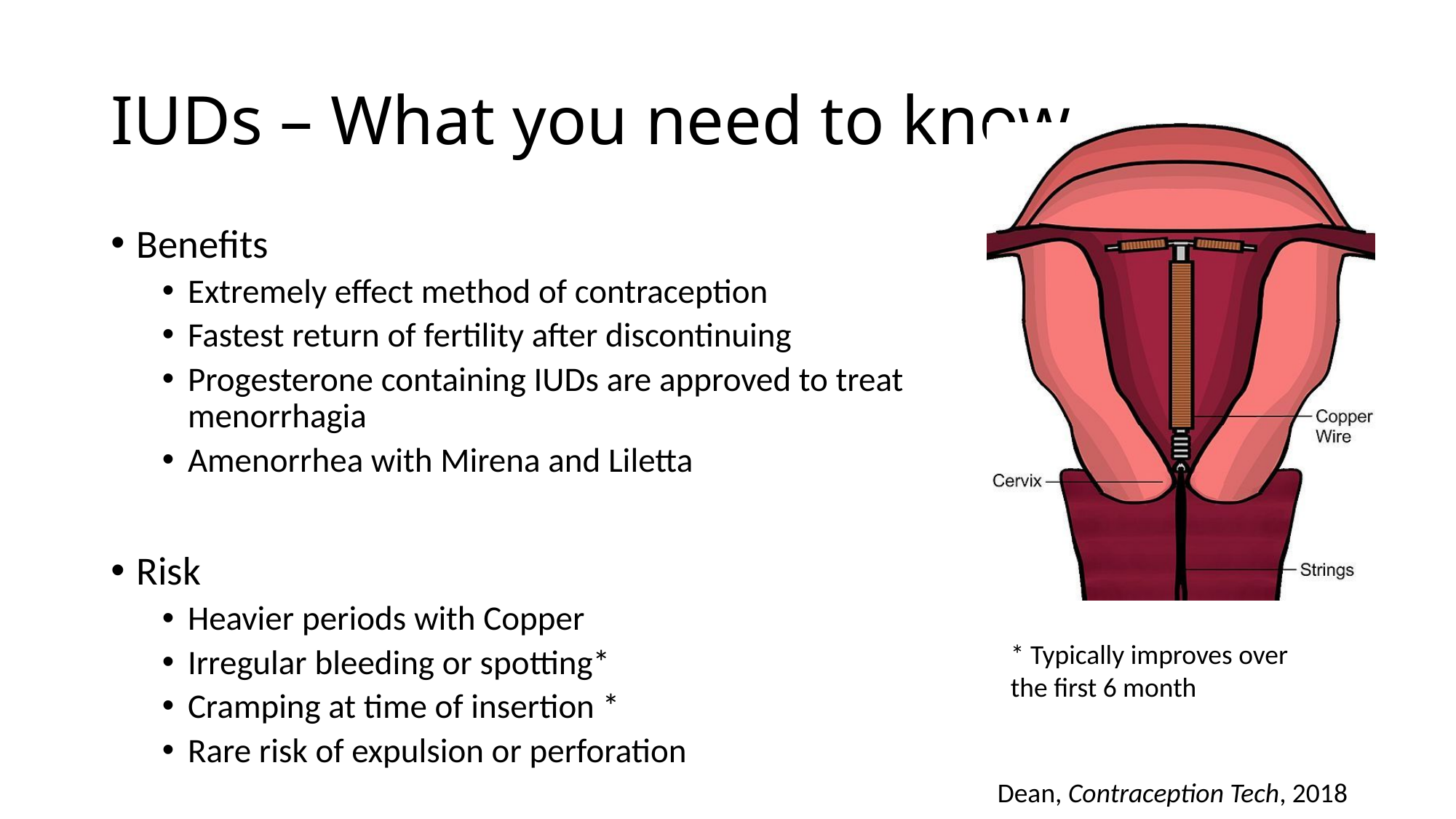

# IUDs – What you need to know
Benefits
Extremely effect method of contraception
Fastest return of fertility after discontinuing
Progesterone containing IUDs are approved to treat menorrhagia
Amenorrhea with Mirena and Liletta
Risk
Heavier periods with Copper
Irregular bleeding or spotting*
Cramping at time of insertion *
Rare risk of expulsion or perforation
* Typically improves over the first 6 month
Dean, Contraception Tech, 2018

## Slide 18
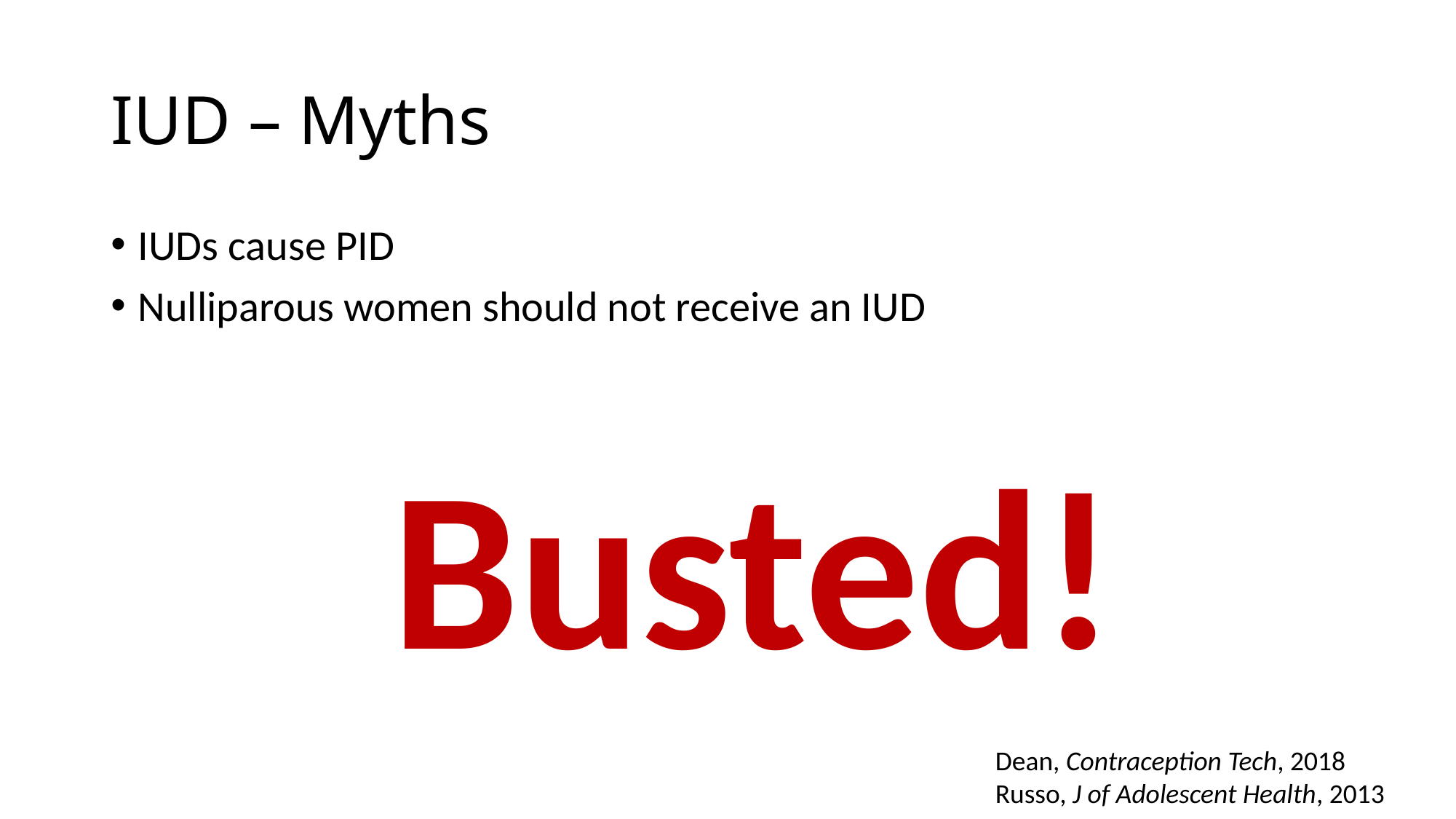

# IUD – Myths
IUDs cause PID
Nulliparous women should not receive an IUD
Busted!
Dean, Contraception Tech, 2018
Russo, J of Adolescent Health, 2013

## Slide 19
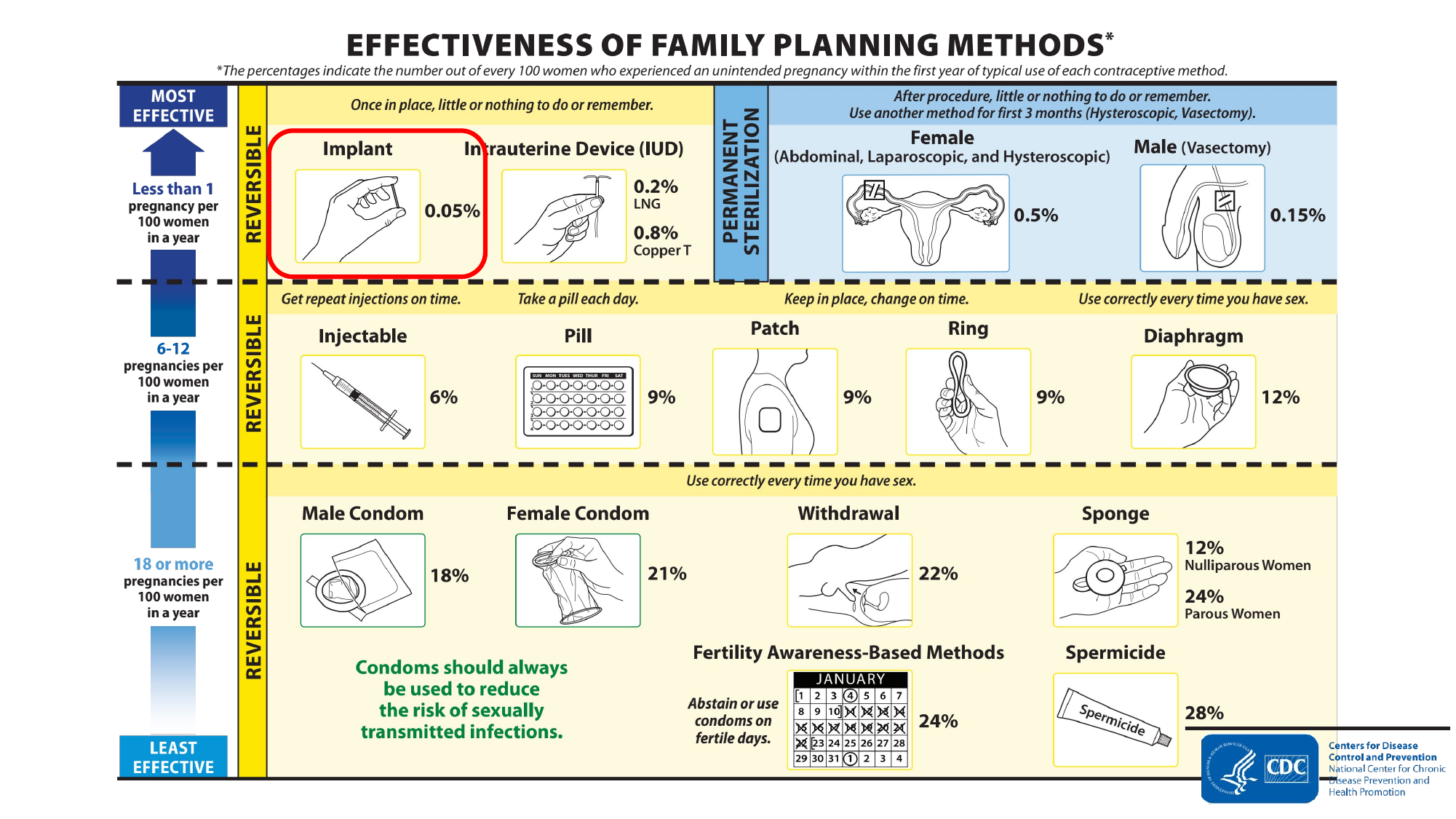

## Slide 20
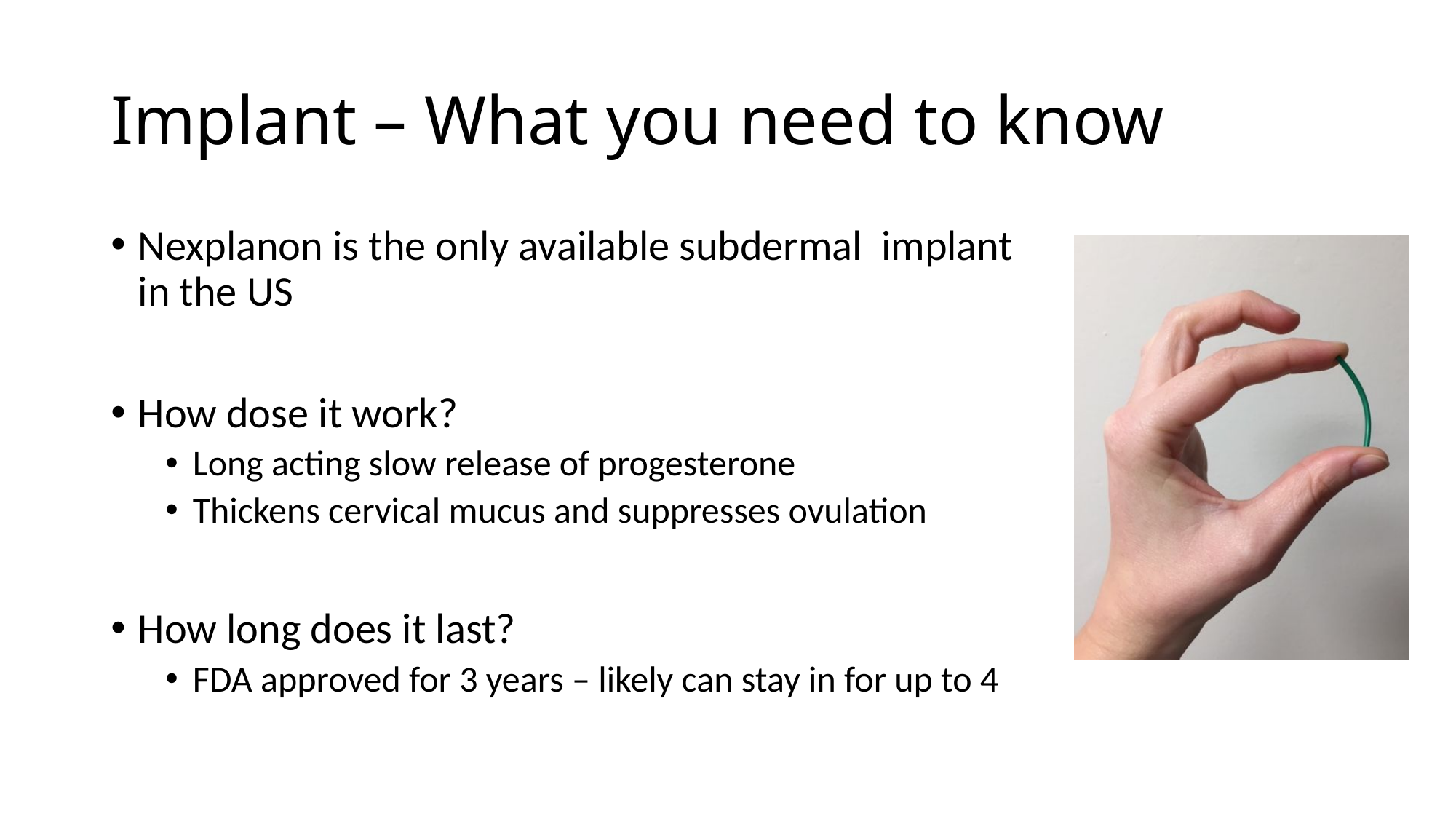

# Implant – What you need to know
Nexplanon is the only available subdermal implant in the US
How dose it work?
Long acting slow release of progesterone
Thickens cervical mucus and suppresses ovulation
How long does it last?
FDA approved for 3 years – likely can stay in for up to 4

## Slide 21
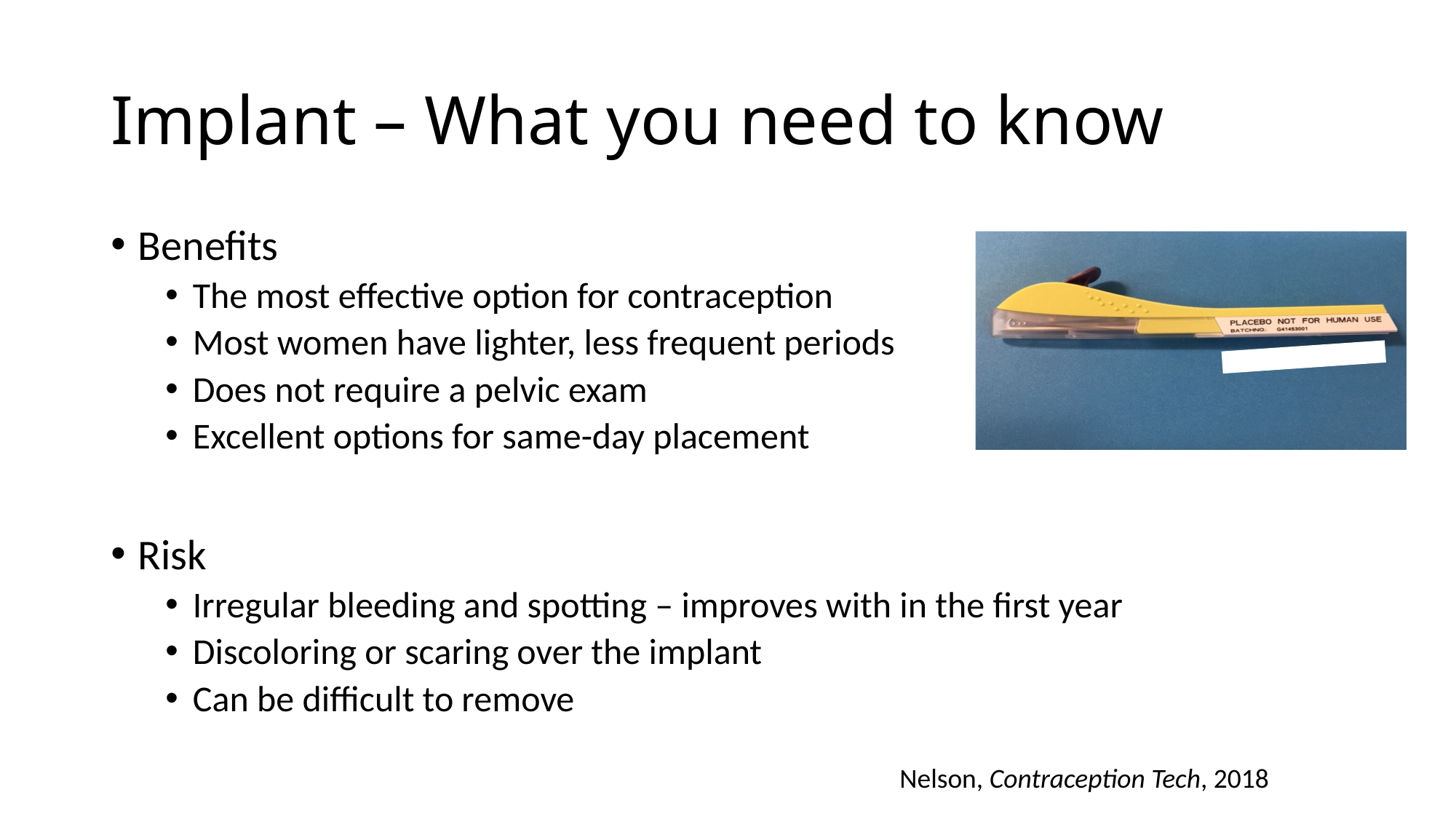

# Implant – What you need to know
Benefits
The most effective option for contraception
Most women have lighter, less frequent periods
Does not require a pelvic exam
Excellent options for same-day placement
Risk
Irregular bleeding and spotting – improves with in the first year
Discoloring or scaring over the implant
Can be difficult to remove
Nelson, Contraception Tech, 2018

## Slide 22
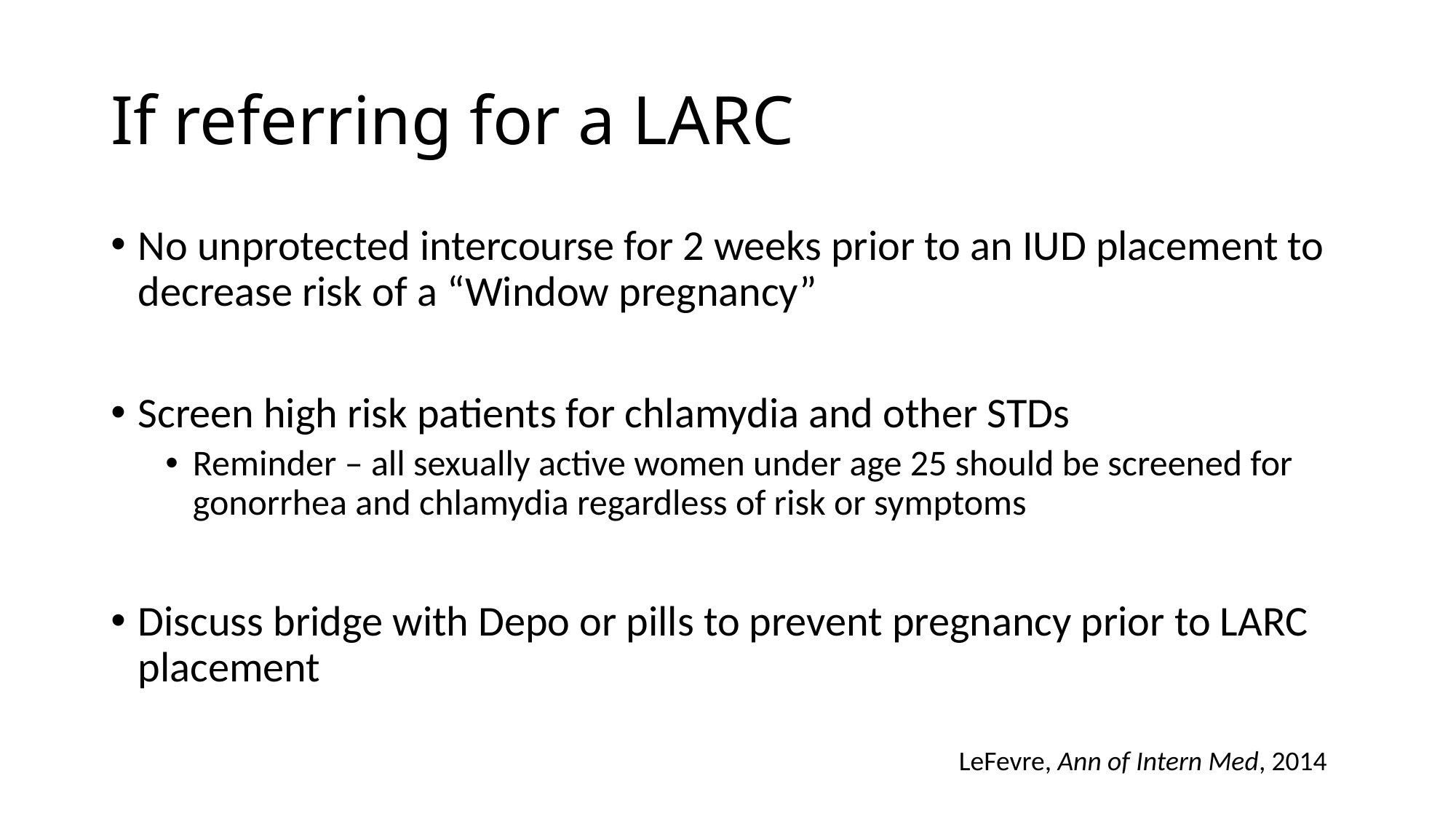

# If referring for a LARC
No unprotected intercourse for 2 weeks prior to an IUD placement to decrease risk of a “Window pregnancy”
Screen high risk patients for chlamydia and other STDs
Reminder – all sexually active women under age 25 should be screened for gonorrhea and chlamydia regardless of risk or symptoms
Discuss bridge with Depo or pills to prevent pregnancy prior to LARC placement
LeFevre, Ann of Intern Med, 2014

## Slide 23
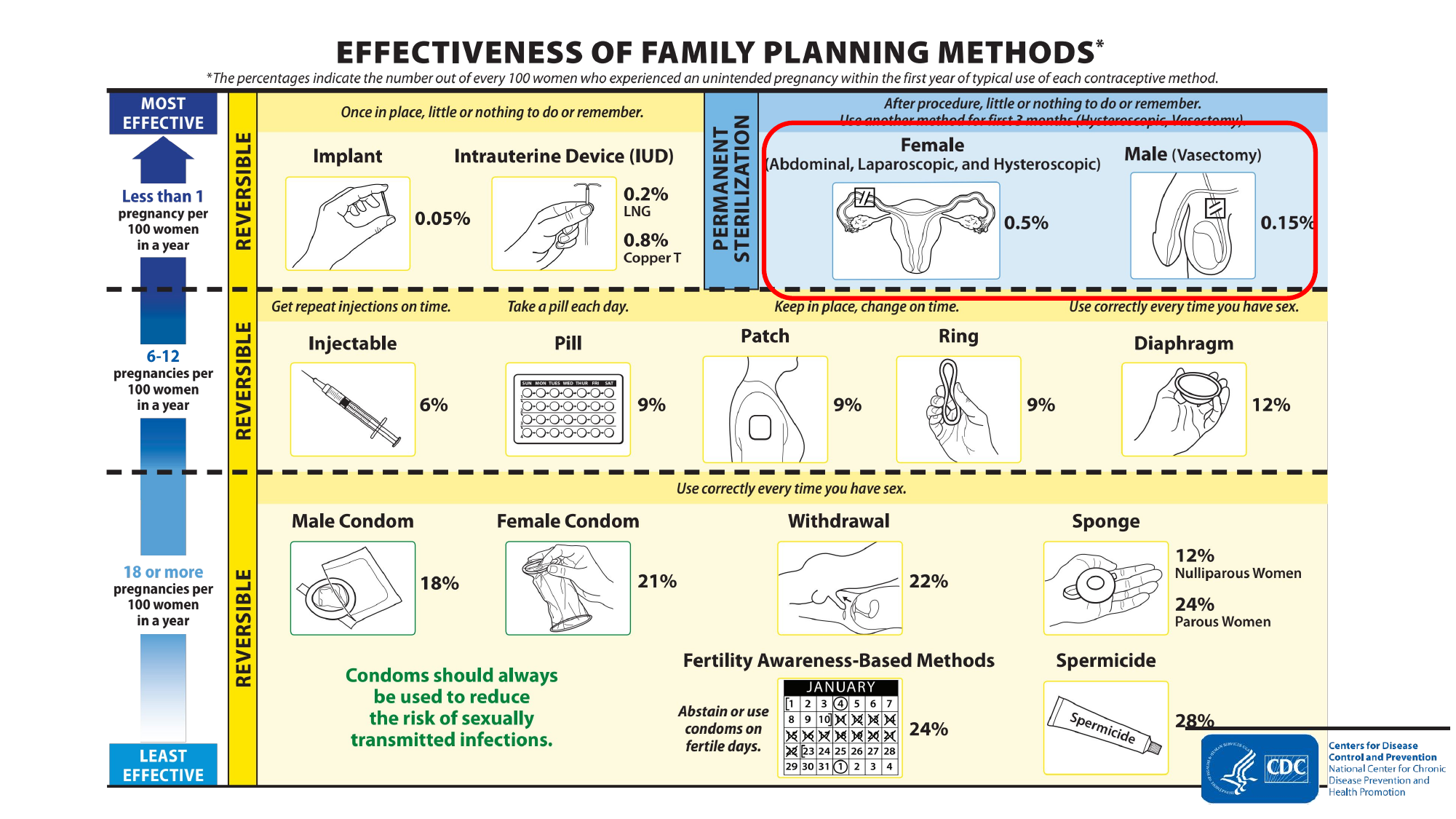

## Slide 24
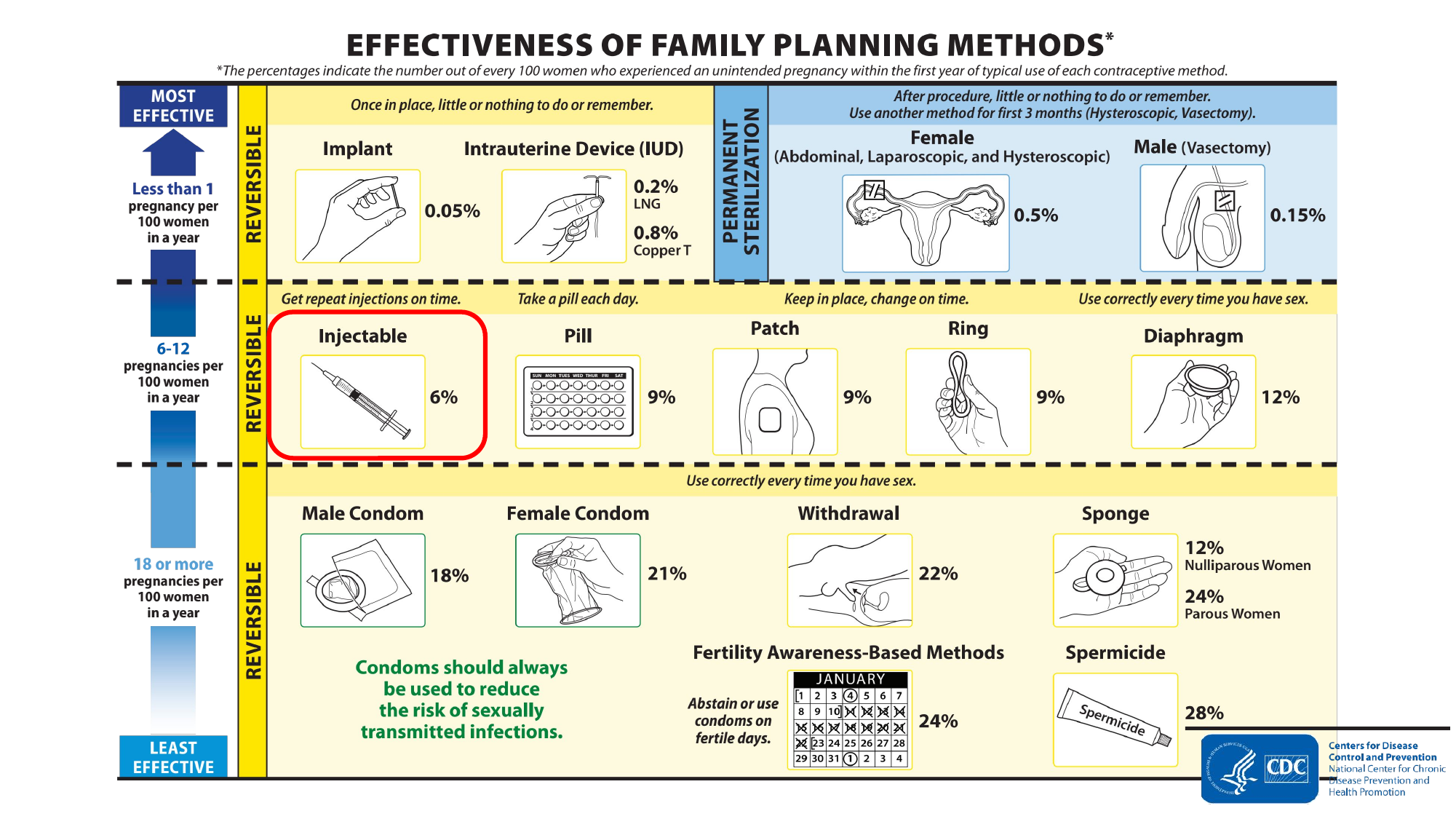

## Slide 25
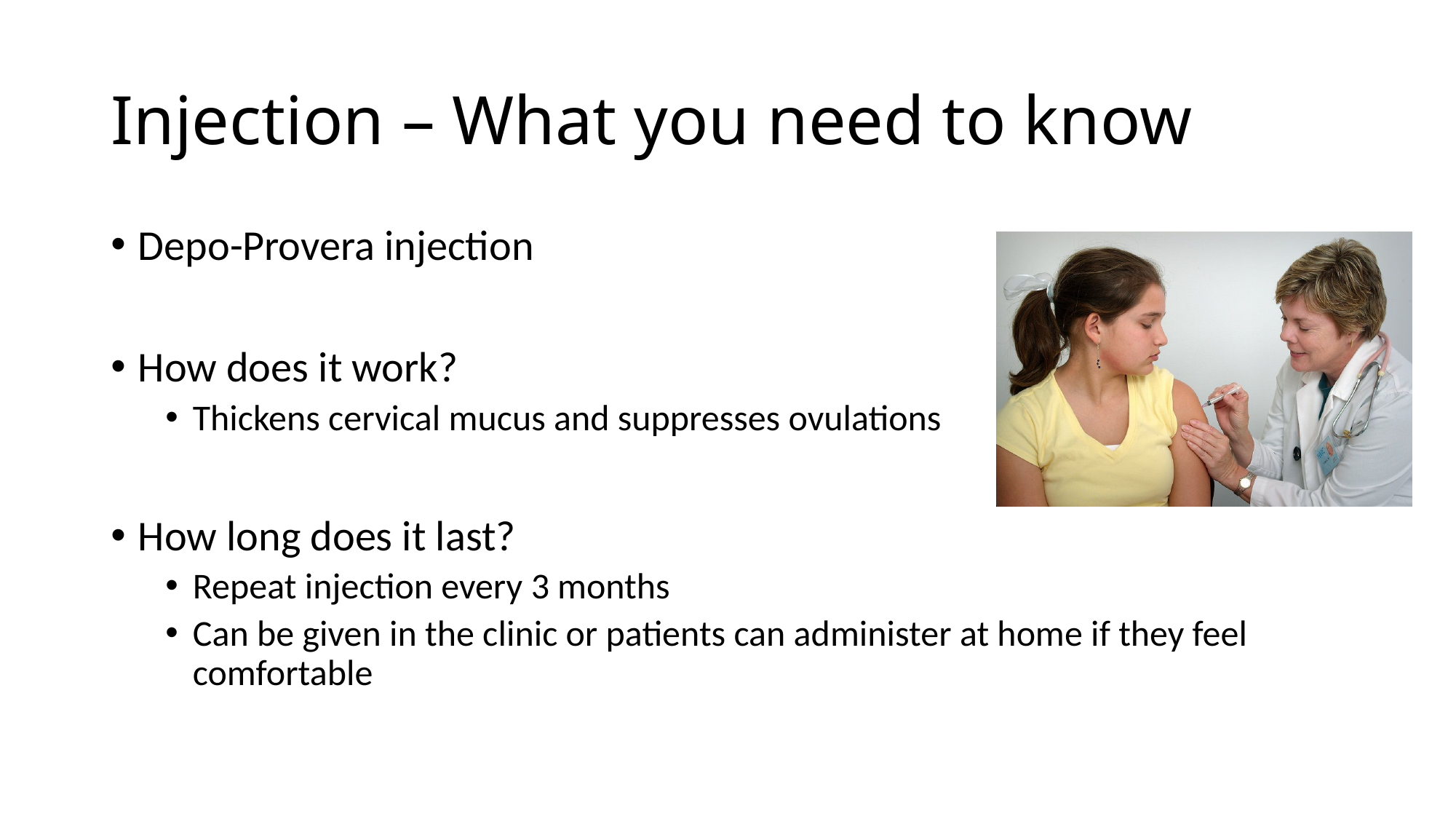

# Injection – What you need to know
Depo-Provera injection
How does it work?
Thickens cervical mucus and suppresses ovulations
How long does it last?
Repeat injection every 3 months
Can be given in the clinic or patients can administer at home if they feel comfortable

## Slide 26
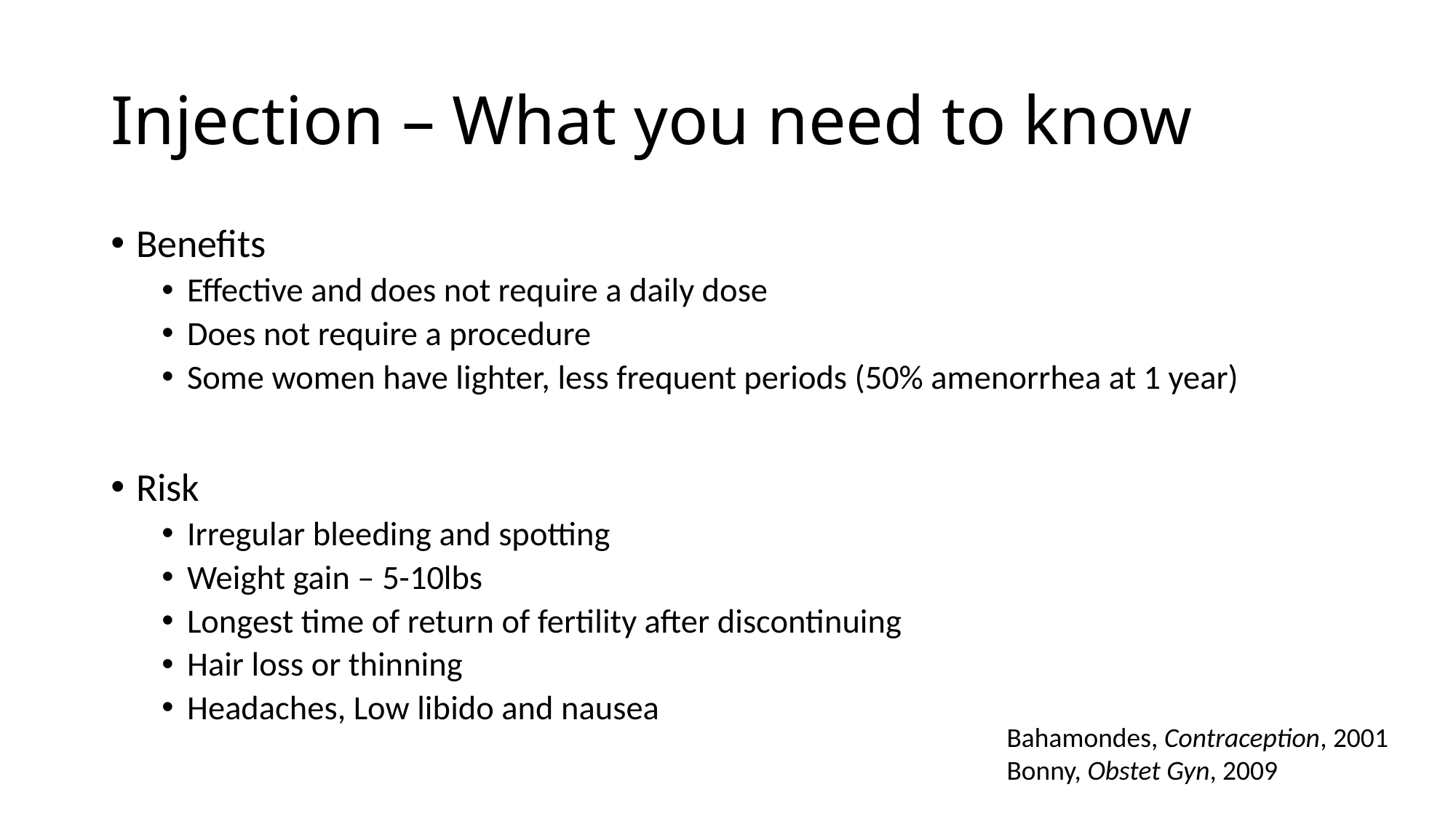

# Injection – What you need to know
Benefits
Effective and does not require a daily dose
Does not require a procedure
Some women have lighter, less frequent periods (50% amenorrhea at 1 year)
Risk
Irregular bleeding and spotting
Weight gain – 5-10lbs
Longest time of return of fertility after discontinuing
Hair loss or thinning
Headaches, Low libido and nausea
Bahamondes, Contraception, 2001
Bonny, Obstet Gyn, 2009

## Slide 27
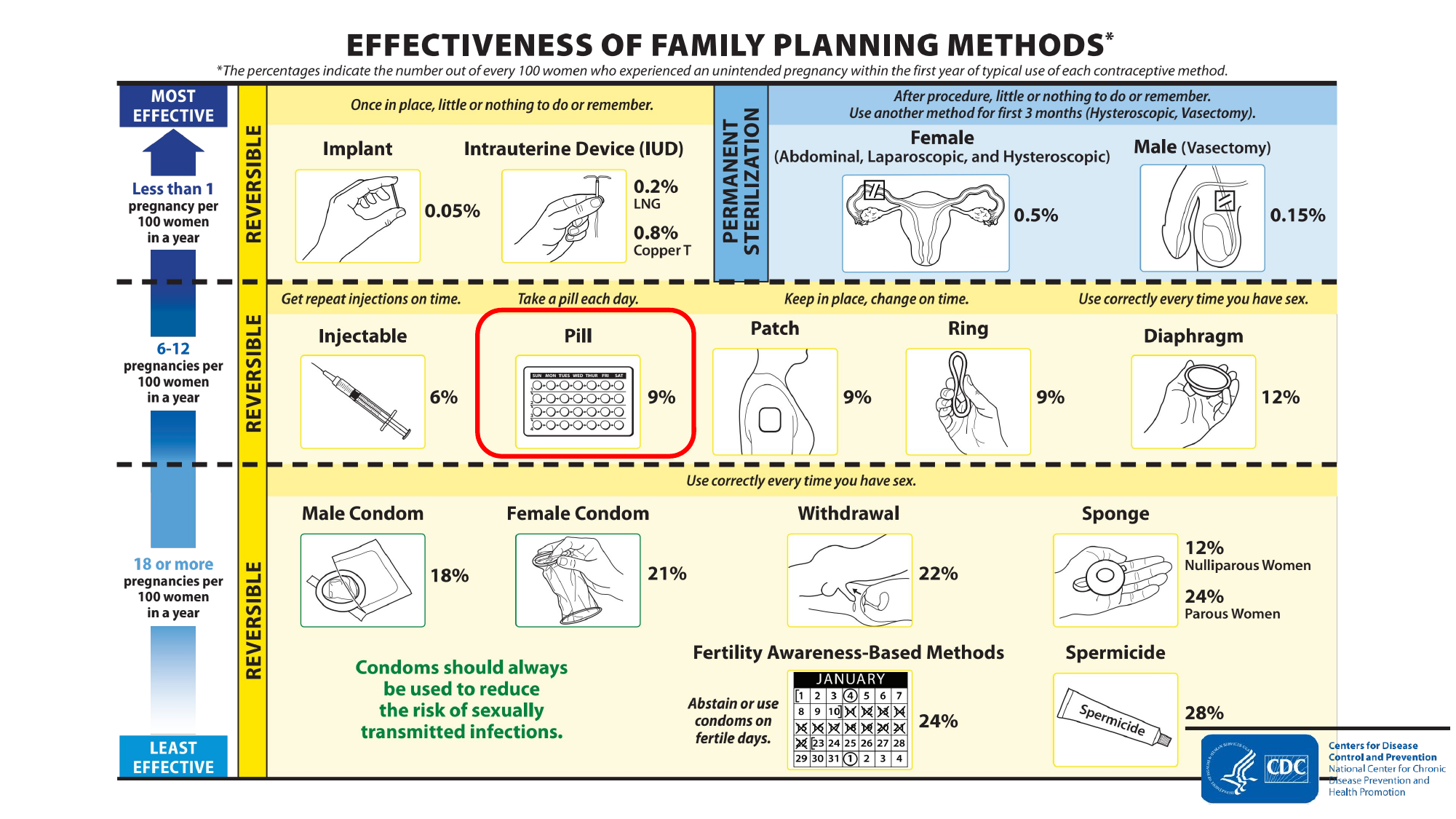

## Slide 28
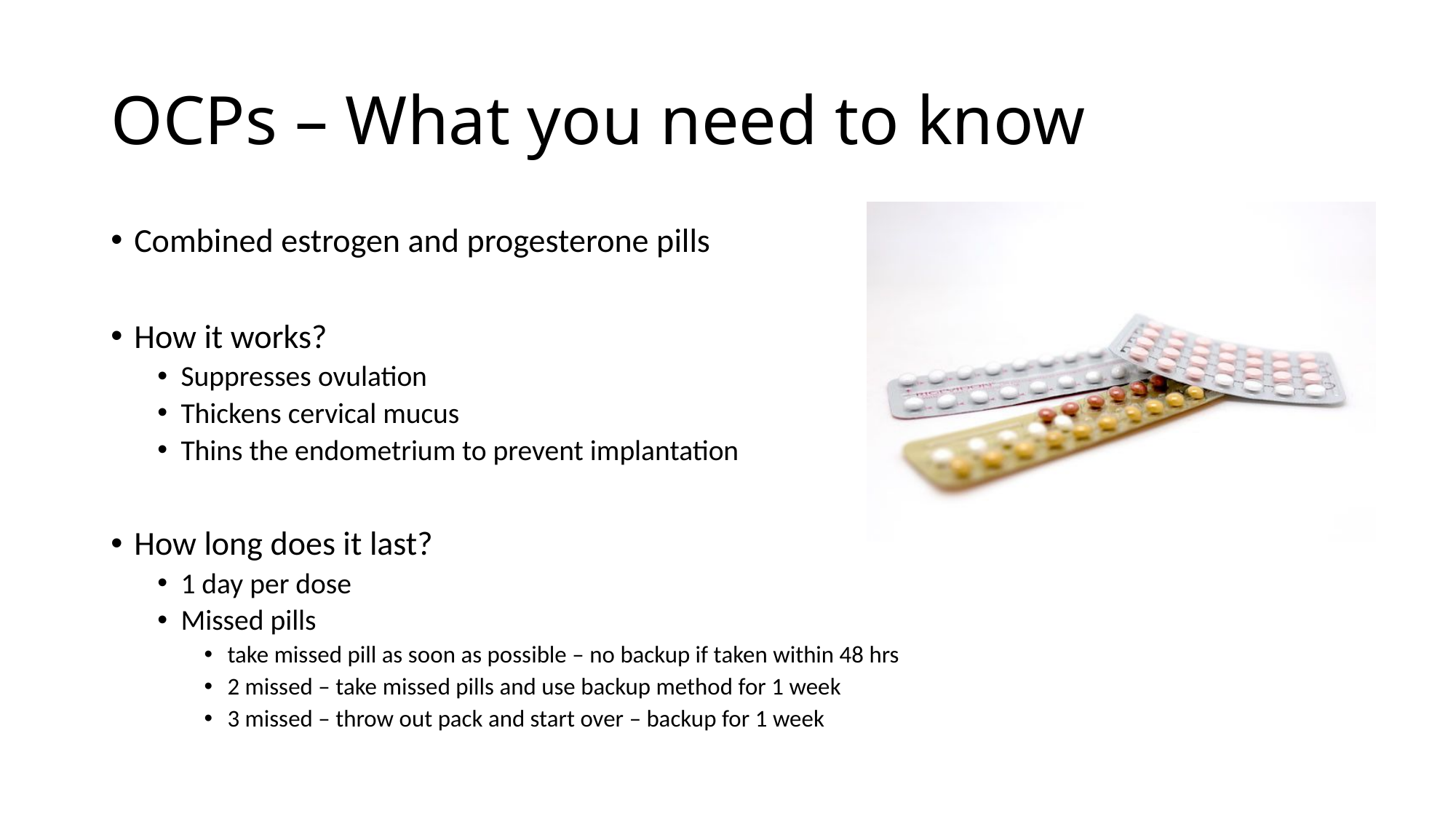

# OCPs – What you need to know
Combined estrogen and progesterone pills
How it works?
Suppresses ovulation
Thickens cervical mucus
Thins the endometrium to prevent implantation
How long does it last?
1 day per dose
Missed pills
take missed pill as soon as possible – no backup if taken within 48 hrs
2 missed – take missed pills and use backup method for 1 week
3 missed – throw out pack and start over – backup for 1 week

## Slide 29
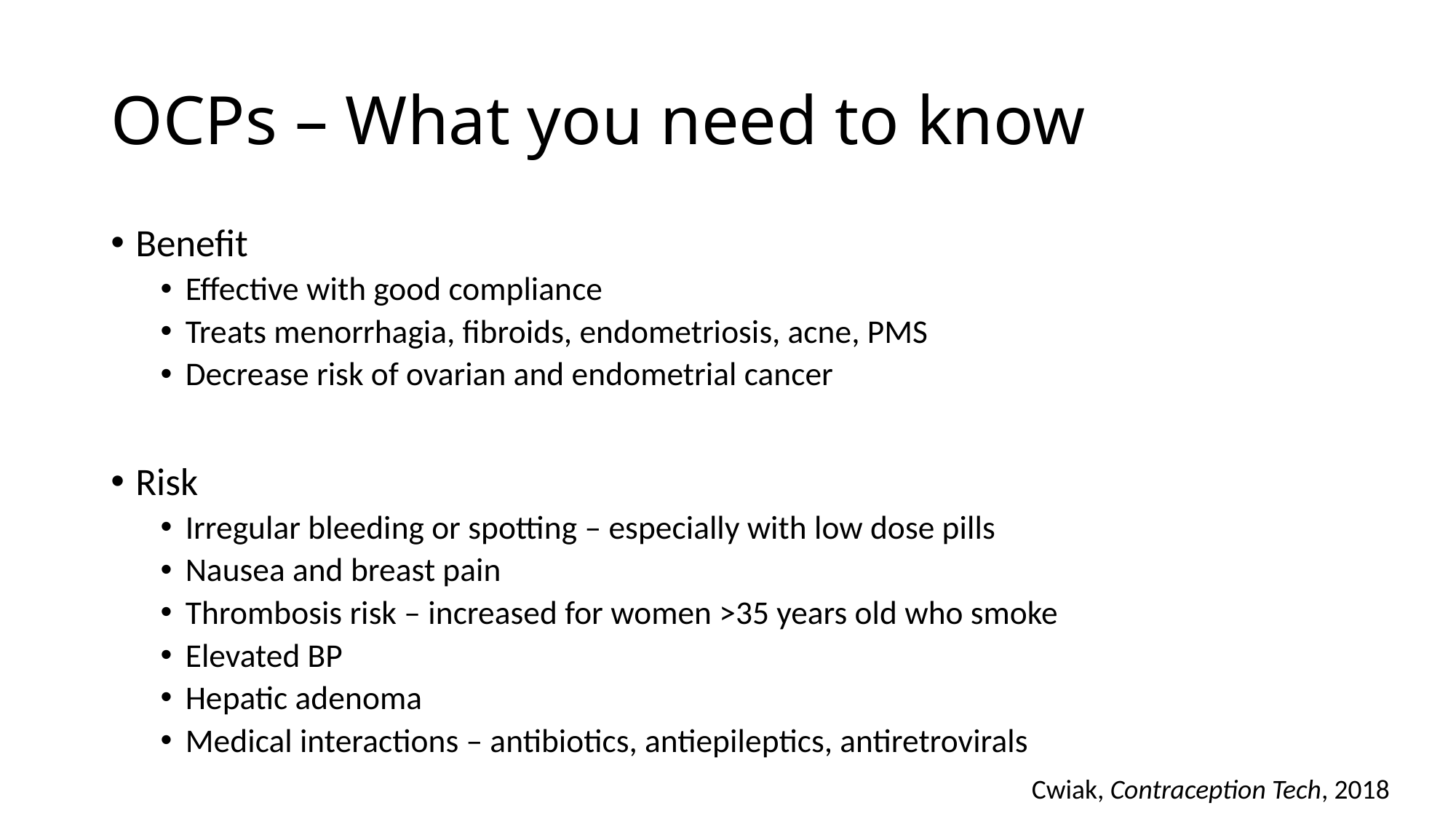

# OCPs – What you need to know
Benefit
Effective with good compliance
Treats menorrhagia, fibroids, endometriosis, acne, PMS
Decrease risk of ovarian and endometrial cancer
Risk
Irregular bleeding or spotting – especially with low dose pills
Nausea and breast pain
Thrombosis risk – increased for women >35 years old who smoke
Elevated BP
Hepatic adenoma
Medical interactions – antibiotics, antiepileptics, antiretrovirals
Cwiak, Contraception Tech, 2018

## Slide 30
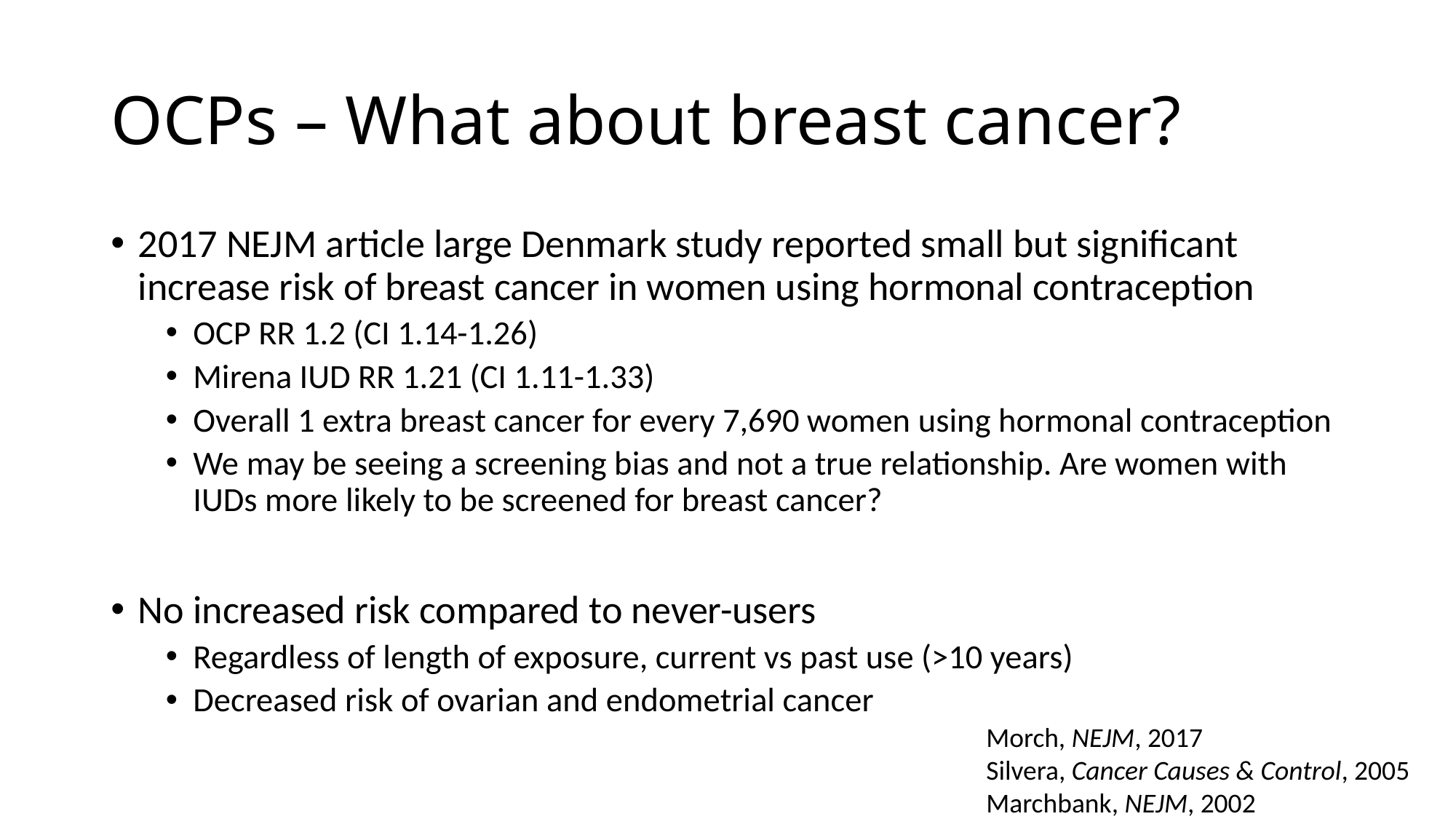

# OCPs – What about breast cancer?
2017 NEJM article large Denmark study reported small but significant increase risk of breast cancer in women using hormonal contraception
OCP RR 1.2 (CI 1.14-1.26)
Mirena IUD RR 1.21 (CI 1.11-1.33)
Overall 1 extra breast cancer for every 7,690 women using hormonal contraception
We may be seeing a screening bias and not a true relationship. Are women with IUDs more likely to be screened for breast cancer?
No increased risk compared to never-users
Regardless of length of exposure, current vs past use (>10 years)
Decreased risk of ovarian and endometrial cancer
Morch, NEJM, 2017
Silvera, Cancer Causes & Control, 2005
Marchbank, NEJM, 2002

## Slide 31
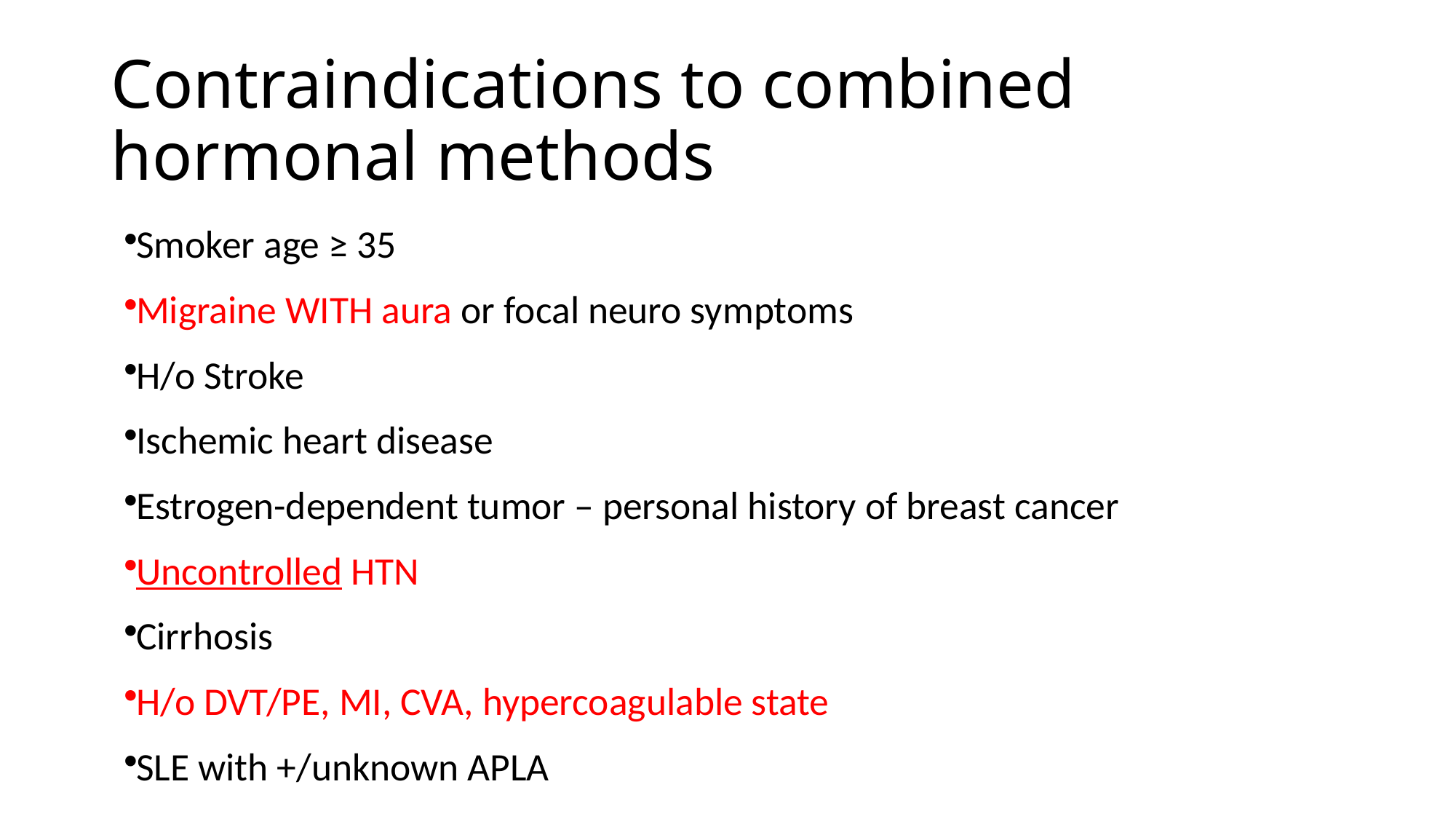

# Contraindications to combined hormonal methods
Smoker age ≥ 35
Migraine WITH aura or focal neuro symptoms
H/o Stroke
Ischemic heart disease
Estrogen-dependent tumor – personal history of breast cancer
Uncontrolled HTN
Cirrhosis
H/o DVT/PE, MI, CVA, hypercoagulable state
SLE with +/unknown APLA

## Slide 32
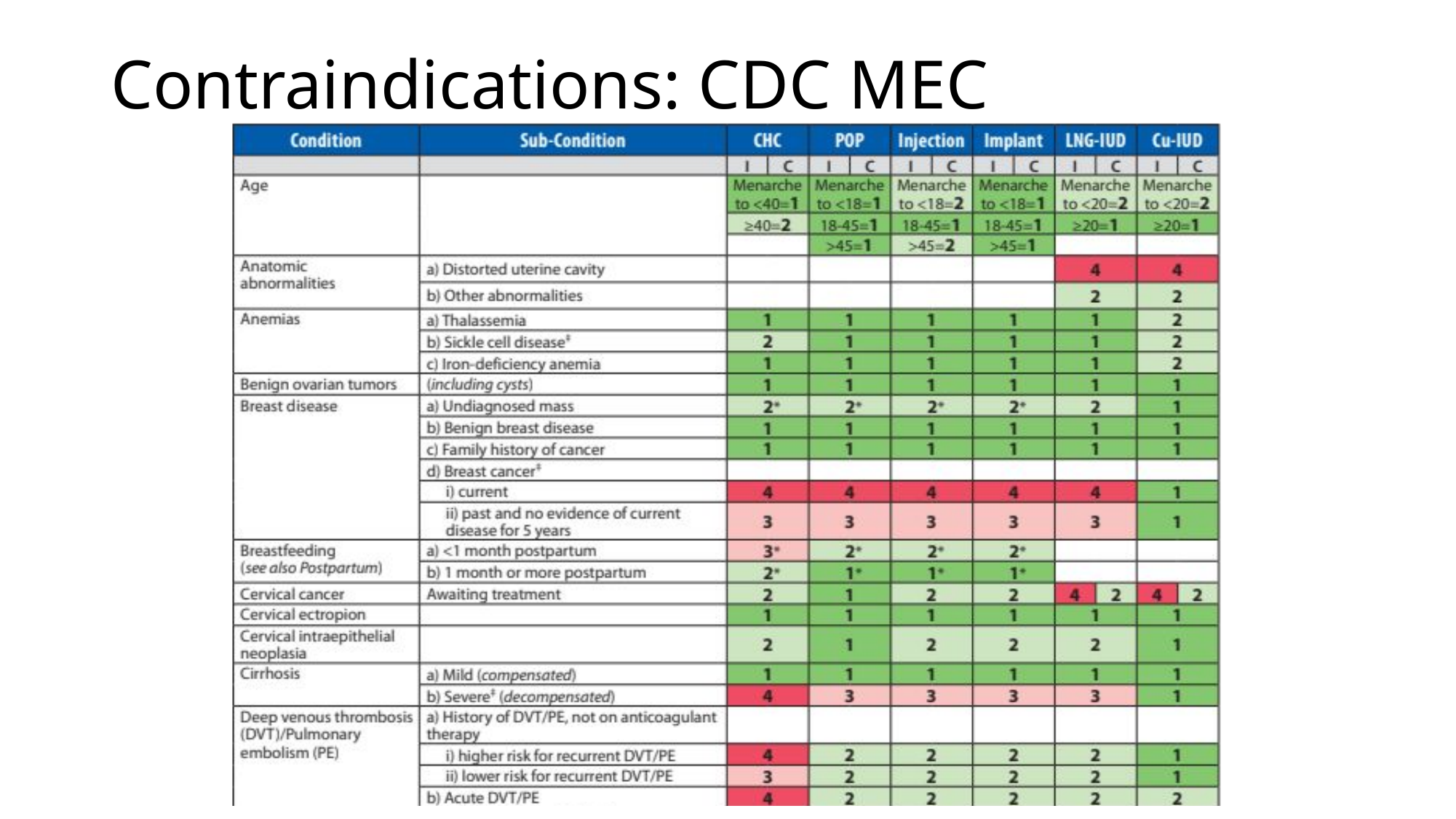

# Contraindications: CDC MEC

## Slide 33
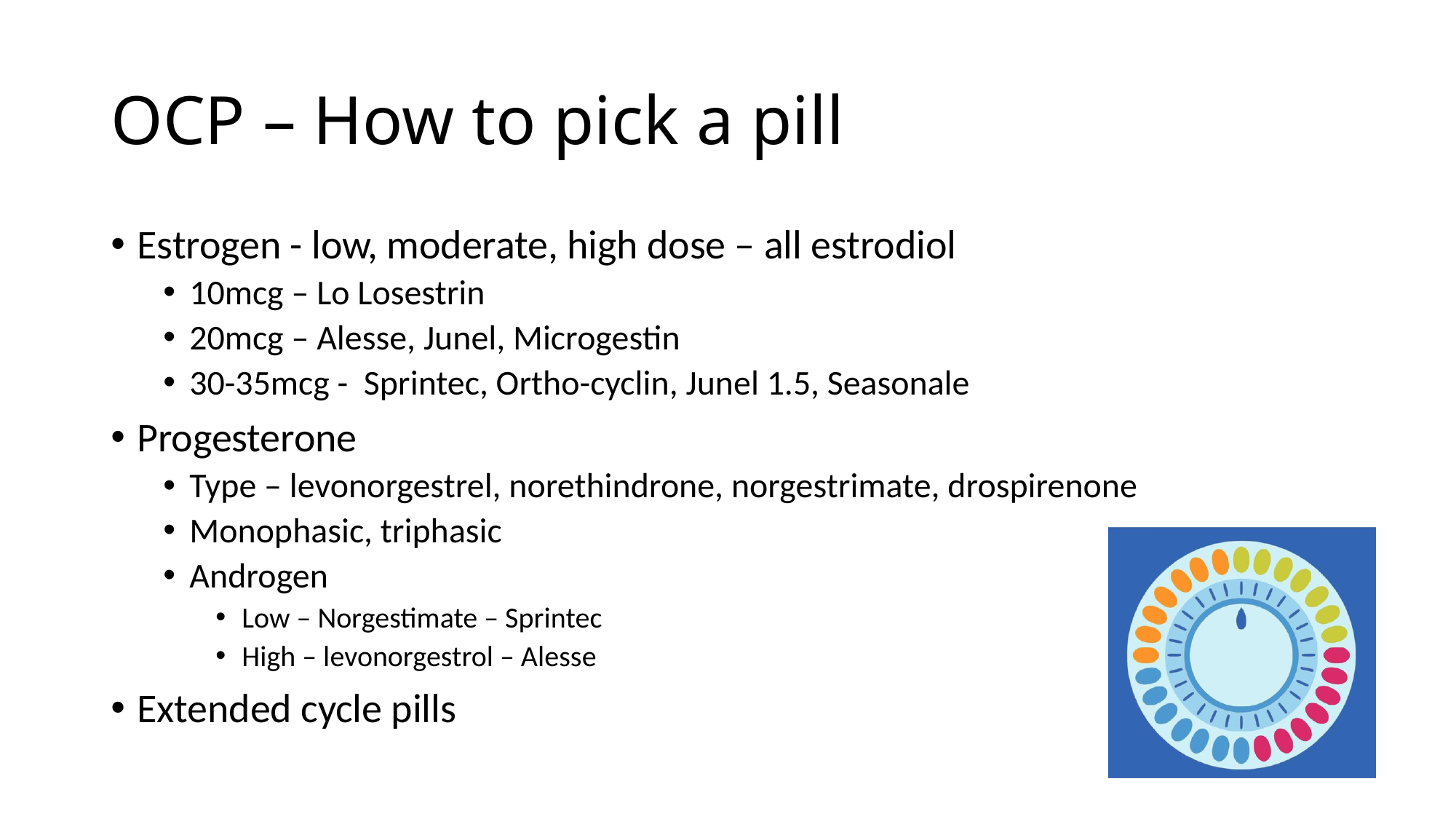

# OCP – How to pick a pill
Estrogen - low, moderate, high dose – all estrodiol
10mcg – Lo Losestrin
20mcg – Alesse, Junel, Microgestin
30-35mcg - Sprintec, Ortho-cyclin, Junel 1.5, Seasonale
Progesterone
Type – levonorgestrel, norethindrone, norgestrimate, drospirenone
Monophasic, triphasic
Androgen
Low – Norgestimate – Sprintec
High – levonorgestrol – Alesse
Extended cycle pills

## Slide 34
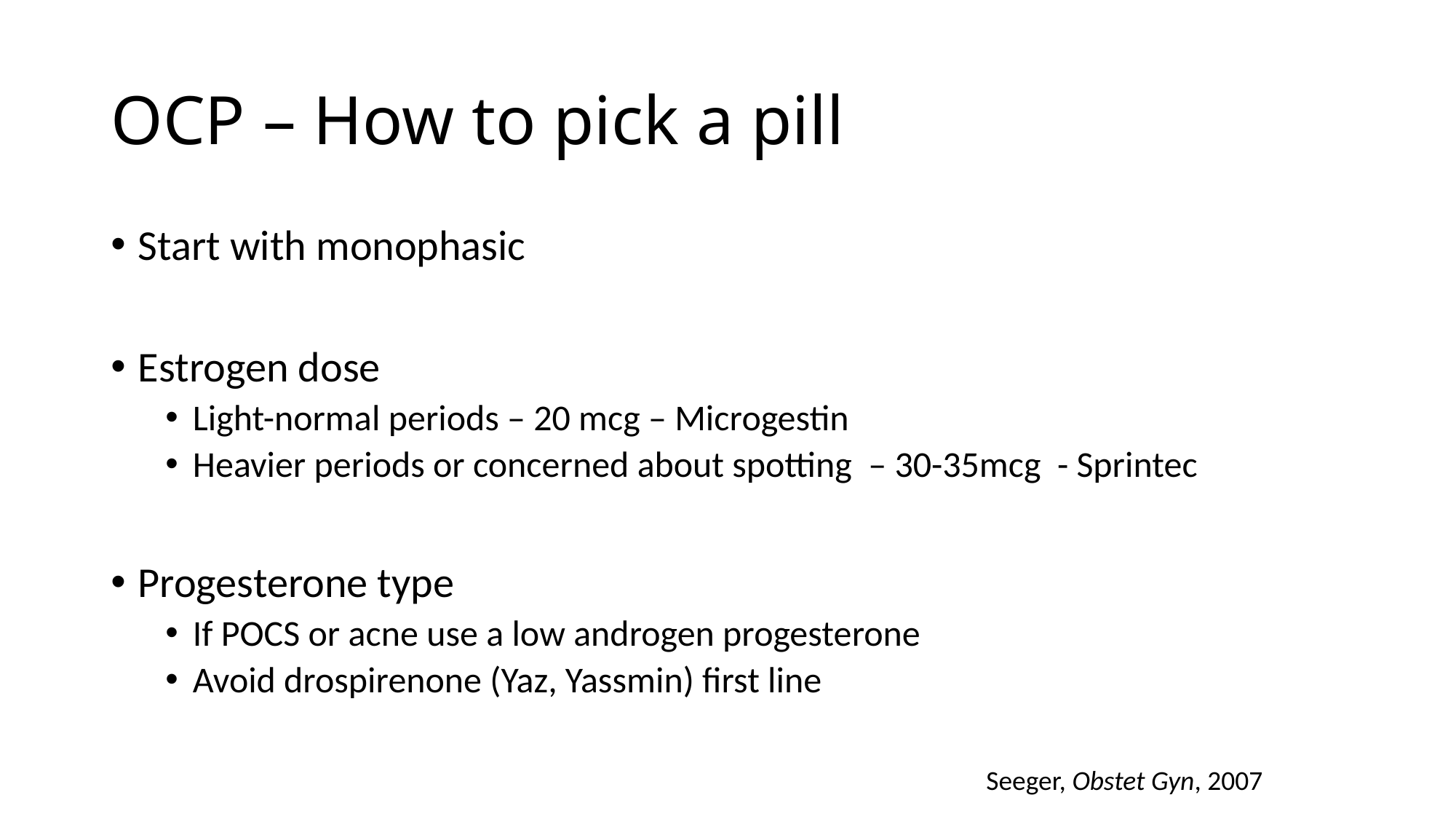

# OCP – How to pick a pill
Start with monophasic
Estrogen dose
Light-normal periods – 20 mcg – Microgestin
Heavier periods or concerned about spotting – 30-35mcg - Sprintec
Progesterone type
If POCS or acne use a low androgen progesterone
Avoid drospirenone (Yaz, Yassmin) first line
Seeger, Obstet Gyn, 2007

## Slide 35
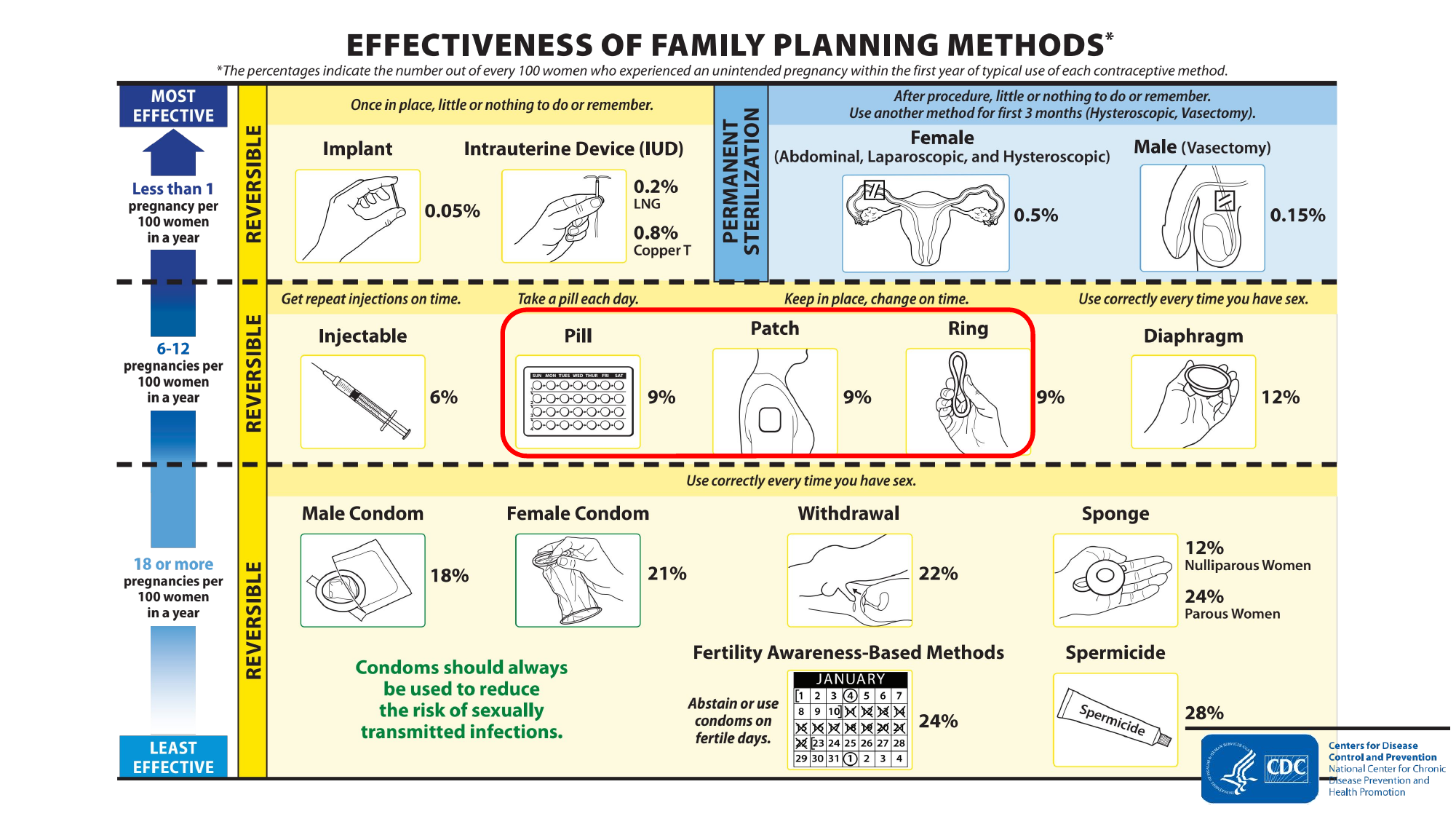

## Slide 36
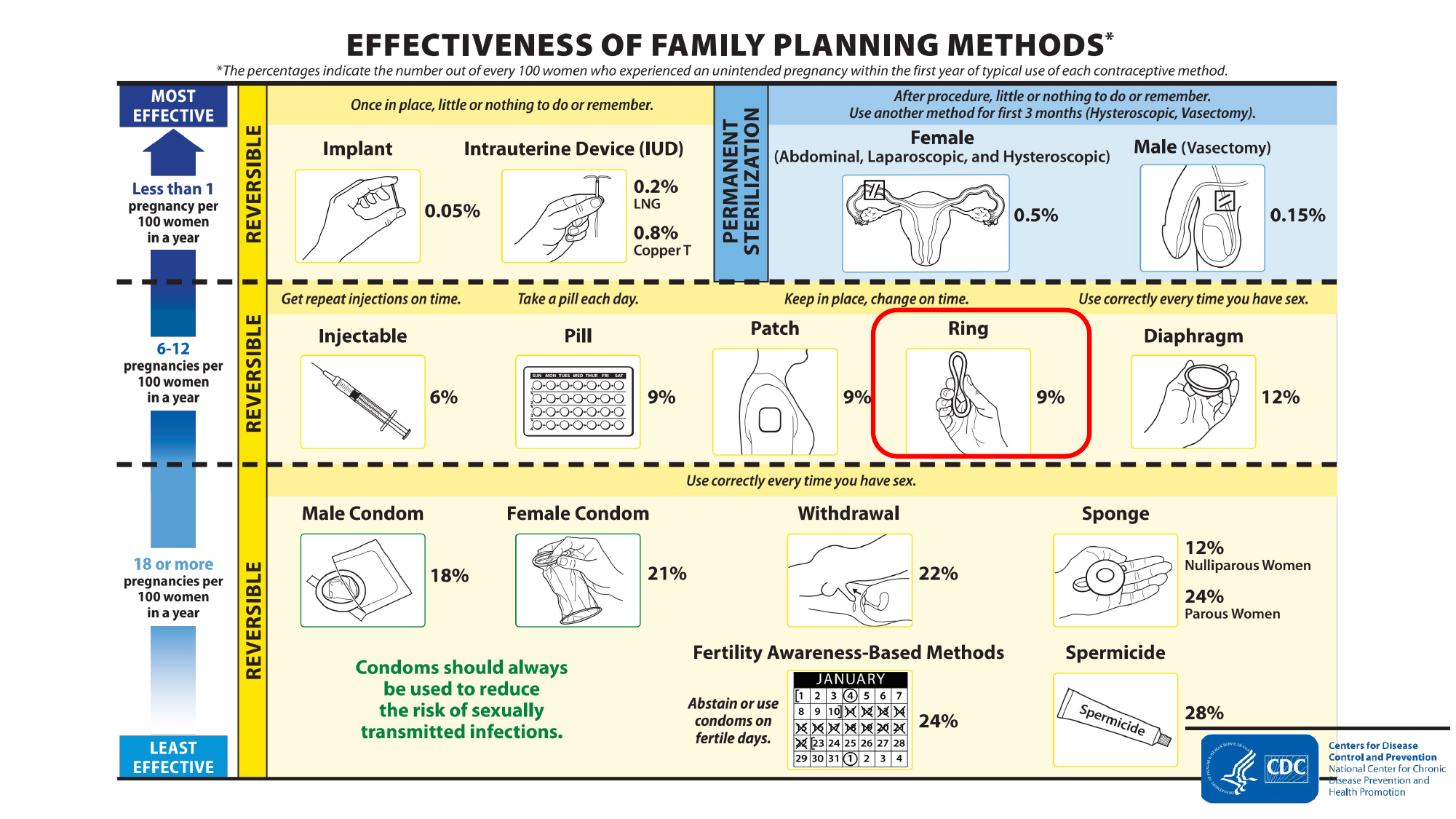

## Slide 37
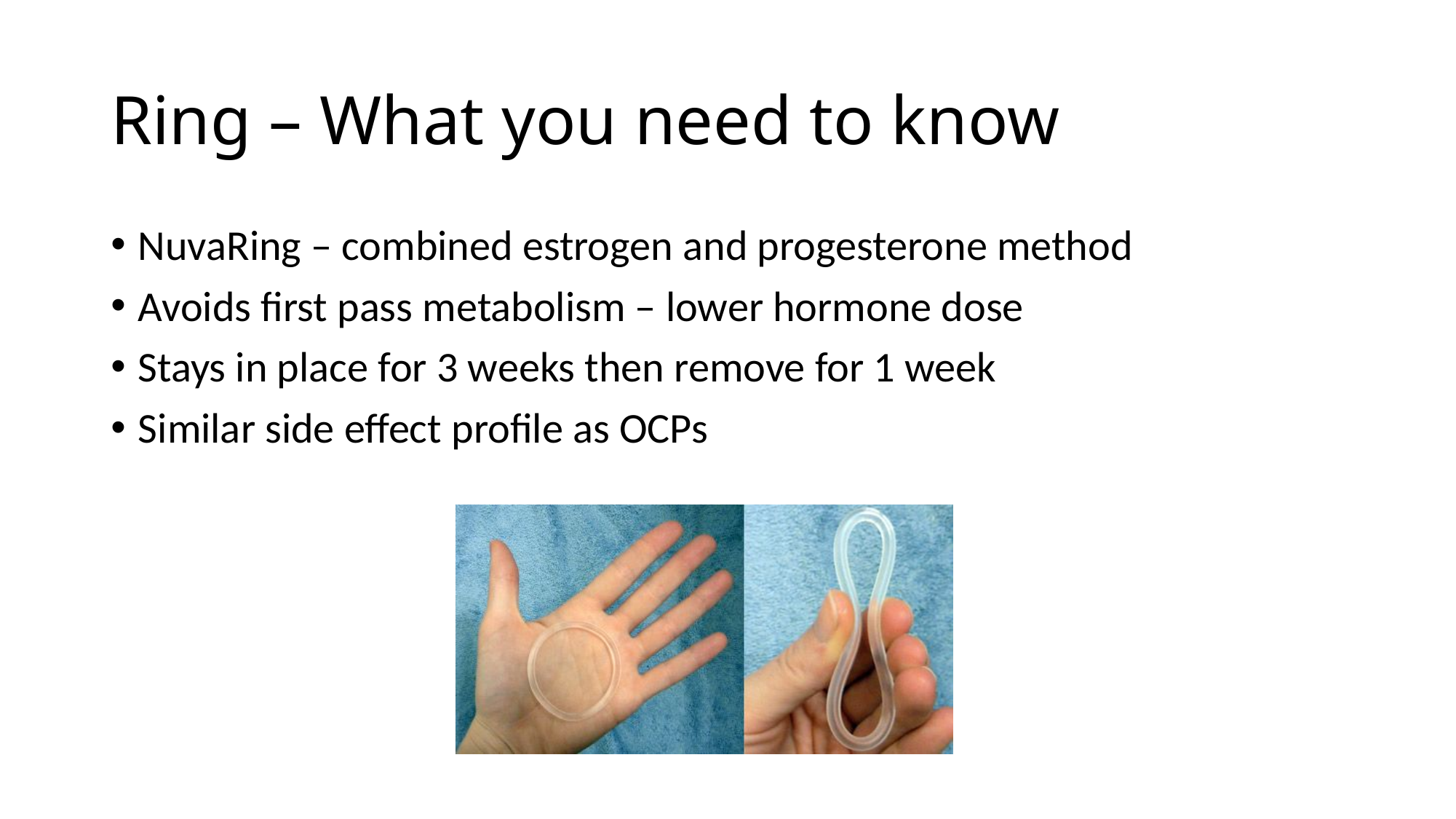

# Ring – What you need to know
NuvaRing – combined estrogen and progesterone method
Avoids first pass metabolism – lower hormone dose
Stays in place for 3 weeks then remove for 1 week
Similar side effect profile as OCPs

## Slide 38
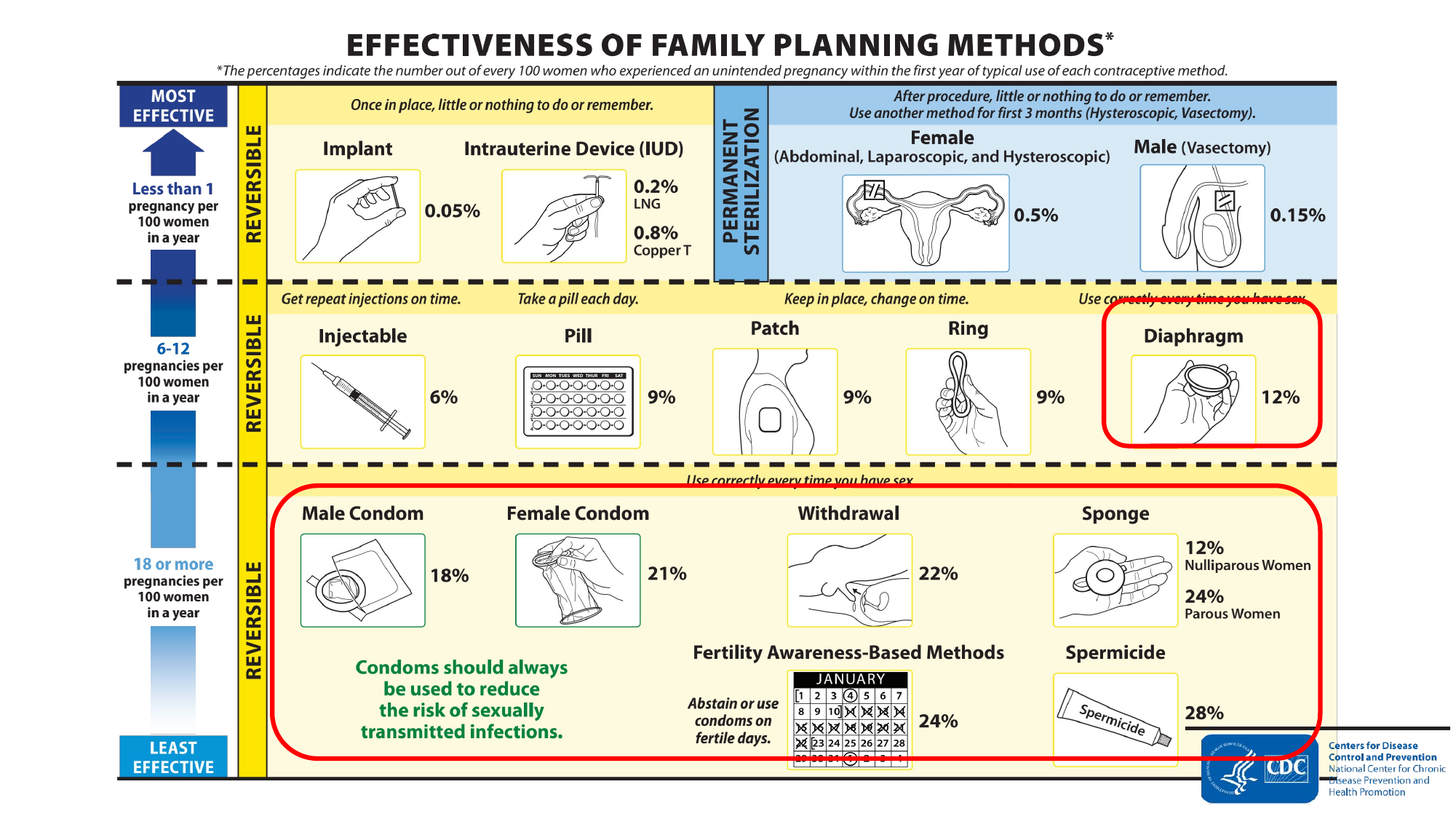

## Slide 39
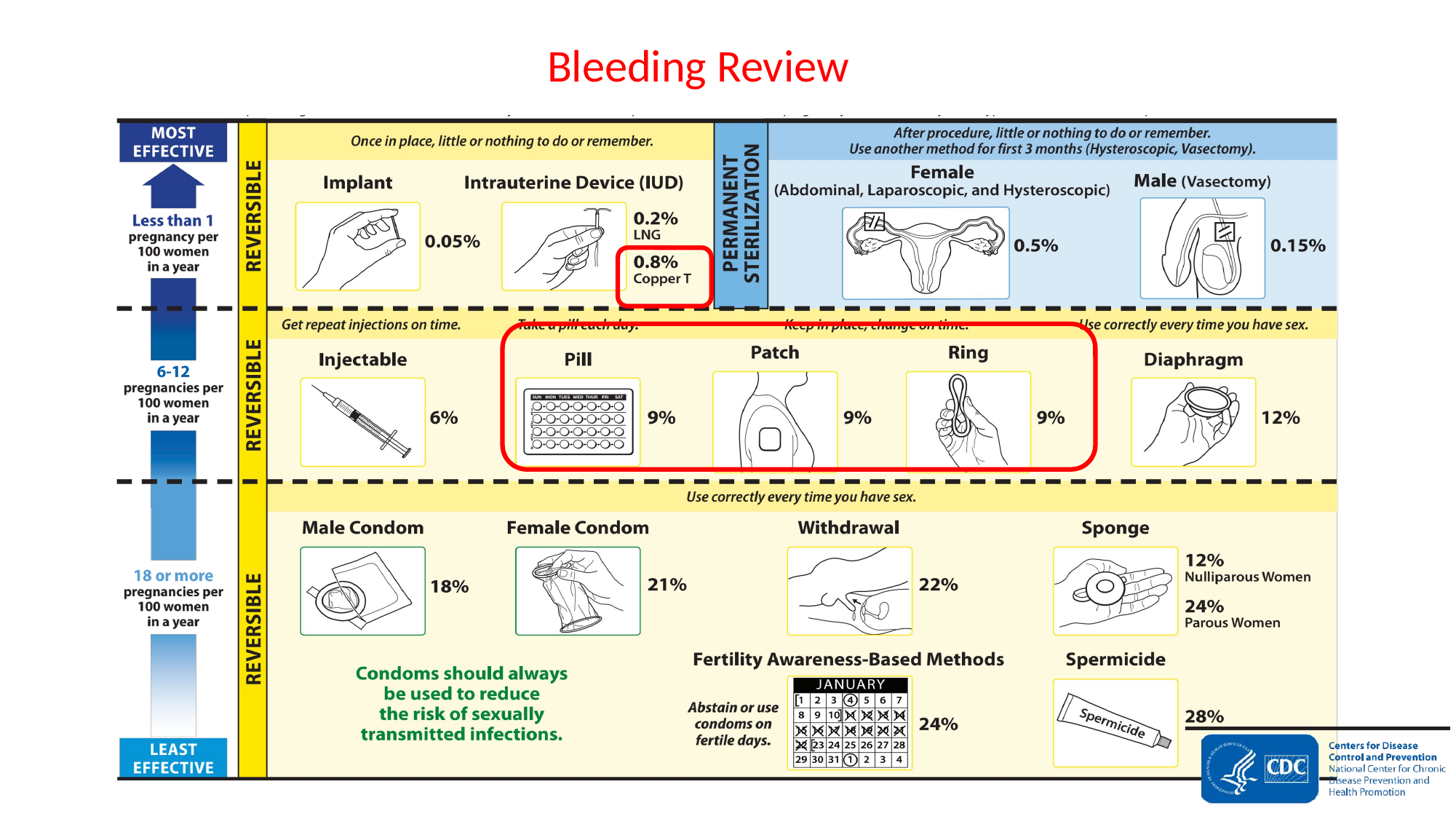

Bleeding Review

## Slide 40
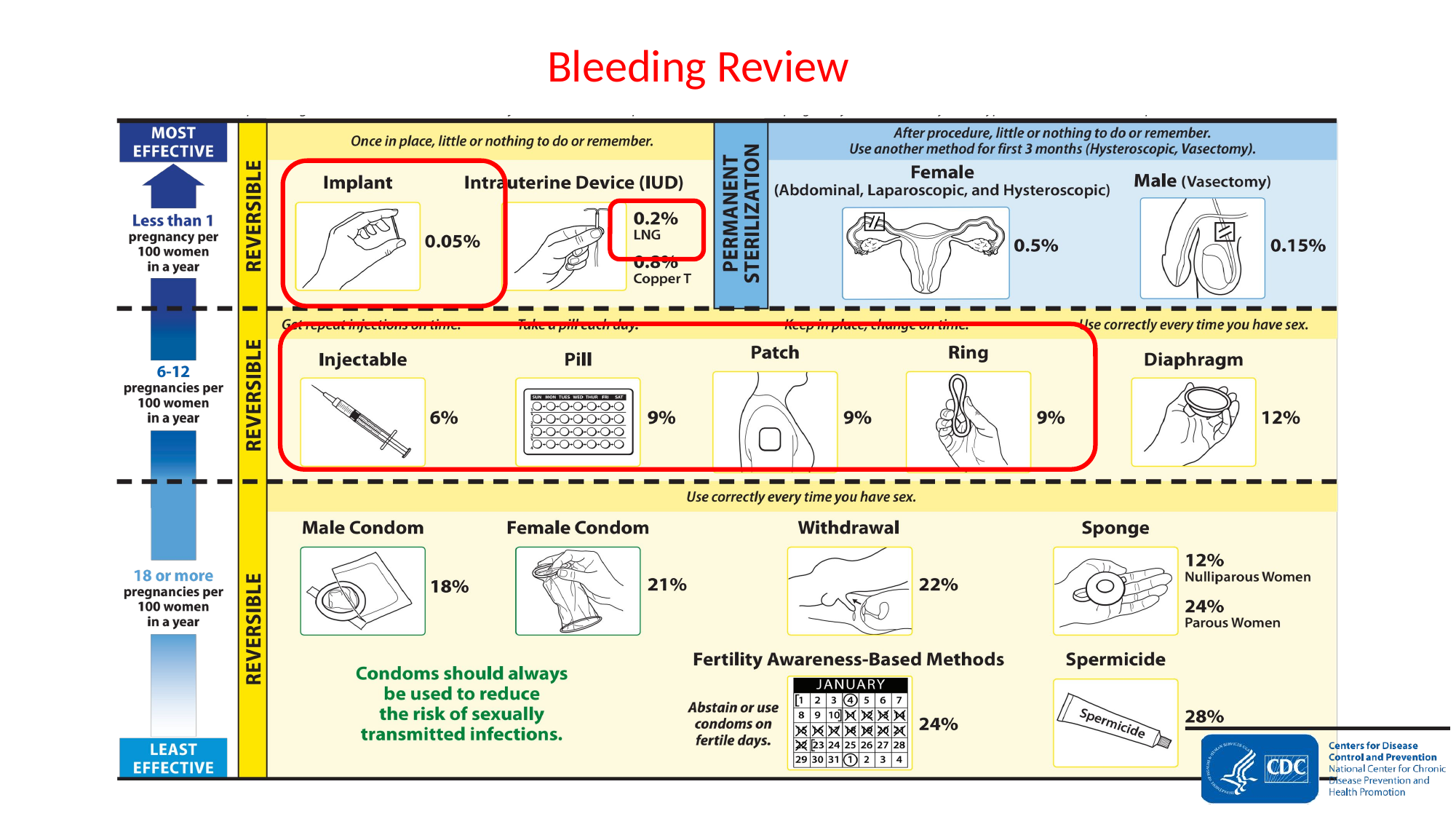

Bleeding Review

## Slide 41
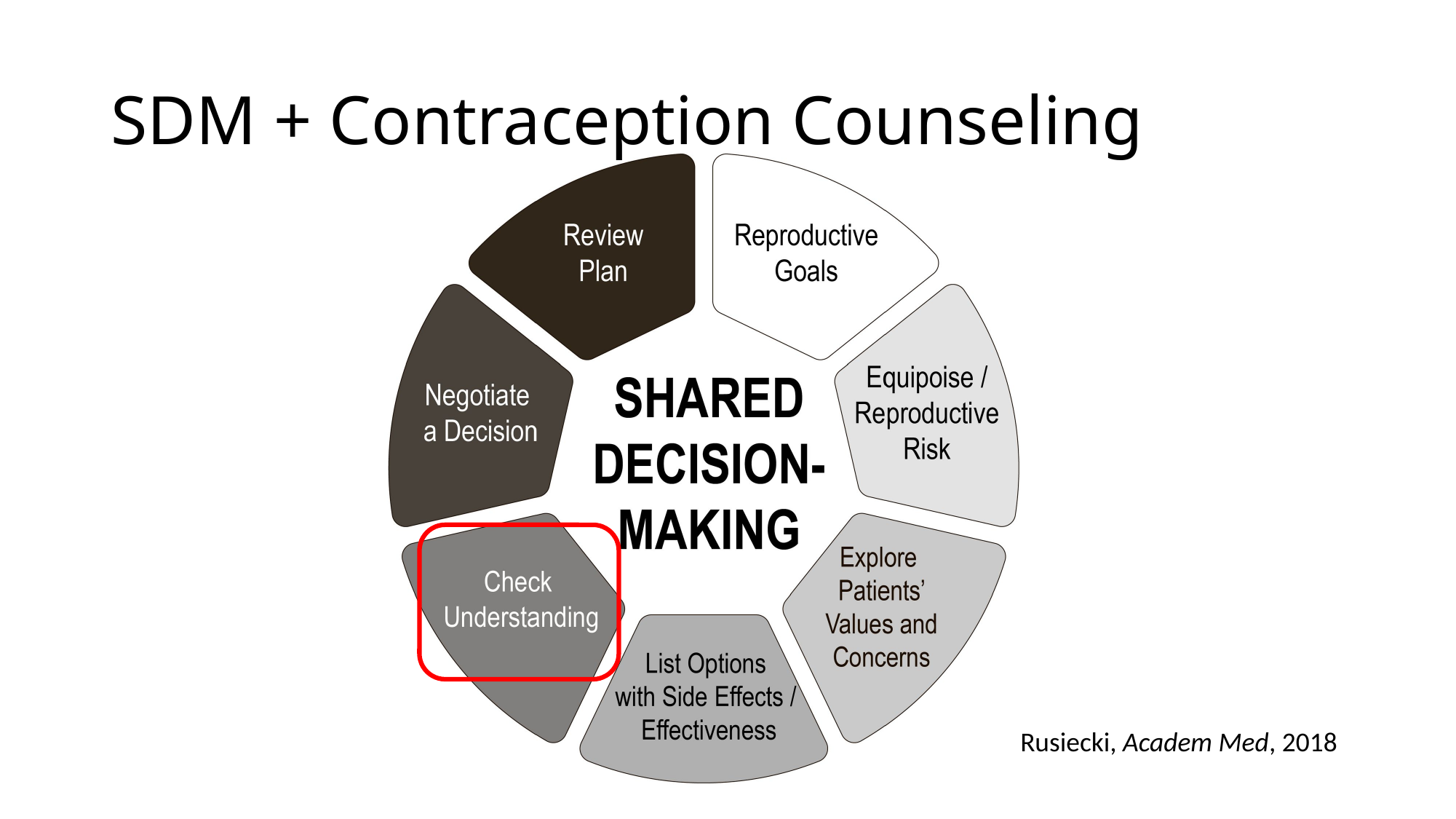

# SDM + Contraception Counseling
Rusiecki, Academ Med, 2018

## Slide 42
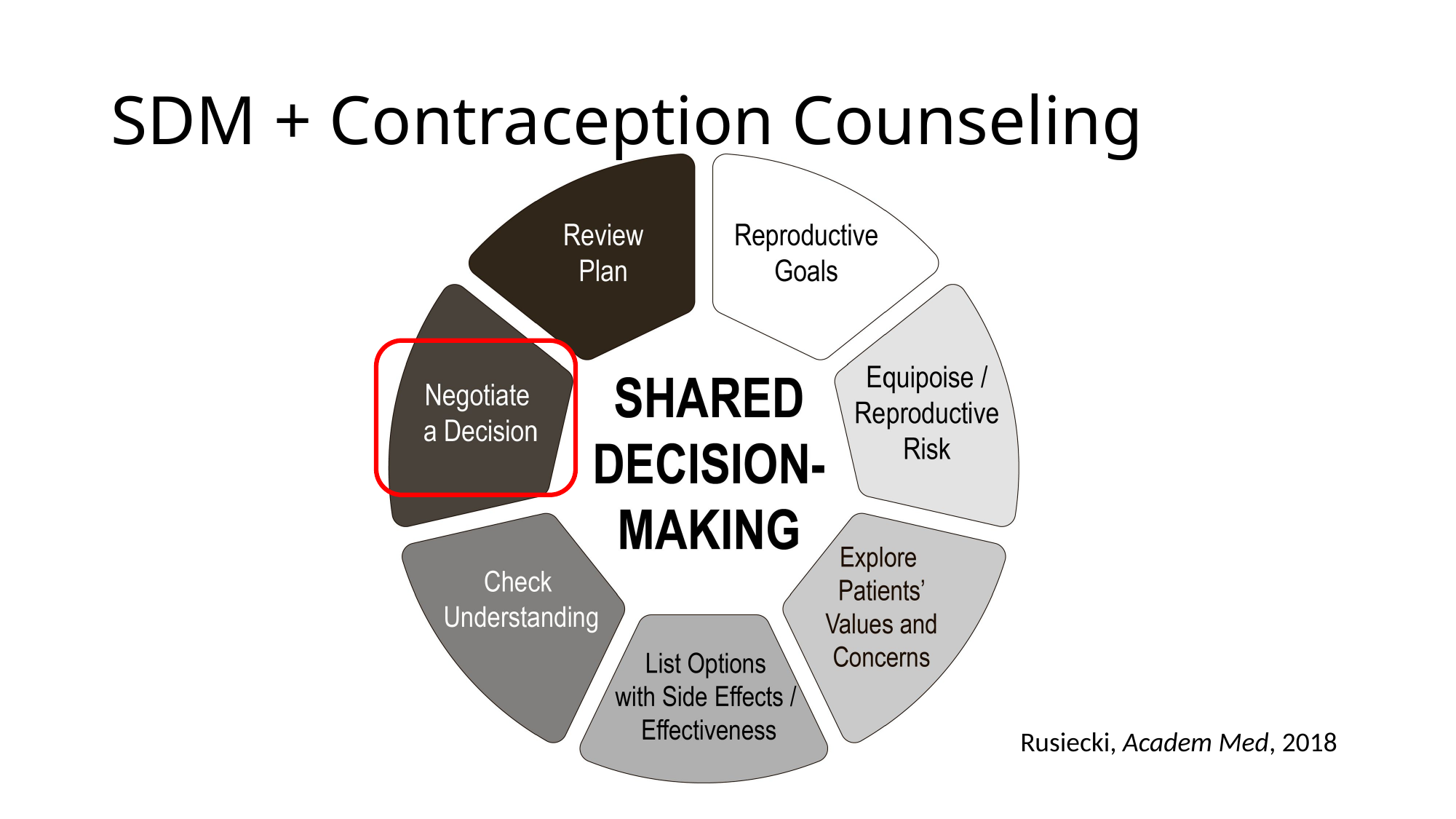

# SDM + Contraception Counseling
Rusiecki, Academ Med, 2018

## Slide 43
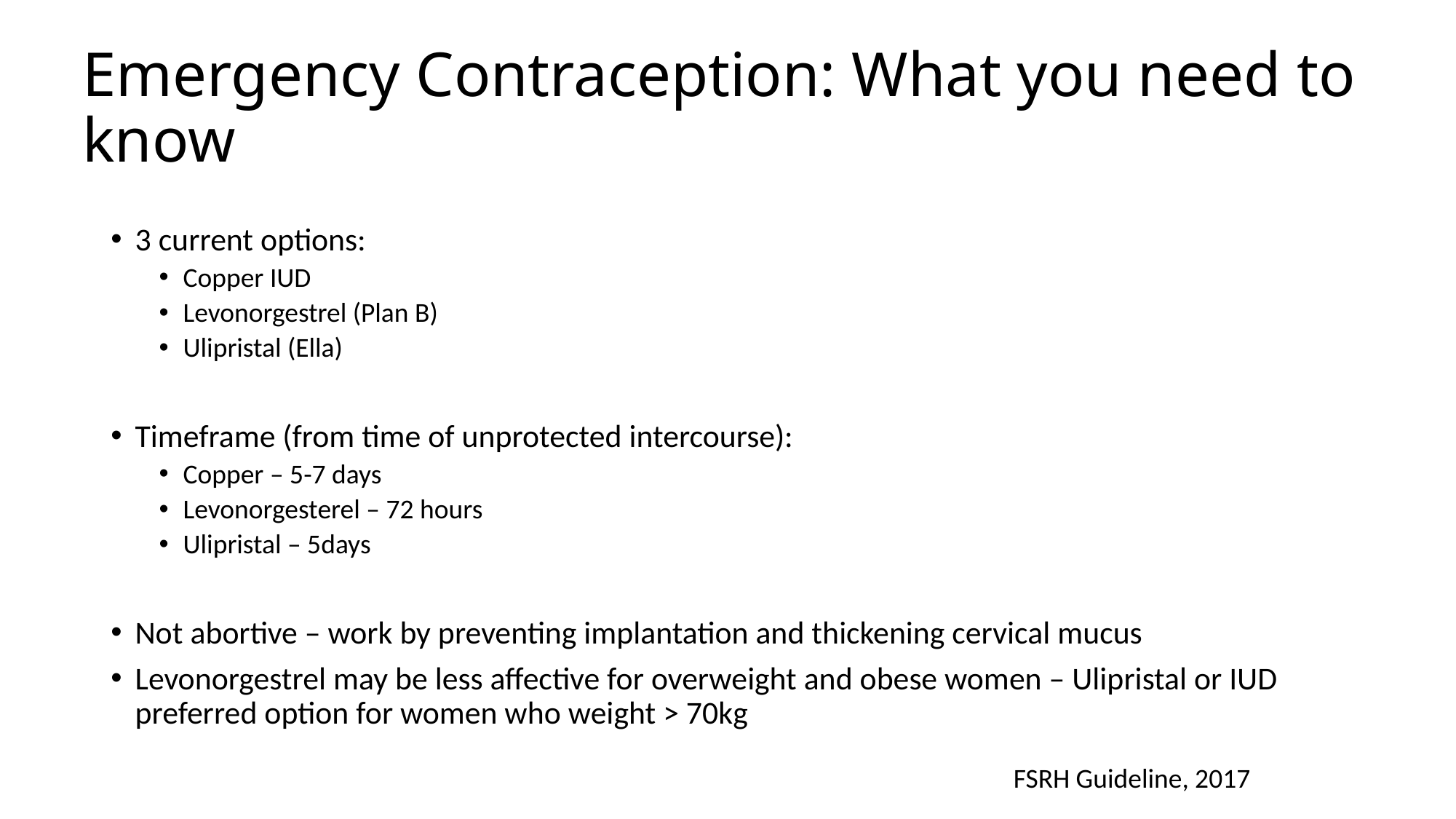

# Emergency Contraception: What you need to know
3 current options:
Copper IUD
Levonorgestrel (Plan B)
Ulipristal (Ella)
Timeframe (from time of unprotected intercourse):
Copper – 5-7 days
Levonorgesterel – 72 hours
Ulipristal – 5days
Not abortive – work by preventing implantation and thickening cervical mucus
Levonorgestrel may be less affective for overweight and obese women – Ulipristal or IUD preferred option for women who weight > 70kg
FSRH Guideline, 2017

## Slide 44
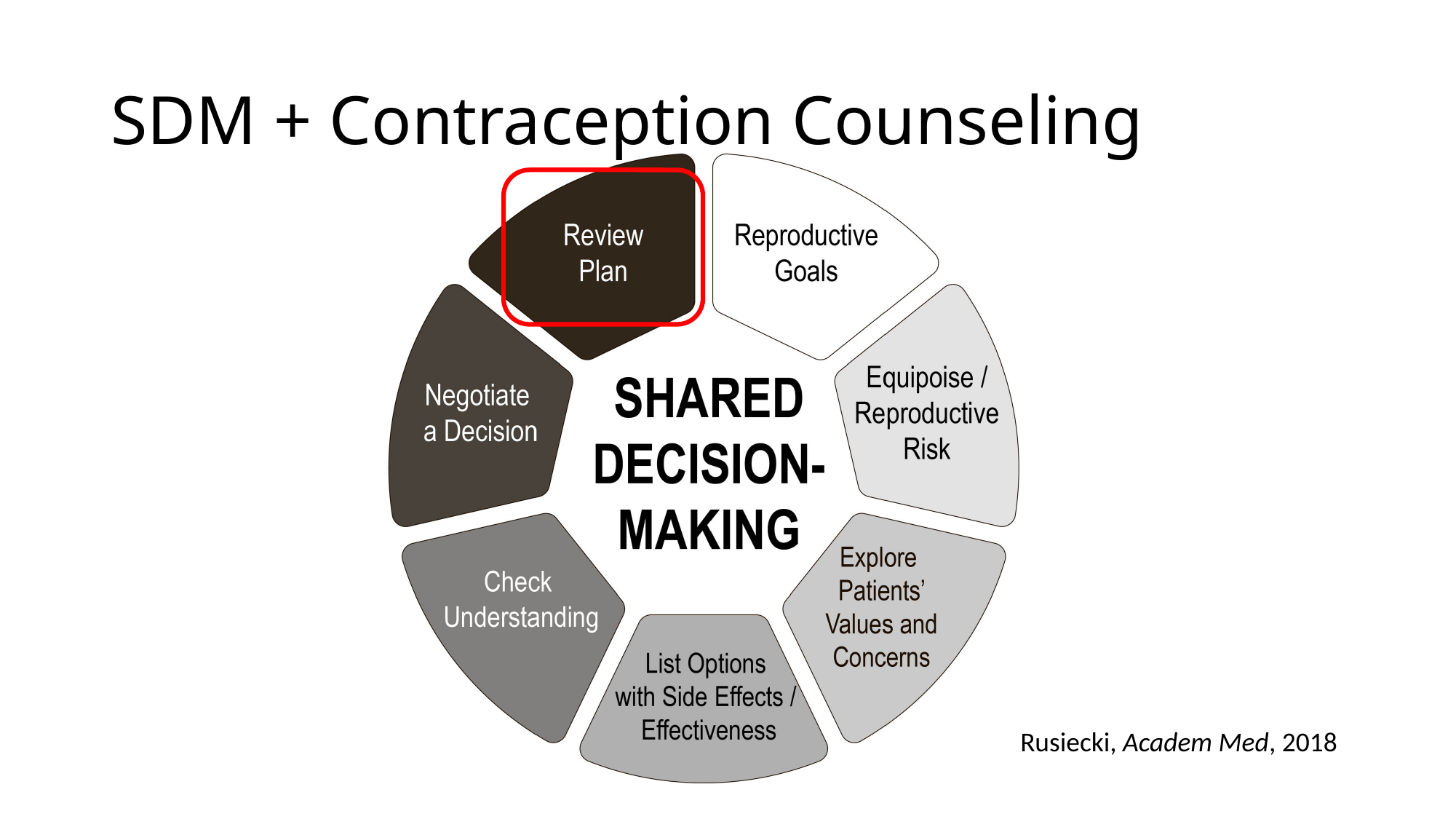

# SDM + Contraception Counseling
Rusiecki, Academ Med, 2018

## Slide 45
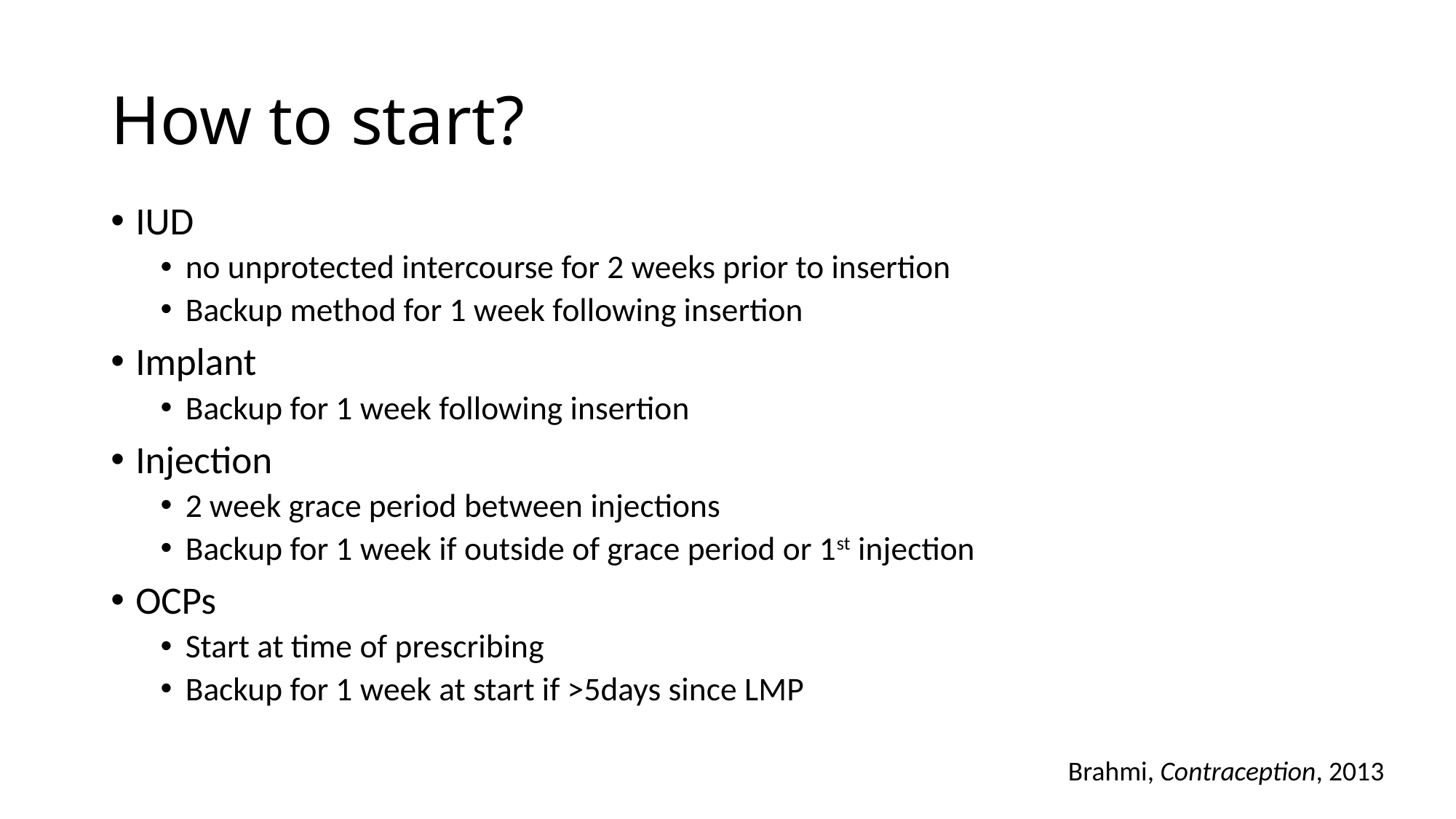

# How to start?
IUD
no unprotected intercourse for 2 weeks prior to insertion
Backup method for 1 week following insertion
Implant
Backup for 1 week following insertion
Injection
2 week grace period between injections
Backup for 1 week if outside of grace period or 1st injection
OCPs
Start at time of prescribing
Backup for 1 week at start if >5days since LMP
Brahmi, Contraception, 2013

## Slide 46
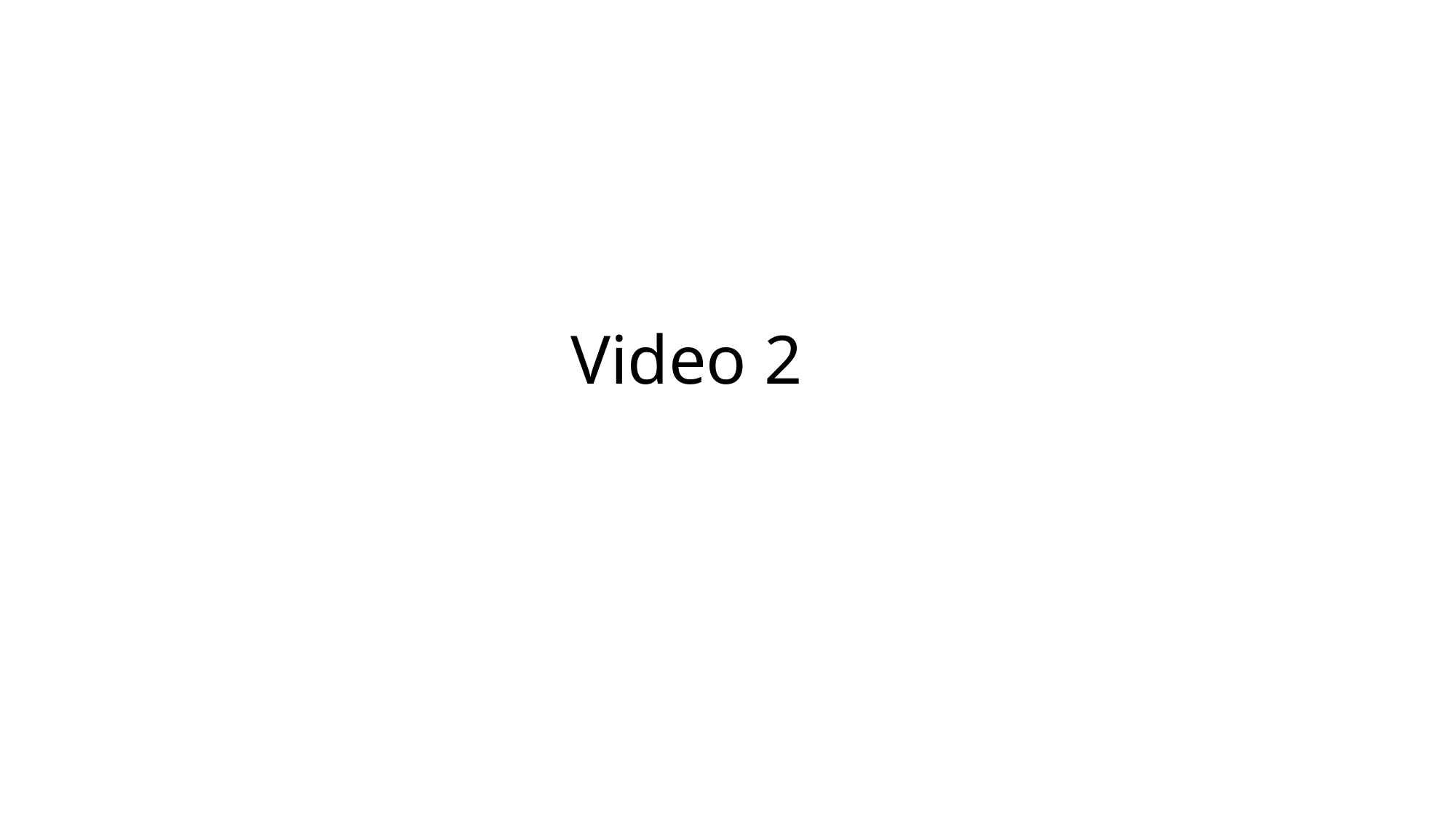

# Video 2

## Slide 47
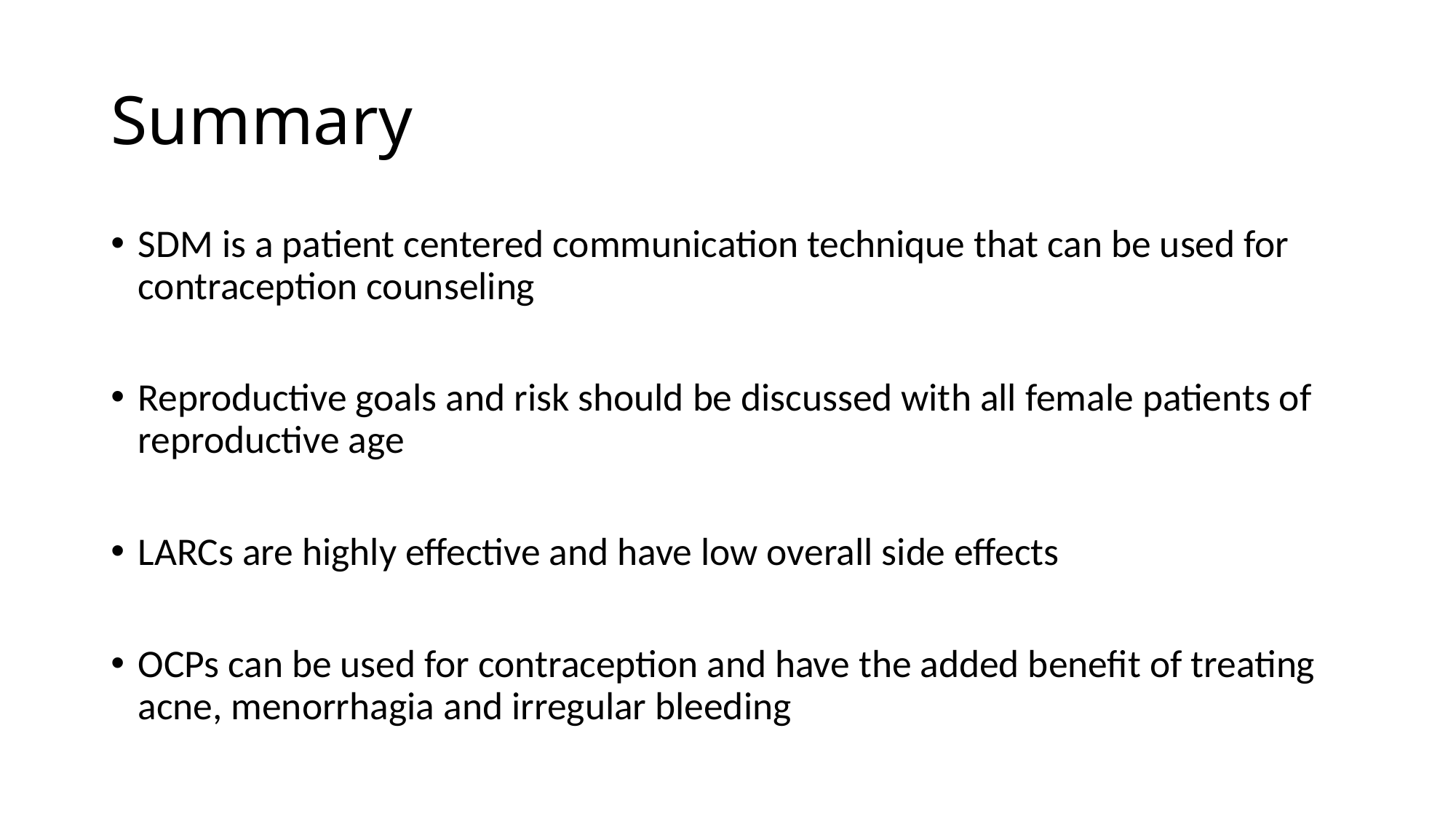

# Summary
SDM is a patient centered communication technique that can be used for contraception counseling
Reproductive goals and risk should be discussed with all female patients of reproductive age
LARCs are highly effective and have low overall side effects
OCPs can be used for contraception and have the added benefit of treating acne, menorrhagia and irregular bleeding
